# Supplementary material for: Developing indicators of risk to environmental variability based on species dependency in U.S. fishing communities in the Northeast and Southeast Regions
Source: PLoS One. 2025 Dec 30;20(12):e0335034. doi: 10.1371/journal.pone.0335034 (PMC12752975; doi:10.1371/journal.pone.0335034)
Supplement: S1 File — (PDF) [file pone.0335034.s001.pdf]

Supplemental Materials I

S1. Table 1. Sensitivity attributes. List of all sensitivity attributes used in CVAs in all three Regions studied - Northeast, South Atlantic, and Gulf of America/Florida Keys.

**Sensitivity Attributes**
Habitat Specificity
Prey Specificity
Sensitivity to Ocean Acidification
Complexity in Reproductive Strategy
Sensitivity to Temperature
Early Life History Survival and Settlement Requirements
Stock Size/Status
Other Stressors
Population Growth Rate
Dispersal of Early Life Stages
Adult Mobility
Spawning Cycle

S1. Table 2. Exposure Factors. List of all exposure factors used in CVAs in all three Regions studied - Northeast, South Atlantic, and Gulf of America/Florida Keys.

**Exposure Factors**	**Northeast**	**South Atlantic**	**Gulf of America/Florida Keys**
Mean Ocean Surface Temperature	X	X	X
Mean Ocean Surface Salinity	X	X	X
Mean Air Temperature	X	X	X
Mean Precipitation	X	X	X
Mean Ocean pH	X	X	X
Sea Level Rise	X	X	X
Variability in Ocean Surface Temperature	X		
Variability in Ocean Surface Salinity	X		
Variability in Air Temperature	X		
Variability in Precipitation	X		
Variability in pH	X		
Ocean Currents	X		
Gulf Stream-Induced Upwelling		X	
Hypoxia			X
Primary Productivity			X
