# Supplementary material for: Developing indicators of risk to environmental variability based on species dependency in U.S. fishing communities in the Northeast and Southeast Regions
Source: PLoS One. 2025 Dec 30;20(12):e0335034. doi: 10.1371/journal.pone.0335034 (PMC12752975; doi:10.1371/journal.pone.0335034)
Supplement: S2 File — (PDF) [file pone.0335034.s002.pdf]

Supplemental Materials II

S2 Table 1. Northeast Region communities. Five-year averages (2018-2022) for all coastal fishing communities in the Northeast Region with any landings of species with CVAs, including region, community name, state, percent classified species in terms of value, sensitivity to temperature, stock size/status, ocean acidification, total sensitivity and total vulnerability, regional quotient for pounds and value, and Simpsons' Reciprocal diversity scores for value.

Region	COMMUNITY	ST	% Class	Temp	Stock	OA	TSens	TVuln	RQLb	RQVal	DVal
NE	BRANFORD	CT	0.43	1.74	2.21	2.14	1.98	2.80	0.00	0.00	2.75
NE	BRIDGEPORT	CT	0.63	1.86	2.21	3.33	2.86	3.78	0.00	0.00	1.91
NE	CLINTON	CT	0.66	1.96	2.17	2.22	2.11	2.58	0.00	0.00	2.48
NE	DARIEN	CT	1.00	2.32	2.28	2.12	2.00	2.00	0.00	0.00	1.00
NE	EAST HAVEN	CT	0.97	1.44	1.89	1.55	1.29	1.99	0.00	0.00	4.92
NE	EAST LYME	CT	1.00	2.08	2.17	1.86	2.34	2.76	0.00	0.00	1.41
NE	FAIRFIELD	CT	1.00	1.48	2.44	1.76	1.00	2.00	0.00	0.00	1.00
NE	GLASTONBURY	CT	1.00	1.32	2.72	1.12	2.00	3.00	0.00	0.00	1.00
NE	GREENWICH	CT	0.93	1.85	2.16	2.22	1.99	2.36	0.00	0.00	1.49
NE	GROTON	CT	0.89	2.18	2.25	2.28	2.14	2.33	0.00	0.00	1.68
NE	GUILFORD	CT	0.50	1.48	2.03	1.96	1.64	2.56	0.00	0.00	2.84
NE	HADDAM	CT	1.00	1.56	2.92	1.36	3.00	4.00	0.00	0.00	1.00
NE	HARTFORD	CT	1.00	1.32	2.72	1.12	2.00	3.00	0.00	0.00	1.00
NE	LYME	CT	0.98	1.43	1.87	1.58	1.34	2.24	0.00	0.00	3.24
NE	MILFORD	CT	0.58	1.77	2.16	3.24	2.69	3.66	0.00	0.00	2.08
NE	MONTVILLE	CT	1.00	1.84	2.48	1.72	2.03	2.53	0.00	0.00	1.08
NE	MYSTIC	CT	1.00	1.49	1.90	1.32	1.11	1.58	0.00	0.00	4.66
NE	NEW HAVEN	CT	0.18	1.94	2.21	3.16	2.85	3.76	0.00	0.00	1.79
NE	NEW LONDON	CT	0.92	1.76	1.80	1.96	1.51	1.76	0.00	0.00	5.82
NE	NIANTIC	CT	0.86	1.70	1.99	1.74	1.84	2.67	0.00	0.00	6.15
NE	NOANK	CT	1.00	2.26	2.27	2.11	1.97	2.04	0.00	0.00	1.17
NE	NORWALK	CT	0.85	2.21	2.26	2.34	2.18	2.43	0.00	0.00	2.19
NE	NORWICH	CT	1.00	1.60	2.01	1.90	2.16	3.16	0.00	0.00	1.63
NE	OLD LYME	CT	1.00	2.14	2.28	2.69	2.32	2.66	0.00	0.00	1.01
NE	OLD SAYBROOK	CT	0.93	1.56	2.53	1.53	2.13	3.00	0.00	0.00	3.48
NE	PORTLAND	CT	1.00	1.56	2.92	1.36	3.00	4.00	0.00	0.00	1.00
NE	ROCKY HILL	CT	1.00	1.56	2.92	1.36	3.00	4.00	0.00	0.00	1.01
NE	STAMFORD	CT	1.00	2.16	2.24	2.40	2.13	2.38	0.00	0.00	1.18
NE	STONINGTON	CT	0.97	1.96	1.88	2.53	1.97	2.21	0.00	0.00	4.54
NE	STRATFORD	CT	1.00	1.72	2.25	3.20	2.56	3.51	0.00	0.00	1.48
NE	WATERFORD	CT	1.00	2.06	2.26	2.44	2.39	2.85	0.00	0.00	2.09
NE	WESTBROOK	CT	0.99	2.03	2.28	2.66	2.30	2.72	0.00	0.00	1.85
NE	BOWERS	DE	1.00	1.64	2.66	2.09	3.00	4.00	0.00	0.00	1.95
NE	INDIAN RIVER	DE	0.99	1.73	2.18	2.56	2.48	3.09	0.00	0.00	3.58
NE	LEWES	DE	1.00	1.56	2.14	2.39	2.34	3.28	0.00	0.00	2.43
NE	LITTLE CREEK	DE	1.00	1.62	2.72	1.93	3.00	4.00	0.00	0.00	1.53
NE	MILFORD	DE	0.99	1.66	2.52	2.39	2.99	3.99	0.00	0.00	2.31
NE	AMESBURY	MA	1.00	2.15	2.78	1.24	1.58	1.58	0.00	0.00	1.83
NE	AQUINNAH	MA	1.00	2.39	2.63	3.91	3.98	4.00	0.00	0.00	1.04
NE	BARNSTABLE	MA	0.96	1.76	2.24	3.46	2.78	3.28	0.00	0.01	4.57
NE	BERKLEY	MA	1.00	2.32	2.28	2.12	2.00	2.00	0.00	0.00	1.00
NE	BEVERLY	MA	0.99	2.32	2.28	2.14	2.01	2.01	0.00	0.00	1.05
NE	BOSTON	MA	1.00	2.10	2.28	1.46	1.67	1.69	0.01	0.01	8.29
NE	BOURNE	MA	0.97	1.80	2.27	2.71	2.85	3.31	0.00	0.00	3.59
NE	BREWSTER	MA	0.93	2.02	2.37	2.70	2.33	2.66	0.00	0.00	2.07
NE	CAMBRIDGE	MA	1.00	2.32	2.28	2.12	2.00	2.00	0.00	0.00	1.00

NE	CHATHAM	MA	0.94	2.05	2.08	2.63	2.23	2.41	0.01	0.01	7.11
NE	CHELSEA	MA	1.00	2.32	2.28	2.12	2.00	2.00	0.00	0.00	1.00
NE	CHILMARK	MA	0.97	1.89	2.23	2.77	2.44	2.91	0.00	0.00	5.72
NE	COHASSET	MA	1.00	2.32	2.28	2.12	2.00	2.00	0.00	0.00	1.01
NE	DANVERS	MA	1.00	2.32	2.28	2.11	2.01	2.01	0.00	0.00	1.01
NE	DARTMOUTH	MA	1.00	1.65	2.15	2.45	2.47	3.33	0.00	0.00	3.11
NE	DENNIS	MA	0.86	1.68	2.40	3.26	2.75	3.42	0.00	0.00	3.37
NE	DUXBURY	MA	0.98	1.41	2.55	3.86	3.00	3.99	0.00	0.00	1.09
NE	EASTHAM	MA	0.96	1.66	2.42	3.63	3.27	3.75	0.00	0.00	2.86
NE	EDGARTOWN	MA	1.00	1.67	2.47	3.78	3.13	3.92	0.00	0.00	2.73
NE	ESSEX	MA	0.94	1.82	2.28	3.45	3.00	3.99	0.00	0.00	1.17
NE	FAIRHAVEN	MA	0.94	2.17	1.99	3.81	2.95	3.22	0.00	0.00	2.91
NE	FALL RIVER	MA	0.87	1.65	2.15	1.82	1.69	2.42	0.00	0.00	2.18
NE	FALMOUTH	MA	0.96	1.57	2.29	3.36	3.08	3.54	0.00	0.00	3.46
NE	FREETOWN	MA	1.00	1.68	1.96	1.24	3.00	4.00	0.00	0.00	1.00
NE	GLOUCESTER	MA	0.96	2.15	2.23	1.88	1.81	1.87	0.04	0.03	5.29
NE	GOSNOLD	MA	1.00	1.40	2.56	3.87	3.00	4.00	0.00	0.00	1.00
NE	GREEN HARBOR-CEDAR CREST	MA	0.04	1.30	1.49	1.25	0.98	1.23	0.00	0.00	1.13
NE	HARWICH PORT	MA	0.90	2.26	2.03	2.92	2.41	2.54	0.00	0.00	4.07
NE	HINGHAM	MA	1.00	2.29	2.28	2.18	2.05	2.11	0.00	0.00	1.14
NE	HULL	MA	0.99	2.10	2.32	2.03	1.74	2.00	0.00	0.00	1.48
NE	IPSWICH	MA	0.80	1.86	2.27	3.37	2.93	3.85	0.00	0.00	1.73
NE	KINGSTON	MA	0.63	1.64	2.48	3.41	2.75	3.51	0.00	0.00	2.51
NE	LYNN	MA	0.81	1.73	1.98	1.34	2.98	3.96	0.00	0.00	1.69
NE	MANCHESTER-BY-THE-SEA	MA	1.00	1.84	2.37	1.91	1.43	2.00	0.00	0.00	1.82
NE	MARBLEHEAD	MA	1.00	2.30	2.28	2.11	2.03	2.05	0.00	0.00	1.09
NE	MARION	MA	1.00	1.66	2.16	3.28	2.70	3.65	0.00	0.00	3.11
NE	MARSHFIELD	MA	0.92	2.28	2.29	2.11	2.03	2.04	0.00	0.00	1.39
NE	MASHPEE	MA	1.00	1.76	2.33	3.31	3.20	3.61	0.00	0.00	2.39
NE	MATTAPOISETT	MA	0.99	1.48	2.25	2.87	2.51	3.51	0.00	0.00	2.07
NE	NAHANT	MA	1.00	2.32	2.27	2.14	2.01	2.01	0.00	0.00	1.03
NE	NANTUCKET	MA	0.99	2.05	2.57	3.81	3.53	3.90	0.00	0.00	2.39
NE	NEW BEDFORD	MA	0.98	2.38	1.85	3.71	2.85	2.86	0.09	0.21	1.47
NE	NEWBURY	MA	0.94	1.85	2.28	3.42	2.96	3.90	0.00	0.00	1.29
NE	NEWBURYPORT	MA	0.83	2.18	2.15	2.24	2.39	2.63	0.00	0.00	3.60
NE	OAK BLUFFS	MA	0.99	1.67	2.27	3.47	2.89	3.77	0.00	0.00	2.71
NE	OCEAN BLUFF-BRANT ROCK	MA	1.00	2.32	2.28	2.12	2.00	2.00	0.00	0.00	1.00
NE	ONSET	MA	1.00	1.85	2.29	3.26	3.04	3.39	0.00	0.00	2.20
NE	ORLEANS	MA	0.89	1.93	2.31	2.96	2.85	3.21	0.00	0.00	4.86
NE	PEABODY	MA	1.00	2.00	2.12	1.68	2.50	3.00	0.00	0.00	1.00
NE	PLYMOUTH	MA	0.94	2.04	2.34	2.62	2.31	2.61	0.00	0.00	2.21
NE	PROVINCETOWN	MA	0.96	2.33	2.07	2.96	2.46	2.47	0.00	0.00	2.38
NE	QUINCY	MA	0.80	1.95	2.26	2.98	2.72	3.44	0.00	0.00	1.91
NE	REVERE	MA	1.00	1.86	2.05	1.52	2.73	3.46	0.00	0.00	1.64
NE	ROCKPORT	MA	1.00	2.32	2.28	2.13	2.01	2.01	0.00	0.00	1.02
NE	ROWLEY	MA	0.93	1.82	2.28	3.48	3.00	4.00	0.00	0.00	1.17
NE	SALEM	MA	0.99	2.32	2.28	2.12	2.00	2.01	0.00	0.00	1.03
NE	SALISBURY	MA	1.00	2.21	2.27	2.44	2.23	2.45	0.00	0.00	1.11
NE	SANDWICH	MA	0.67	2.20	2.22	2.10	2.22	2.40	0.00	0.00	2.78
NE	SAUGUS	MA	1.00	2.31	2.27	2.10	2.02	2.03	0.00	0.00	1.04
NE	SCITUATE	MA	0.96	2.27	2.36	2.05	2.04	2.10	0.00	0.00	1.63
NE	SOMERSET	MA	1.00	1.68	2.28	3.80	4.00	4.00	0.00	0.00	1.00
NE	SWAMPSCOTT	MA	1.00	2.32	2.28	2.11	2.01	2.01	0.00	0.00	1.02
NE	SWANSEA	MA	1.00	1.68	2.28	3.80	4.00	4.00	0.00	0.00	1.00
NE	TISBURY	MA	0.98	1.83	2.25	3.22	3.50	3.68	0.00	0.00	2.45

NE	TRURO	MA	0.92	2.00	2.24	2.64	2.51	2.89	0.00	0.00	2.68
NE	VINEYARD HAVEN	MA	0.99	1.78	2.25	3.53	2.87	3.80	0.00	0.00	1.68
NE	WAREHAM	MA	0.99	1.46	2.53	3.85	3.08	4.00	0.00	0.00	1.37
NE	WELLFLEET	MA	0.98	1.54	2.46	3.82	3.16	3.89	0.00	0.00	1.88
NE	WEST TISBURY	MA	0.98	1.79	2.25	3.05	2.92	3.90	0.00	0.00	1.99
NE	WESTPORT	MA	0.98	1.71	2.29	2.97	2.57	3.22	0.00	0.00	4.66
NE	WEYMOUTH	MA	0.98	1.70	2.00	1.36	2.94	3.89	0.00	0.00	1.27
NE	WINTHROP	MA	0.99	2.24	2.24	2.02	2.12	2.24	0.00	0.00	1.30
NE	WOODS HOLE	MA	0.97	1.49	2.07	2.14	1.76	2.59	0.00	0.00	4.10
NE	YARMOUTH	MA	0.70	1.64	2.33	3.23	2.88	3.71	0.00	0.00	4.00
NE	ABINGDON	MD	1.00	1.64	2.72	1.56	3.00	4.00	0.00	0.00	1.23
NE	ACCOKEEK	MD	1.00	1.24	1.96	1.28	1.00	2.00	0.00	0.00	1.00
NE	BANKS O'DEE	MD	1.00	1.65	2.59	1.52	3.00	4.00	0.00	0.00	1.01
NE	BLAKE CREEK	MD	1.00	1.64	2.80	1.60	3.00	4.00	0.00	0.00	1.00
NE	BROAD CREEK	MD	1.00	1.64	2.80	1.60	3.00	4.00	0.00	0.00	1.00
NE	BUSHWOOD	MD	1.00	1.64	2.80	1.60	3.00	4.00	0.00	0.00	1.00
NE	CANOE NECK CREEK	MD	1.00	1.65	2.51	1.47	3.00	4.00	0.00	0.00	1.33
NE	CARTHEGENA CREEK	MD	1.00	1.66	2.45	1.45	3.00	4.00	0.00	0.00	1.05
NE	CHESAPEAKE BEACH	MD	1.00	1.68	1.96	1.24	3.00	4.00	0.00	0.00	1.00
NE	CHICAMUXEN CREEK	MD	1.00	1.64	2.80	1.60	3.00	4.00	0.00	0.00	1.00
NE	COBB ISLAND	MD	0.66	1.65	2.30	1.37	2.96	3.96	0.00	0.00	2.27
NE	COMBS CREEK	MD	1.00	1.66	2.42	1.44	3.00	4.00	0.00	0.00	1.13
NE	COOPER CREEK	MD	1.00	1.39	2.73	1.22	2.22	3.22	0.00	0.00	1.15
NE	CORNFIELD HARBOR	MD	0.89	1.53	2.33	1.62	1.64	2.64	0.00	0.00	2.65
NE	CRISFIELD	MD	1.00	1.64	2.73	1.57	3.00	4.00	0.00	0.00	1.18
NE	DISTRICT 1 ST. INIGOES	MD	1.00	1.64	2.80	1.60	3.00	4.00	0.00	0.00	1.00
NE	DISTRICT 6 HOOPERS ISLAND	MD	1.00	1.68	1.96	1.24	3.00	4.00	0.00	0.00	1.00
NE	DISTRICT 6 PATUXENT	MD	1.00	1.64	2.80	1.60	3.00	4.00	0.00	0.00	1.00
NE	DUKEHART CREEK	MD	1.00	1.64	2.80	1.60	3.00	4.00	0.00	0.00	1.00
NE	GOOSE BAY	MD	0.69	1.60	2.56	1.42	2.83	3.83	0.00	0.00	1.75
NE	ISLAND CREEK	MD	0.98	1.65	2.52	1.49	2.96	3.96	0.00	0.00	1.28
NE	LEONARDTOWN	MD	0.83	1.65	2.51	1.48	3.00	4.00	0.00	0.00	1.76
NE	MALLOWS BAY	MD	0.06	1.65	2.44	1.44	2.99	3.99	0.00	0.00	1.14
NE	MATTAWOMAN CREEK	MD	0.39	1.66	2.43	1.44	3.00	4.00	0.00	0.00	1.78
NE	MIDDLE RIVER	MD	1.00	1.64	2.80	1.60	3.00	4.00	0.00	0.00	1.01
NE	MORGANTOWN	MD	0.86	1.66	2.39	1.42	3.00	4.00	0.00	0.00	1.48
NE	MOUNT VICTORIA	MD	0.99	1.58	2.66	1.64	2.36	3.35	0.00	0.00	1.16
NE	NANJEMOY CREEK	MD	0.55	1.62	2.40	1.38	2.88	3.88	0.00	0.00	2.28
NE	NEWBURG	MD	0.93	1.60	2.79	1.54	2.88	3.88	0.00	0.00	1.45
NE	NORTH EAST	MD	1.00	1.68	1.96	1.24	3.00	4.00	0.00	0.00	1.00
NE	OCEAN CITY	MD	0.77	1.82	1.97	2.69	2.42	2.94	0.00	0.00	7.23
NE	PICCOWAXEN CREEK	MD	0.85	1.59	2.50	1.39	2.80	3.80	0.00	0.00	2.23
NE	PINEY POINT	MD	0.99	1.66	2.45	1.45	3.00	4.00	0.00	0.00	1.25
NE	POPES CREEK	MD	1.00	1.66	2.37	1.42	3.00	4.00	0.00	0.00	1.21
NE	POPLAR HILL CREEK	MD	1.00	1.64	2.80	1.60	3.00	4.00	0.00	0.00	1.00
NE	PORT TOBACCO VILLAGE	MD	0.97	1.51	2.76	1.41	2.60	3.60	0.00	0.00	1.09

NE	RIVERSIDE	MD	0.90	1.63	2.32	1.35	2.90	3.90	0.00	0.00	1.88
NE	SANDY POINT	MD	1.00	1.66	2.36	1.41	3.00	4.00	0.00	0.00	1.38
NE	SMITH CREEK	MD	0.99	1.64	2.46	1.47	2.86	3.86	0.00	0.00	1.24
NE	SMITH ISLAND	MD	1.00	1.64	2.80	1.60	3.00	4.00	0.00	0.00	1.00
NE	SMITH POINT	MD	0.61	1.64	2.30	1.34	2.93	3.93	0.00	0.00	2.60
NE	ST. CATHERINE SOUND	MD	1.00	1.64	2.80	1.60	3.00	4.00	0.00	0.00	1.00
NE	ST. CLEMENTS BAY	MD	1.00	1.67	2.17	1.33	3.00	4.00	0.00	0.00	1.01
NE	ST. GEORGES CREEK	MD	0.97	1.60	2.50	1.50	2.59	3.59	0.00	0.00	1.87
NE	ST. JEROMES	MD	1.00	1.63	2.45	1.49	2.79	3.79	0.00	0.00	1.42
NE	ST. PATRICK'S CREEK	MD	0.98	1.66	2.38	1.43	2.97	3.97	0.00	0.00	1.31
NE	TALL TIMBERS	MD	0.96	1.59	2.45	1.57	2.23	3.23	0.00	0.00	2.18
NE	WAVERLY CREEK	MD	1.00	1.64	2.80	1.60	3.00	4.00	0.00	0.00	1.00
NE	WHITE NECK CREEK	MD	1.00	1.64	2.41	1.40	2.93	3.93	0.00	0.00	1.39
NE	WICOMICO RIVER	MD	0.83	1.67	2.10	1.30	3.00	4.00	0.00	0.00	1.56
NE	ADDISON	ME	0.96	2.26	2.29	2.21	2.08	2.17	0.00	0.00	1.33
NE	ALNA	ME	1.00	2.12	2.24	1.39	2.67	3.67	0.00	0.00	1.00
NE	ARROWSIC	ME	1.00	1.82	2.28	3.48	3.00	4.00	0.00	0.00	1.00
NE	ARUNDEL	ME	1.00	1.32	2.72	1.12	2.00	3.00	0.00	0.00	1.00
NE	BAILEY ISLAND	ME	1.00	2.32	2.28	2.12	2.00	2.00	0.00	0.00	1.01
NE	BANGOR	ME	1.00	1.32	2.72	1.12	2.00	3.00	0.00	0.00	1.00
NE	BAR HARBOR	ME	1.00	2.15	2.26	2.39	2.18	2.37	0.00	0.00	1.52
NE	BATH	ME	0.99	1.46	2.48	2.67	3.07	3.80	0.00	0.00	2.20
NE	BEALS	ME	0.95	2.32	2.26	2.22	2.06	2.07	0.01	0.01	1.24
NE	BELFAST	ME	0.99	1.97	2.43	1.78	2.01	2.35	0.00	0.00	1.70
NE	BENTON FALLS	ME	1.00	2.52	2.00	1.52	3.00	4.00	0.00	0.00	1.00
NE	BIDDEFORD	ME	0.96	2.26	2.28	2.28	2.12	2.24	0.00	0.00	1.39
NE	BIRCH HARBOR	ME	0.90	2.16	2.79	1.86	2.29	2.39	0.00	0.00	1.74
NE	BLUE HILL	ME	0.99	2.17	2.31	2.22	2.12	2.23	0.00	0.00	1.57
NE	BOOTHBAY	ME	0.83	2.19	2.31	2.26	2.14	2.34	0.00	0.00	2.31
NE	BOOTHBAY HARBOR	ME	0.99	2.29	2.27	2.11	1.97	1.99	0.00	0.00	1.12
NE	BREMEN	ME	0.98	2.21	2.31	2.13	2.06	2.21	0.00	0.00	1.43
NE	BREWER	ME	1.00	1.32	2.72	1.12	2.00	3.00	0.00	0.00	1.00
NE	BRISTOL	ME	0.96	2.19	2.33	2.04	2.02	2.16	0.00	0.00	1.46
NE	BROOKLIN	ME	0.99	2.30	2.25	2.28	2.10	2.14	0.00	0.00	1.24
NE	BROOKSVILLE	ME	0.88	1.81	2.36	3.20	2.84	3.48	0.00	0.00	4.25
NE	BRUNSWICK	ME	1.00	1.71	2.32	3.56	3.39	3.96	0.00	0.00	2.47
NE	BUCKSPORT	ME	0.87	1.46	2.61	1.80	2.30	3.21	0.00	0.00	1.38
NE	BUNKERS HARBOR	ME	1.00	2.32	2.28	2.12	2.00	2.00	0.00	0.00	1.01
NE	CALAIS	ME	1.00	1.35	2.70	1.19	2.03	3.00	0.00	0.00	1.06
NE	CAMDEN	ME	1.00	1.49	2.62	1.92	2.35	2.97	0.00	0.00	1.92
NE	CAPE ELIZABETH	ME	1.00	2.32	2.28	2.15	2.02	2.03	0.00	0.00	1.04
NE	CASCO	ME	1.00	1.40	2.56	3.88	3.00	4.00	0.00	0.00	1.00
NE	CASTINE	ME	0.99	1.89	2.37	3.09	2.78	3.08	0.00	0.00	2.29
NE	CHAMBERLAIN	ME	1.00	1.32	2.72	1.12	2.00	3.00	0.00	0.00	1.00
NE	CHEBEAGUE ISLAND	ME	1.00	2.31	2.28	2.14	2.01	2.02	0.00	0.00	1.03
NE	CHERRYFIELD	ME	1.00	1.69	2.50	1.25	2.31	3.31	0.00	0.00	1.64
NE	COLUMBIA FALLS	ME	1.00	1.68	2.51	3.02	2.88	3.01	0.00	0.00	1.27
NE	COREA	ME	0.99	2.32	2.28	2.12	2.00	2.00	0.00	0.00	1.02
NE	CRANBERRY ISLES	ME	0.99	2.32	2.28	2.12	2.00	2.00	0.00	0.00	1.02
NE	CRIEHAVEN	ME	1.00	2.32	2.28	2.12	2.00	2.00	0.00	0.00	1.00

NE	CUMBERLAND CENTER	ME	1.00	2.02	2.31	2.55	2.58	2.72	0.00	0.00	1.42
NE	CUSHING	ME	0.99	2.30	2.28	2.18	2.04	2.09	0.00	0.01	1.11
NE	CUTLER	ME	0.99	2.26	2.23	2.51	2.25	2.38	0.00	0.00	1.73
NE	DAMARISCOTTA	ME	1.00	1.41	2.55	3.87	3.00	4.00	0.00	0.00	1.05
NE	DEER ISLE	ME	1.00	2.26	2.28	2.29	2.12	2.23	0.00	0.00	1.30
NE	DENNYSVILLE	ME	1.00	1.87	2.26	2.85	2.65	3.29	0.00	0.00	1.92
NE	DRESDEN	ME	1.00	2.52	2.00	1.52	3.00	4.00	0.00	0.00	1.00
NE	EAST CENTRAL WASHINGTON	ME	0.94	2.30	2.20	2.67	2.36	2.45	0.00	0.00	2.24
NE	EAST MACHIAS	ME	1.00	1.59	2.56	1.21	2.23	3.23	0.00	0.00	1.38
NE	EASTPORT	ME	0.99	2.31	2.24	2.31	2.11	2.12	0.00	0.00	1.29
NE	EDGECOMB	ME	0.65	1.70	2.48	2.97	2.56	3.25	0.00	0.00	2.49
NE	ELIOT	ME	0.99	1.77	1.94	2.68	2.37	2.59	0.00	0.00	2.64
NE	ELLSWORTH	ME	1.00	1.54	2.59	1.54	2.24	3.05	0.00	0.00	1.89
NE	FALMOUTH	ME	1.00	1.63	2.57	1.49	2.03	2.72	0.00	0.00	1.80
NE	FRANKFORT	ME	1.00	1.35	2.69	1.28	2.07	3.07	0.00	0.00	1.16
NE	FRANKLIN	ME	1.00	1.68	2.43	2.45	2.59	3.56	0.00	0.00	2.14
NE	FREEPORT	ME	0.99	2.03	2.30	2.82	2.49	2.98	0.00	0.00	2.35
NE	FRENCHBORO	ME	1.00	2.32	2.28	2.12	2.00	2.00	0.00	0.00	1.00
NE	FRIENDSHIP	ME	0.99	2.30	2.28	2.11	1.99	2.02	0.01	0.01	1.09
NE	GARDINER	ME	1.00	1.32	2.72	1.12	2.00	3.00	0.00	0.00	1.00
NE	GEORGETOWN	ME	0.99	2.25	2.29	2.27	2.09	2.19	0.00	0.00	1.24
NE	GOLDSBORO	ME	0.99	2.07	2.37	2.05	2.10	2.39	0.00	0.00	1.99
NE	GOULDSBORO	ME	1.00	2.31	2.29	2.12	2.00	2.01	0.00	0.00	1.03
NE	GREAT SPRUCE HEAD ISLAND	ME	0.99	2.31	2.27	2.15	2.01	2.02	0.00	0.01	1.09
NE	HAMPDEN	ME	0.98	1.48	2.58	1.89	2.38	3.29	0.00	0.00	1.43
NE	HANCOCK	ME	0.93	2.14	2.31	2.25	2.13	2.34	0.00	0.00	1.84
NE	HARPSWELL	ME	0.99	2.16	2.31	2.13	1.91	2.09	0.01	0.02	1.42
NE	HARPSWELL CENTER	ME	1.00	2.32	2.28	2.12	2.00	2.00	0.00	0.00	1.01
NE	HARRINGTON	ME	0.99	2.29	2.28	2.20	2.06	2.12	0.00	0.01	1.16
NE	ISLE AU HAUT	ME	1.00	2.30	1.86	3.22	2.52	2.52	0.00	0.00	1.36
NE	ISLESBORO	ME	1.00	2.14	2.25	2.42	2.20	2.40	0.00	0.00	1.49
NE	JEFFERSON	ME	1.00	2.52	2.00	1.52	3.00	4.00	0.00	0.00	1.00
NE	JONESBORO	ME	0.81	1.95	2.28	3.10	2.74	3.46	0.00	0.00	2.25
NE	JONESPORT	ME	0.94	2.25	2.18	2.44	2.28	2.39	0.01	0.01	1.88
NE	KENNEBUNK	ME	0.95	1.43	2.68	1.23	2.01	2.89	0.00	0.00	1.40
NE	KENNEBUNKPORT	ME	0.98	2.31	2.28	2.11	1.99	2.00	0.00	0.00	1.07
NE	KITTERY	ME	0.99	2.32	2.28	2.12	2.00	2.00	0.00	0.00	1.01
NE	KITTERY POINT	ME	1.00	2.44	1.80	4.00	3.00	3.00	0.00	0.00	1.00
NE	KNOX	ME	1.00	1.48	2.44	1.76	1.00	2.00	0.00	0.00	1.00
NE	LAMOINE	ME	1.00	2.01	2.22	2.67	2.38	2.77	0.00	0.00	1.83
NE	LINCOLNVILLE	ME	0.91	2.10	2.37	1.94	2.02	2.25	0.00	0.00	1.92
NE	LUBEC	ME	0.98	2.10	2.25	2.95	2.59	2.78	0.00	0.00	3.37
NE	MACHIAS	ME	1.00	1.54	2.56	1.91	2.32	3.20	0.00	0.00	2.12
NE	MACHIASPORT	ME	0.98	2.25	2.26	2.41	2.20	2.35	0.00	0.00	1.59
NE	MATINICUS ISLE	ME	1.00	2.32	2.28	2.12	2.00	2.00	0.00	0.00	1.00
NE	MILBRIDGE	ME	0.98	2.30	2.28	2.19	2.05	2.09	0.00	0.01	1.15
NE	MONHEGAN	ME	0.94	2.32	2.30	2.11	2.01	2.01	0.00	0.00	1.16
NE	MOUNT DESERT	ME	1.00	2.24	2.29	2.16	2.06	2.14	0.00	0.00	1.29
NE	NEW HARBOR	ME	0.99	2.28	2.29	2.09	1.98	2.02	0.00	0.00	1.12
NE	NEWCASTLE	ME	1.00	1.90	2.34	2.13	2.75	3.75	0.00	0.00	2.33
NE	NOBLEBORO	ME	1.00	2.32	2.28	2.12	2.00	2.00	0.00	0.00	1.00
NE	NORTH HAVEN	ME	1.00	2.31	2.28	2.14	2.01	2.02	0.00	0.00	1.03
NE	NORTHPORT	ME	1.00	1.38	2.30	2.73	2.65	3.64	0.00	0.00	1.05
NE	OGUNQUIT	ME	0.97	2.31	2.28	2.12	2.00	2.01	0.00	0.00	1.09

NE	ORLAND	ME	0.96	1.51	2.60	1.28	2.18	3.18	0.00	0.00	1.57
NE	ORRINGTON	ME	0.92	1.32	2.72	1.12	2.00	3.00	0.00	0.00	1.12
NE	OWLS HEAD	ME	1.00	2.32	2.28	2.12	2.00	2.01	0.00	0.01	1.02
NE	PASSAMAQUODDY PLEASANT POINT	ME	1.00	1.57	2.50	2.30	2.50	3.50	0.00	0.00	1.00
NE	PEMAQUID	ME	1.00	1.63	2.55	2.10	2.25	2.97	0.00	0.00	2.59
NE	PEMBROKE	ME	0.96	2.17	2.05	3.65	2.98	3.31	0.00	0.00	2.42
NE	PENOBSCOT	ME	0.98	1.56	2.53	1.76	2.25	3.07	0.00	0.00	1.74
NE	PERRY	ME	0.88	2.17	2.14	3.01	2.81	3.34	0.00	0.00	3.70
NE	PHIPPSBURG	ME	0.99	2.25	2.29	2.23	2.09	2.19	0.00	0.00	1.31
NE	PORT CLYDE	ME	0.98	2.30	2.30	2.10	1.98	1.99	0.00	0.00	1.13
NE	PORTLAND	ME	0.97	2.13	2.18	2.02	1.88	1.99	0.02	0.01	2.58
NE	PROSPECT	ME	1.00	1.32	2.71	1.13	1.98	2.98	0.00	0.00	1.04
NE	PROSPECT HARBOR	ME	1.00	2.27	2.27	2.07	1.97	2.01	0.00	0.00	1.16
NE	PULPIT HARBOR	ME	1.00	2.32	2.28	2.12	2.00	2.00	0.00	0.00	1.00
NE	ROBBINSTON	ME	1.00	2.21	2.00	3.73	2.95	3.32	0.00	0.00	1.22
NE	ROCKLAND	ME	0.99	2.21	2.12	2.09	1.88	1.88	0.01	0.00	1.71
NE	ROCKPORT	ME	0.98	2.23	2.32	2.04	2.01	2.09	0.00	0.00	1.26
NE	ROQUE BLUFFS	ME	0.97	1.97	2.22	3.37	2.87	3.59	0.00	0.00	1.87
NE	SACO	ME	0.64	2.15	2.36	1.95	2.00	2.17	0.00	0.00	2.34
NE	SAINT GEORGE	ME	0.94	2.20	2.29	2.37	2.19	2.39	0.00	0.00	1.70
NE	SCARBOROUGH	ME	0.99	2.15	2.29	2.52	2.29	2.60	0.00	0.00	1.85
NE	SEARSMONT	ME	1.00	1.32	2.72	1.12	2.00	3.00	0.00	0.00	1.00
NE	SEARSPORT	ME	0.75	1.88	2.41	1.99	2.09	2.54	0.00	0.00	2.43
NE	SEDGWICK	ME	0.97	1.66	2.55	1.59	2.08	2.77	0.00	0.00	1.56
NE	SORRENTO	ME	1.00	2.30	2.28	2.15	2.01	2.03	0.00	0.00	1.09
NE	SOUTH BRISTOL	ME	0.99	2.11	2.33	2.49	2.20	2.44	0.00	0.00	1.72
NE	SOUTH FREPORT	ME	0.94	2.19	1.98	3.97	3.00	3.24	0.00	0.00	1.16
NE	SOUTH PORTLAND	ME	0.75	1.56	2.29	1.33	1.73	2.38	0.00	0.00	1.70
NE	SOUTHPORT	ME	1.00	2.32	2.28	2.12	2.00	2.00	0.00	0.00	1.01
NE	SOUTHWEST HARBOR	ME	1.00	2.32	2.28	2.14	2.01	2.01	0.00	0.01	1.03
NE	STEUBEN	ME	0.99	2.26	2.30	2.10	2.02	2.09	0.00	0.00	1.18
NE	STOCKTON SPRINGS	ME	0.96	2.31	2.28	2.13	2.01	2.02	0.00	0.00	1.10
NE	STONINGTON	ME	1.00	2.31	2.27	2.17	2.03	2.04	0.01	0.02	1.09
NE	SULLIVAN	ME	0.94	1.86	2.42	2.16	2.28	2.86	0.00	0.00	3.02
NE	SURRY	ME	1.00	1.94	2.44	1.79	2.03	2.41	0.00	0.00	1.82
NE	SWANS ISLAND	ME	1.00	2.31	2.28	2.14	2.01	2.03	0.00	0.00	1.03
NE	TENANTS HARBOR	ME	0.98	2.31	2.28	2.14	2.01	2.02	0.00	0.00	1.09
NE	THOMASTON	ME	0.94	2.03	2.33	2.66	2.38	2.76	0.00	0.00	2.71
NE	TOPSHAM	ME	1.00	1.76	2.41	2.62	2.76	3.24	0.00	0.00	1.66
NE	TREMONT	ME	1.00	2.32	2.28	2.14	2.01	2.02	0.00	0.01	1.04
NE	TRENTON	ME	0.98	2.21	2.30	2.34	2.15	2.28	0.00	0.00	1.43
NE	UNION	ME	1.00	1.32	2.72	1.12	2.00	3.00	0.00	0.00	1.00
NE	VASSALBORO	ME	1.00	2.52	2.00	1.52	3.00	4.00	0.00	0.00	1.00
NE	VERONA	ME	1.00	2.32	2.28	2.12	2.00	2.00	0.00	0.00	1.00
NE	VINALHAVEN	ME	1.00	2.32	2.28	2.13	2.00	2.01	0.01	0.02	1.02
NE	WALDO	ME	1.00	1.49	2.57	1.90	2.33	3.33	0.00	0.00	1.01
NE	WALDOBORO	ME	0.99	1.56	2.52	2.19	2.45	3.42	0.00	0.00	2.02
NE	WARREN	ME	1.00	1.59	2.56	1.21	2.23	3.23	0.00	0.00	1.48
NE	WELLS	ME	0.82	2.27	2.30	2.07	2.00	2.05	0.00	0.00	1.56
NE	WEST BATH	ME	1.00	1.66	2.34	3.72	3.55	3.98	0.00	0.00	2.45
NE	WEST JONESPORT	ME	1.00	2.02	2.38	2.70	2.50	2.50	0.00	0.00	1.00
NE	WESTPORT ISLAND	ME	0.88	2.29	2.28	2.17	2.04	2.08	0.00	0.00	1.40

NE	WHITING	ME	1.00	1.78	2.39	3.13	2.87	3.16	0.00	0.00	2.26
NE	WHITNEYVILLE	ME	0.84	1.72	2.48	3.28	3.00	3.00	0.00	0.00	1.37
NE	WINTER HARBOR	ME	1.00	2.28	2.29	2.14	2.04	2.06	0.00	0.00	1.14
NE	WISCASSET	ME	0.43	2.19	2.28	2.46	2.26	2.51	0.00	0.00	2.25
NE	WOOLWICH	ME	0.89	2.13	2.16	2.33	2.89	3.89	0.00	0.00	2.38
NE	YARMOUTH	ME	0.98	1.59	2.50	2.38	2.48	3.41	0.00	0.00	2.60
NE	YORK HARBOR	ME	0.97	2.32	2.28	2.12	2.00	2.00	0.00	0.00	1.07
NE	DOVER	NH	1.00	2.28	2.29	2.19	2.04	2.08	0.00	0.00	1.08
NE	DURHAM	NH	1.00	1.44	2.55	3.81	2.96	3.92	0.00	0.00	1.09
NE	GREENLAND	NH	1.00	1.40	2.56	3.88	3.00	4.00	0.00	0.00	1.00
NE	HAMPTON	NH	0.66	2.15	2.32	2.53	2.23	2.43	0.00	0.00	2.74
NE	HAMPTON/SEABROOK	NH	0.18	1.10	1.02	0.56	1.00	1.00	0.00	0.00	1.20
NE	NEW CASTLE	NH	0.47	2.42	1.87	3.75	2.86	2.87	0.00	0.00	2.18
NE	NEWINGTON	NH	1.00	2.31	2.28	2.14	2.01	2.02	0.00	0.01	1.03
NE	PORTSMOUTH	NH	0.98	2.19	2.29	2.02	1.84	1.96	0.00	0.00	1.61
NE	RYE	NH	0.88	2.22	2.23	1.98	1.90	1.93	0.00	0.00	2.97
NE	SEABROOK	NH	0.91	2.24	2.31	1.98	1.87	1.90	0.00	0.00	1.94
NE	ABSECON	NJ	1.00	2.04	1.96	1.08	1.00	1.00	0.00	0.00	1.00
NE	ATLANTIC CITY	NJ	1.00	2.05	1.80	3.73	3.24	3.24	0.02	0.01	1.64
NE	BARNEGAT	NJ	0.69	1.80	2.01	2.37	1.91	2.43	0.00	0.00	3.20
NE	BARNEGAT LIGHT	NJ	0.93	2.36	1.84	3.63	2.84	2.87	0.00	0.01	1.54
NE	BELFORD	NJ	1.00	1.40	2.00	1.41	1.09	1.85	0.00	0.00	3.49
NE	BELMAR	NJ	0.98	2.32	2.27	2.12	2.00	2.00	0.00	0.00	1.06
NE	BRICK	NJ	1.00	1.24	1.96	1.28	1.00	2.00	0.00	0.00	1.00
NE	BRIELLE	NJ	0.90	1.70	1.70	2.02	1.72	2.25	0.00	0.00	1.24
NE	BRIGANTINE	NJ	1.00	0.71	0.91	1.51	1.33	1.60	0.00	0.00	1.41
NE	CAPE MAY	NJ	1.00	2.02	1.97	3.04	2.20	2.43	0.08	0.03	2.49
NE	ELIZABETH	NJ	1.00	1.48	2.44	1.76	1.00	2.00	0.00	0.00	1.00
NE	FORKED RIVER	NJ	1.00	1.24	1.96	1.28	1.00	2.00	0.00	0.00	1.00
NE	FORTESCUE	NJ	1.00	1.58	2.66	1.66	2.23	3.23	0.00	0.00	1.33
NE	HIGHLANDS	NJ	0.75	1.24	1.96	1.28	1.00	2.00	0.00	0.00	1.24
NE	KEYPORT	NJ	1.00	1.48	2.44	1.76	1.00	2.00	0.00	0.00	1.00
NE	MANASQUAN	NJ	0.83	1.40	1.91	1.48	1.02	1.82	0.00	0.00	1.47
NE	MANTOLOKING	NJ	1.00	1.32	2.72	1.12	2.00	3.00	0.00	0.00	1.00
NE	MAURICE RIVER	NJ	1.00	0.75	1.21	0.86	0.69	1.19	0.00	0.00	1.86
NE	NEPTUNE	NJ	1.00	1.86	1.82	1.70	1.60	1.60	0.00	0.00	1.00
NE	OCEAN CITY	NJ	1.00	1.64	2.80	1.60	3.00	4.00	0.00	0.00	1.20
NE	PINE BEACH	NJ	1.00	1.63	1.90	1.46	1.12	1.24	0.00	0.00	4.40
NE	POINT PLEASANT BEACH	NJ	0.97	2.10	1.71	3.07	2.63	2.91	0.02	0.01	4.73
NE	PORT NORRIS	NJ	0.94	1.40	2.57	1.44	1.50	2.50	0.00	0.00	1.20
NE	PORT REPUBLIC	NJ	0.81	1.51	2.51	1.71	2.08	3.08	0.00	0.00	1.50
NE	RED BANK	NJ	1.00	1.48	2.44	1.76	1.00	2.00	0.00	0.00	1.00
NE	RUMSON	NJ	1.00	1.42	2.55	1.50	1.40	2.40	0.00	0.00	1.23
NE	SEA BRIGHT	NJ	0.99	1.17	1.24	1.12	0.79	1.50	0.00	0.00	1.41
NE	SEA ISLE CITY	NJ	0.42	1.88	2.15	2.01	1.97	2.43	0.00	0.00	6.04
NE	TUCKERTON	NJ	1.00	1.64	2.80	1.60	3.00	4.00	0.00	0.00	1.00
NE	WARETOWN	NJ	0.67	1.88	2.29	1.36	1.81	2.21	0.00	0.00	1.51
NE	WILDWOOD	NJ	0.99	2.28	1.86	3.89	3.00	3.00	0.00	0.00	1.48
NE	AMAGANSETT	NY	0.98	1.50	1.67	1.34	1.45	2.20	0.00	0.00	5.69
NE	AMITY HARBOR	NY	1.00	1.40	2.04	1.84	2.00	3.00	0.00	0.00	1.00
NE	ATLANTIC BEACH	NY	1.00	1.45	1.96	1.26	1.97	2.96	0.00	0.00	1.03
NE	BAY SHORE	NY	0.94	1.65	2.76	1.62	2.97	3.96	0.00	0.00	1.25
NE	BELLPORT	NY	0.89	1.64	2.70	1.59	2.57	3.52	0.00	0.00	1.47
NE	BILTMORE SHORES	NY	1.00	2.33	2.12	2.23	2.97	3.97	0.00	0.00	1.07
NE	BRONX	NY	1.00	2.13	2.20	2.12	2.49	3.49	0.00	0.00	1.01

NE	BROOKLYN	NY	0.95	2.09	2.24	2.29	2.70	3.56	0.00	0.00	3.39
NE	CAPTREE	NY	0.99	1.75	2.46	1.61	2.91	3.89	0.00	0.00	1.85
NE	CATSKILL	NY	0.49	2.16	2.11	1.51	2.60	3.39	0.00	0.00	1.39
NE	CENTER MORICHES	NY	0.93	1.49	2.13	1.45	1.82	2.74	0.00	0.00	5.38
NE	CENTERPORT	NY	0.93	1.97	2.02	1.63	1.96	2.64	0.00	0.00	2.05
NE	CITY ISLAND	NY	1.00	2.29	2.10	2.21	2.96	3.96	0.00	0.00	1.51
NE	COLD SPRING	NY	1.00	2.22	2.06	2.18	3.00	4.00	0.00	0.00	1.00
NE	CORNWALL-ON-HUDSON	NY	1.00	2.52	2.00	1.52	3.00	4.00	0.00	0.00	1.00
NE	COXSACKIE	NY	1.00	1.80	2.72	1.48	3.00	4.00	0.00	0.00	1.00
NE	CROTON-ON-HUDSON	NY	0.73	1.81	2.34	1.66	1.79	2.79	0.00	0.00	1.75
NE	EAST HAMPTON	NY	0.91	1.68	2.10	1.72	2.29	3.16	0.00	0.00	4.91
NE	EAST MARION	NY	1.00	1.67	2.09	1.67	1.91	2.41	0.00	0.00	1.18
NE	EAST MORICHES	NY	0.84	1.54	2.18	1.40	2.16	2.97	0.00	0.00	4.27
NE	EAST QUOGUE	NY	1.00	2.08	2.00	2.12	3.00	4.00	0.00	0.00	1.00
NE	EASTPORT	NY	0.88	1.71	2.03	1.40	2.27	3.05	0.00	0.00	2.70
NE	ESOPUS	NY	1.00	2.52	2.00	1.52	3.00	4.00	0.00	0.00	1.00
NE	FISHERS ISLAND	NY	0.98	1.48	1.96	1.27	2.00	2.97	0.00	0.00	1.10
NE	FLANDERS	NY	0.20	2.08	2.00	2.12	3.00	4.00	0.00	0.00	1.89
NE	FREEPORT	NY	0.94	1.84	2.14	1.88	2.21	2.81	0.00	0.00	6.97
NE	GERRITSEN	NY	0.97	2.33	2.12	2.22	2.97	3.96	0.00	0.00	1.13
NE	GLEN COVE	NY	1.00	2.14	2.09	2.14	2.83	3.83	0.00	0.00	1.43
NE	GREAT NECK	NY	1.00	1.48	2.44	1.76	1.00	2.00	0.00	0.00	1.00
NE	GREENPORT	NY	0.97	1.58	1.90	2.09	1.95	2.79	0.00	0.00	6.21
NE	HAMILTON BEACH	NY	1.00	1.72	1.40	1.59	1.57	2.56	0.00	0.00	2.06
NE	HAMPTON BAYS/SHINNECOCK	NY	0.98	1.69	1.91	1.61	1.78	2.14	0.00	0.00	6.49
NE	HANCOCK	NY	1.00	1.32	2.72	1.12	2.00	3.00	0.00	0.00	1.00
NE	HARBOR GREEN	NY	1.00	2.36	2.12	2.24	3.00	4.00	0.00	0.00	1.00
NE	HAVERSTRAW	NY	0.90	1.56	2.51	1.69	1.50	2.50	0.00	0.00	1.25
NE	HEMPSTEAD	NY	1.00	1.68	2.59	1.63	1.33	2.19	0.00	0.00	1.70
NE	HEWLETT HARBOR	NY	1.00	1.68	1.96	1.24	3.00	4.00	0.00	0.00	1.00
NE	HUNTINGTON	NY	0.93	1.95	1.77	1.80	1.83	2.43	0.00	0.00	3.19
NE	ISLAND PARK	NY	0.99	1.75	2.04	1.68	2.24	3.21	0.00	0.00	3.56
NE	ISLIP	NY	0.52	1.73	2.14	1.86	2.52	3.26	0.00	0.00	3.54
NE	JAMAICA BAY-ROCKAWAY	NY	0.95	2.24	2.11	2.14	2.77	3.77	0.00	0.00	1.44
NE	JAMESPORT	NY	0.96	1.54	2.18	1.83	1.84	2.69	0.00	0.00	3.33
NE	JONES POINT	NY	1.00	1.68	1.96	1.24	3.00	4.00	0.00	0.00	1.00
NE	KINGS PARK	NY	1.00	2.08	2.00	2.12	3.00	4.00	0.00	0.00	1.00
NE	KINGSTON	NY	0.53	1.77	2.42	1.29	2.34	3.30	0.00	0.00	1.85
NE	LONG BEACH	NY	1.00	1.66	1.96	1.40	2.50	3.48	0.00	0.00	1.72
NE	MAMARONECK	NY	1.00	2.36	2.12	2.24	3.00	4.00	0.00	0.00	1.00
NE	MASTIC	NY	0.94	1.69	2.03	1.76	2.38	3.35	0.00	0.00	1.75
NE	MATTITUCK	NY	0.96	1.55	1.57	1.58	1.50	2.42	0.00	0.00	4.03
NE	MONTAUK	NY	0.97	1.70	1.86	1.48	1.67	2.05	0.01	0.01	7.75
NE	MONTROSE	NY	0.52	1.48	2.44	1.76	1.00	2.00	0.00	0.00	1.05
NE	MORICHES	NY	0.95	1.71	2.35	1.62	2.52	3.49	0.00	0.00	4.52
NE	MOUNT SINAI	NY	0.88	1.82	1.89	1.85	2.06	3.03	0.00	0.00	4.40
NE	NAPEAGUE	NY	1.00	1.68	1.96	1.24	3.00	4.00	0.00	0.00	1.00
NE	NARROWSBURG	NY	1.00	1.32	2.72	1.12	2.00	3.00	0.00	0.00	1.00
NE	NEW ROCHELLE	NY	1.00	1.83	2.37	2.45	2.56	3.45	0.00	0.00	2.68
NE	NEW SUFFOLK	NY	1.00	1.76	2.14	2.81	2.97	3.97	0.00	0.00	1.12
NE	NEW YORK	NY	0.97	1.96	2.22	2.19	2.34	3.20	0.00	0.00	2.69

NE	NISSEQUOGUE	NY	1.00	1.46	2.23	1.40	1.76	2.69	0.00	0.00	2.13
NE	NORTHPORT	NY	0.96	2.06	2.08	2.10	2.70	3.65	0.00	0.00	2.01
NE	NOYACK	NY	1.00	1.56	1.04	1.32	1.00	2.00	0.00	0.00	1.00
NE	OAK BEACH	NY	1.00	1.81	2.00	1.53	2.89	3.89	0.00	0.00	1.32
NE	OAKDALE	NY	1.00	1.78	2.01	1.29	3.00	3.77	0.00	0.00	1.06
NE	OCEANSIDE	NY	1.00	1.96	2.05	2.92	2.72	3.08	0.00	0.00	1.91
NE	ORIENT	NY	0.99	1.98	2.13	1.78	2.33	2.80	0.00	0.00	2.42
NE	OSSINING	NY	0.81	1.53	2.32	1.73	1.00	1.89	0.00	0.00	1.44
NE	OYSTER BAY	NY	0.99	1.94	2.29	1.89	2.51	3.41	0.00	0.00	2.46
NE	PATCHOGUE	NY	0.92	1.74	2.14	1.64	2.02	2.85	0.00	0.00	4.45
NE	POINT LOOKOUT	NY	0.99	1.74	1.98	1.95	2.18	3.06	0.00	0.00	3.86
NE	PORT CHESTER	NY	1.00	2.36	2.12	2.24	2.99	3.99	0.00	0.00	1.01
NE	PORT JEFFERSON	NY	1.00	1.70	1.42	1.53	1.46	2.44	0.00	0.00	1.61
NE	PORT WASHINGTON	NY	1.00	2.21	2.12	2.17	2.79	3.79	0.00	0.00	1.38
NE	QUEENS	NY	1.00	2.36	2.12	2.24	3.00	4.00	0.00	0.00	1.00
NE	RIVERHEAD	NY	0.99	1.54	2.35	1.72	1.11	2.02	0.00	0.00	1.76
NE	ROCKAWAY PARK	NY	0.97	2.14	2.07	2.07	2.64	3.63	0.00	0.00	1.70
NE	ROCKAWAY POINT	NY	1.00	1.37	1.99	1.48	1.72	2.63	0.00	0.00	1.70
NE	SAG HARBOR	NY	0.99	1.68	2.07	1.31	2.92	3.91	0.00	0.00	1.57
NE	SAUGERTIES	NY	0.54	1.80	2.72	1.48	3.00	4.00	0.00	0.00	1.06
NE	SEAFORD	NY	0.98	1.70	2.41	1.74	2.48	3.46	0.00	0.00	2.62
NE	SETAUKET-EAST SETAUKET	NY	1.00	2.21	2.09	2.17	2.82	3.82	0.00	0.00	1.38
NE	SHELTER ISLAND	NY	0.99	1.74	2.23	3.21	2.74	3.73	0.00	0.00	1.91
NE	SHOREHAM	NY	1.00	2.08	2.01	2.03	2.60	3.56	0.00	0.00	1.98
NE	SMITHTOWN	NY	1.00	1.96	1.87	1.89	2.64	3.63	0.00	0.00	1.58
NE	SOUTHAMPTON	NY	0.97	1.64	2.05	1.46	2.39	3.35	0.00	0.00	1.88
NE	SOUTHOLD	NY	0.79	1.90	2.06	1.87	2.65	3.58	0.00	0.00	3.62
NE	SPRINGS	NY	1.00	1.69	1.97	1.34	2.92	3.92	0.00	0.00	1.41
NE	STATEN ISLAND	NY	0.78	1.66	2.46	1.95	1.56	2.23	0.00	0.00	1.54
NE	STONY BROOK	NY	0.95	1.63	2.00	1.78	2.08	3.04	0.00	0.00	2.05
NE	STUYVESANT	NY	0.89	2.45	2.07	1.52	3.00	4.00	0.00	0.00	1.51
NE	TIVOLI	NY	1.00	2.52	2.00	1.52	3.00	4.00	0.00	0.00	1.00
NE	ULSTER PARK	NY	1.00	1.96	1.36	1.52	1.00	1.00	0.00	0.00	1.00
NE	VAN KEURENS	NY	0.90	1.64	2.80	1.60	2.99	3.99	0.00	0.00	1.27
NE	WAINSCOTT	NY	0.99	1.46	1.71	1.33	1.37	2.17	0.00	0.00	4.05
NE	WEST HAMPTON DUNES	NY	1.00	2.08	2.00	2.12	3.00	4.00	0.00	0.00	1.00
NE	WESTHAMPTON	NY	1.00	1.89	2.18	1.88	2.74	3.74	0.00	0.00	1.03
NE	WHITESTONE	NY	1.00	2.35	2.13	2.23	2.99	3.99	0.00	0.00	1.03
NE	YONKERS	NY	1.00	1.48	2.44	1.76	1.00	2.00	0.00	0.00	1.00
NE	BARRINGTON	RI	1.00	1.68	2.28	3.78	3.98	4.00	0.00	0.00	1.03
NE	BRISTOL	RI	0.95	1.73	2.25	3.60	3.26	3.86	0.00	0.00	3.02
NE	CHARLESTOWN	RI	1.00	1.45	2.55	3.11	2.83	3.71	0.00	0.00	1.51
NE	EAST GREENWICH	RI	1.00	1.68	2.28	3.80	4.00	4.00	0.00	0.00	1.01
NE	JAMESTOWN	RI	1.00	1.47	2.29	2.94	2.53	3.46	0.00	0.00	2.87
NE	LITTLE COMPTON	RI	0.86	1.95	1.99	1.58	1.49	1.75	0.00	0.00	6.17
NE	MELVILLE	RI	0.78	2.06	2.16	2.67	3.24	4.00	0.00	0.00	2.58
NE	MIDDLETOWN	RI	1.00	1.48	2.02	1.77	2.11	2.99	0.00	0.00	3.01
NE	NARRAGANSETT/POINT JUDITH	RI	0.96	1.85	1.89	2.29	1.78	1.93	0.04	0.03	5.13
NE	NEW SHOREHAM	RI	0.88	2.03	2.31	2.40	2.24	2.54	0.00	0.00	2.63
NE	NEWPORT	RI	0.75	2.21	2.22	2.00	1.83	1.91	0.00	0.00	2.44
NE	NORTH KINGSTOWN	RI	1.00	1.49	1.96	1.98	1.35	1.43	0.02	0.01	2.98
NE	PORTSMOUTH	RI	0.90	1.67	2.09	3.20	2.51	3.50	0.00	0.00	2.27

NE	PROVIDENCE	RI	1.00	1.65	2.10	2.81	2.63	3.61	0.00	0.00	1.52
NE	SOUTH KINGSTOWN	RI	0.99	1.50	2.19	2.28	2.14	3.03	0.00	0.00	3.19
NE	TIVERTON	RI	0.41	1.70	1.89	1.90	1.75	2.45	0.00	0.00	5.28
NE	WARREN	RI	0.82	1.80	2.27	3.77	2.93	3.90	0.00	0.00	1.54
NE	WARWICK	RI	0.99	1.69	2.24	3.64	3.84	3.95	0.00	0.00	1.27
NE	WESTERLY	RI	0.98	1.47	2.25	2.60	2.40	3.34	0.00	0.00	2.46
NE	ACCOMAC	VA	0.97	1.62	2.49	2.72	3.27	3.91	0.00	0.01	3.48
NE	AQUIA CREEK	VA	0.21	1.65	2.59	1.51	3.00	4.00	0.00	0.00	1.43
NE	ATLANTIC	VA	1.00	1.41	2.55	3.88	3.00	4.00	0.00	0.00	1.09
NE	BATTERY PARK	VA	1.00	1.40	2.56	3.87	3.00	4.00	0.00	0.00	1.01
NE	BELLE HAVEN	VA	1.00	1.53	2.58	3.05	3.14	3.96	0.00	0.00	1.48
NE	BELVEDERE BEACH	VA	1.00	1.64	2.80	1.60	3.00	4.00	0.00	0.00	1.00
NE	CAPE CHARLES	VA	0.98	1.69	2.41	3.05	3.52	3.86	0.00	0.00	2.08
NE	CENTER CROSS	VA	1.00	1.40	2.56	3.88	3.00	4.00	0.00	0.00	1.00
NE	CHERITON	VA	1.00	1.65	2.31	3.81	3.88	4.00	0.00	0.00	1.27
NE	CHERRY HILL	VA	0.30	1.57	2.55	1.38	2.75	3.75	0.00	0.00	1.26
NE	CHINCOTEAGUE	VA	0.98	1.53	2.12	2.40	2.37	2.95	0.00	0.00	6.40
NE	COAN RIVER	VA	1.00	1.66	2.43	1.44	3.00	4.00	0.00	0.00	1.10
NE	COBBS CREEK	VA	0.90	1.46	2.29	2.14	1.88	2.88	0.00	0.00	1.86
NE	COLES POINT	VA	1.00	1.53	2.66	2.65	3.00	4.00	0.00	0.00	1.07
NE	COPLE	VA	0.99	1.66	2.35	1.40	2.99	3.99	0.00	0.00	1.57
NE	DAHLGREN	VA	0.78	1.66	2.31	1.39	3.00	4.00	0.00	0.00	1.63
NE	DAUGHERTY	VA	0.99	1.34	1.95	1.43	1.00	2.00	0.00	0.00	1.29
NE	DELTAVILLE	VA	0.99	1.45	2.58	3.41	2.99	3.99	0.00	0.00	1.50
NE	DISTRICT 1 NORTHUMBERLA ND	VA	1.00	1.68	2.29	3.79	3.98	4.00	0.00	0.00	1.04
NE	DISTRICT 3 NEW KENT	VA	0.89	1.64	2.80	1.60	3.00	4.00	0.00	0.00	1.23
NE	DISTRICT 3 NORTHAMPTON	VA	1.00	1.68	2.31	3.67	3.94	4.00	0.00	0.00	1.15
NE	DISTRICT 4 NORTHAMPTON	VA	0.96	1.52	2.48	2.76	2.85	3.51	0.00	0.00	2.70
NE	DISTRICT 5 NORTHAMPTON	VA	1.00	1.57	2.55	3.09	3.33	4.00	0.00	0.00	1.00
NE	DISTRICT 6 ACCOMACK	VA	0.99	1.55	2.69	2.44	2.95	3.95	0.00	0.00	1.40
NE	EASTVILLE	VA	1.00	1.65	2.60	2.46	3.36	4.00	0.00	0.00	1.98
NE	FAIRVIEW BEACH	VA	0.38	1.65	2.31	1.37	2.96	3.96	0.00	0.00	1.97
NE	GLOUCESTER POINT	VA	0.80	1.56	2.66	2.54	3.03	3.96	0.00	0.00	1.79
NE	GREENBACKVILLE	VA	0.99	1.64	2.80	1.60	3.00	4.00	0.00	0.00	1.01
NE	GUNSTON COVE	VA	1.00	1.64	2.80	1.60	3.00	4.00	0.00	0.00	1.00
NE	GWYNN	VA	1.00	1.55	2.59	2.01	2.66	3.66	0.00	0.00	1.63
NE	HACK CREEK	VA	1.00	1.68	1.96	1.24	3.00	4.00	0.00	0.00	1.00
NE	HAGUE	VA	1.00	1.52	2.68	2.74	3.00	4.00	0.00	0.00	1.00
NE	HAMPTON	VA	0.92	1.87	1.96	2.63	2.24	2.70	0.00	0.01	3.91
NE	HAMPTON HALL BRANCH	VA	1.00	1.64	2.80	1.60	3.00	4.00	0.00	0.00	1.00
NE	HARBORTON	VA	0.99	1.59	2.71	1.95	2.88	3.87	0.00	0.00	1.44
NE	HARRYHOGAN	VA	0.96	1.59	2.07	1.30	2.60	3.60	0.00	0.00	1.33
NE	HARTFIELD	VA	0.87	1.66	2.38	1.42	3.00	4.00	0.00	0.00	1.29
NE	HUDGINS	VA	0.99	1.47	2.38	3.05	2.83	3.82	0.00	0.00	1.30
NE	HULL CREEK	VA	1.00	1.64	2.80	1.60	3.00	4.00	0.00	0.00	1.00
NE	KILLNECK CREEK	VA	1.00	1.64	2.80	1.60	3.00	4.00	0.00	0.00	1.00
NE	KILMARNOCK	VA	1.00	1.54	2.69	2.57	2.98	3.98	0.00	0.00	1.44

NE	KING WILLIAM	VA	0.82	1.64	2.76	1.59	3.00	4.00	0.00	0.00	1.55
NE	KINGSCOTE CREEK	VA	1.00	1.67	2.09	1.29	3.00	4.00	0.00	0.00	1.35
NE	KIPTOPEKE	VA	0.76	1.56	2.30	2.70	3.03	3.49	0.00	0.00	2.03
NE	LEWISETTA	VA	0.98	1.56	2.71	2.37	2.92	3.92	0.00	0.00	1.15
NE	LITTLE WICOMICO RIVER	VA	0.92	1.53	2.35	1.57	1.81	2.77	0.00	0.00	2.74
NE	LOCUST HILL	VA	1.00	1.58	2.74	2.17	3.00	4.00	0.00	0.00	1.00
NE	LODGE CREEK	VA	1.00	1.68	1.96	1.24	3.00	4.00	0.00	0.00	1.00
NE	MOBJACK	VA	1.00	1.56	2.60	2.33	2.90	3.90	0.00	0.00	1.87
NE	MOLLUSK	VA	1.00	1.40	2.56	3.88	3.00	4.00	0.00	0.00	1.00
NE	MONTROSS	VA	0.98	1.66	2.36	1.41	3.00	4.00	0.00	0.00	1.25
NE	MORATTICO	VA	1.00	1.56	2.72	2.35	3.00	4.00	0.00	0.00	1.28
NE	MOUNT HOLLY	VA	1.00	1.56	2.72	2.36	3.00	4.00	0.00	0.00	1.00
NE	MOUNT VERNON	VA	1.00	1.68	1.96	1.24	3.00	4.00	0.00	0.00	1.00
NE	MUNDY POINT	VA	1.00	1.66	2.39	1.42	3.00	4.00	0.00	0.00	1.20
NE	NANSEMOND	VA	0.75	1.72	2.41	3.33	3.00	4.00	0.00	0.00	1.21
NE	NEWPORT NEWS	VA	0.97	2.00	2.06	3.34	2.73	3.15	0.00	0.01	2.87
NE	NORFOLK	VA	0.96	1.61	2.65	1.64	2.83	3.82	0.00	0.00	1.64
NE	ONANCOCK	VA	0.98	1.55	2.66	2.32	2.81	3.80	0.00	0.00	2.37
NE	OTH CITY OF WILLIAMSBURG	VA	0.88	1.67	2.17	1.33	2.99	3.99	0.00	0.00	1.44
NE	POQUOSON	VA	0.98	1.60	2.71	1.91	2.89	3.89	0.00	0.00	1.64
NE	POTOMAC BEACH	VA	0.99	1.67	2.24	1.36	3.00	4.00	0.00	0.00	1.03
NE	QUINBY	VA	1.00	1.66	2.35	3.58	3.83	4.00	0.00	0.00	1.45
NE	REEDVILLE	VA	1.00	1.47	2.47	2.27	1.56	2.55	0.30	0.07	1.28
NE	REMLIK	VA	1.00	1.54	2.70	2.54	3.00	4.00	0.00	0.00	1.34
NE	RESCUE	VA	1.00	1.41	2.57	3.79	3.00	4.00	0.00	0.00	1.08
NE	SALUDA	VA	1.00	1.40	2.61	3.11	2.77	3.77	0.00	0.00	1.31
NE	SANFORD	VA	1.00	2.44	1.80	4.00	3.00	3.00	0.00	0.00	1.00
NE	SAXIS	VA	1.00	1.47	2.63	3.16	2.99	3.99	0.00	0.00	1.27
NE	SEAFORD	VA	1.00	2.44	1.80	4.00	3.00	3.00	0.00	0.01	1.00
NE	SEVERN	VA	1.00	1.49	2.71	2.08	2.72	3.72	0.00	0.00	1.09
NE	SHARPS	VA	1.00	1.63	2.79	1.70	3.00	4.00	0.00	0.00	1.09
NE	SMITHFIELD	VA	0.98	1.51	2.67	2.87	3.00	4.00	0.00	0.00	1.56
NE	TANGIER	VA	1.00	1.60	2.76	1.97	3.00	4.00	0.00	0.00	1.41
NE	TAPPAHANNOCK	VA	0.96	1.66	2.48	1.46	3.00	4.00	0.00	0.00	1.24
NE	TETOTUM	VA	0.97	1.66	2.27	1.37	3.00	4.00	0.00	0.00	1.44
NE	THE GLEBE	VA	1.00	1.68	1.96	1.24	3.00	4.00	0.00	0.00	1.00
NE	TOPPING	VA	1.00	1.40	2.56	3.87	3.00	4.00	0.00	0.00	1.01
NE	URBANNA	VA	0.99	1.53	2.57	2.65	2.94	3.94	0.00	0.00	1.76
NE	VIRGINIA BEACH	VA	0.68	1.53	2.32	1.56	1.98	2.67	0.00	0.00	5.59
NE	WACHAPREAGUE	VA	0.97	1.58	2.28	1.82	2.59	3.51	0.00	0.00	2.71
NE	WAKE	VA	0.91	1.54	2.62	2.54	2.99	3.99	0.00	0.00	1.99
NE	WASHINGTON	VA	0.93	1.65	2.46	1.50	3.00	4.00	0.00	0.00	1.89
NE	WATER VIEW	VA	0.99	1.52	2.54	2.73	3.00	4.00	0.00	0.00	1.61
NE	WEEMS	VA	1.00	1.40	2.56	3.87	3.00	4.00	0.00	0.00	1.01
NE	WEST POINT	VA	0.97	1.64	2.77	1.59	3.00	4.00	0.00	0.00	1.16
NE	WESTVILLE	VA	0.99	1.54	2.55	2.15	2.64	3.62	0.00	0.00	3.29
NE	WHITE STONE	VA	1.00	1.49	2.57	2.72	2.72	3.72	0.00	0.00	2.21
NE	WIDEWATER	VA	0.18	1.61	2.37	1.36	2.85	3.84	0.00	0.00	1.42
NE	YORK	VA	1.00	1.60	2.76	2.01	3.00	4.00	0.00	0.00	1.34

S2 Table 2. Southeast Region - South Atlantic subregion communities. Five-year averages (2018-2022) for all coastal fishing communities in the South Atlantic (SE/SA) subregion of the Southeast Region with any landings of species with CVAs, including region, community name, state, percent classified species in terms of value, sensitivity to temperature, stock size/status, ocean acidification, total sensitivity and total vulnerability, regional quotient for pounds and value, and Simpsons' Reciprocal diversity scores for value.

Region	COMMUNITY	ST	% Class	Temp	Stock	OA	TSens	TVuln	RQLb	RQVal	DVal
SE/SA	ASTOR	FL	1.00	1.16	1.92	2.84	2.00	3.00	0.00	0.00	1.00
SE/SA	ATLANTIC BEACH	FL	0.98	1.79	1.26	3.25	2.67	3.67	0.03	0.04	2.31
SE/SA	BOCA RATON	FL	0.28	1.74	2.10	2.29	2.00	3.00	0.00	0.00	3.30
SE/SA	BOYNTON BEACH	FL	0.19	1.79	1.81	1.85	1.45	2.45	0.00	0.00	1.59
SE/SA	BUNNELL	FL	0.77	1.10	1.91	2.64	1.95	2.95	0.00	0.00	1.80
SE/SA	CALLAHAN	FL	0.96	1.39	1.63	3.07	2.34	3.34	0.00	0.00	1.56
SE/SA	CAPE CANAVERAL	FL	0.88	1.17	1.90	2.49	1.75	2.75	0.00	0.00	2.06
SE/SA	COCOA	FL	0.85	1.73	1.55	1.54	1.20	2.20	0.03	0.03	3.56
SE/SA	COCOA BEACH	FL	0.83	1.16	1.92	2.84	2.00	3.00	0.00	0.00	1.39
SE/SA	COOPER CITY	FL	1.00	2.36	1.84	2.04	2.00	3.00	0.00	0.00	1.00
SE/SA	COPELAND	FL	1.00	1.16	1.92	2.84	2.00	3.00	0.00	0.00	1.00
SE/SA	CORAL GABLES	FL	0.72	1.64	1.44	3.84	3.00	4.00	0.00	0.00	1.44
SE/SA	CRESCENT CITY	FL	0.99	1.16	1.92	2.84	2.00	3.00	0.00	0.00	1.02
SE/SA	DANIA BEACH	FL	0.66	1.82	1.66	3.40	2.80	3.80	0.00	0.00	3.33
SE/SA	DAVIE	FL	0.77	1.66	1.40	3.80	3.00	4.00	0.00	0.00	1.38
SE/SA	DAYTONA BEACH	FL	0.79	1.89	2.03	2.01	1.87	2.87	0.01	0.01	7.08
SE/SA	DAYTONA BEACH SHORES	FL	0.98	2.03	2.57	2.01	2.10	3.10	0.00	0.00	4.77
SE/SA	DEERFIELD BEACH	FL	0.59	1.65	1.50	2.47	1.87	2.87	0.00	0.00	9.06
SE/SA	DELRAY BEACH	FL	0.51	2.15	2.27	2.35	2.39	3.39	0.00	0.00	5.23
SE/SA	EAST PALATKA	FL	1.00	1.16	1.92	2.84	2.00	3.00	0.00	0.00	1.00
SE/SA	EDGEWATER	FL	0.05	1.44	1.42	2.34	1.97	2.97	0.03	0.00	1.16
SE/SA	ELKTON	FL	0.79	1.29	2.28	2.11	1.95	2.95	0.00	0.00	3.93
SE/SA	FELLSMERE	FL	0.81	1.16	1.92	2.84	2.00	3.00	0.00	0.00	1.45
SE/SA	FERNANDINA BEACH	FL	0.90	1.79	1.15	3.49	2.93	3.93	0.01	0.01	2.02
SE/SA	FLAGLER BEACH	FL	0.99	1.16	1.92	2.84	2.00	3.00	0.00	0.00	1.02
SE/SA	FLORIDA CITY	FL	0.99	1.64	1.44	3.84	3.00	4.00	0.00	0.02	1.02
SE/SA	FORT LAUDERDALE	FL	0.68	2.12	1.93	2.57	2.37	3.37	0.00	0.01	7.89
SE/SA	FORT PIERCE	FL	0.83	1.71	1.49	1.54	1.18	2.18	0.02	0.01	4.29
SE/SA	GRANT-VALKARIA	FL	1.00	1.16	1.92	2.84	2.00	3.00	0.00	0.00	1.00
SE/SA	GREEN COVE SPRINGS	FL	0.99	1.46	1.55	3.14	2.45	3.45	0.00	0.00	1.97
SE/SA	HASTINGS	FL	1.00	1.16	1.92	2.84	2.00	3.00	0.00	0.00	1.00
SE/SA	HIALEAH	FL	0.55	2.05	1.72	2.54	2.24	3.24	0.04	0.00	3.23
SE/SA	HIALEAH GARDENS	FL	1.00	1.64	1.44	3.70	2.89	3.89	0.00	0.00	1.14
SE/SA	HILLSBORO BEACH	FL	1.00	2.24	3.96	2.36	3.00	4.00	0.00	0.00	1.00
SE/SA	HOBE SOUND	FL	1.00	1.64	1.47	3.82	2.99	3.99	0.00	0.00	1.03
SE/SA	HOLLY HILL	FL	1.00	1.16	1.92	2.84	2.00	3.00	0.00	0.00	1.00
SE/SA	HOLLYWOOD	FL	0.41	1.89	1.67	3.19	2.66	3.66	0.00	0.02	2.43
SE/SA	HOMESTEAD	FL	0.76	1.69	1.46	3.63	2.87	3.87	0.02	0.01	2.18
SE/SA	INDIALANTIC	FL	0.41	1.33	1.85	1.55	1.23	2.23	0.00	0.00	3.93
SE/SA	JACKSONVILLE	FL	0.92	1.66	1.42	3.23	2.55	3.55	0.06	0.04	3.53
SE/SA	JACKSONVILLE BEACH	FL	0.98	1.61	2.10	2.14	1.59	2.59	0.00	0.00	5.84
SE/SA	JENSEN BEACH	FL	0.10	1.69	1.87	2.13	1.71	2.71	0.00	0.00	3.10
SE/SA	JUNO BEACH	FL	0.68	2.36	1.84	2.04	2.00	3.00	0.00	0.00	1.48
SE/SA	JUPITER	FL	0.66	1.88	1.89	2.51	2.21	3.21	0.00	0.00	10.04

SE/SA	JUPITER INLET COLONY	FL	1.00	1.36	1.40	1.60	1.00	2.00	0.00	0.00	1.00
SE/SA	LAKE GENEVA	FL	0.92	1.13	1.73	1.18	1.01	2.01	0.00	0.00	1.31
SE/SA	LAKE MONROE	FL	0.99	1.16	1.92	2.84	2.00	3.00	0.00	0.00	1.02
SE/SA	LAKE PARK	FL	0.08	2.14	1.93	1.93	1.98	2.98	0.01	0.02	4.46
SE/SA	LAKE WORTH	FL	0.17	1.65	1.43	3.43	2.66	3.66	0.00	0.00	1.64
SE/SA	LANTANA	FL	1.00	1.64	1.44	3.84	3.00	4.00	0.00	0.00	1.00
SE/SA	LAUDERHILL	FL	0.88	1.81	1.56	3.13	2.55	3.55	0.00	0.00	1.98
SE/SA	MALABAR	FL	1.00	1.64	1.83	1.18	1.01	2.01	0.00	0.00	1.15
SE/SA	MARGATE	FL	0.11	1.66	1.45	1.42	1.02	2.02	0.00	0.01	1.46
SE/SA	MEDLEY	FL	1.00	1.64	1.44	3.84	3.00	4.00	0.00	0.01	1.00
SE/SA	MELBOURNE	FL	0.66	1.64	2.07	1.99	1.77	2.77	0.00	0.00	10.51
SE/SA	MELBOURNE BEACH	FL	0.25	1.59	1.87	1.86	1.51	2.51	0.00	0.00	2.94
SE/SA	MERRITT ISLAND	FL	0.98	1.21	1.95	2.59	1.85	2.85	0.00	0.00	1.39
SE/SA	MIAMI	FL	0.79	1.75	1.53	3.50	2.79	3.79	0.02	0.04	2.30
SE/SA	MIAMI BEACH	FL	0.41	1.95	1.90	2.54	2.19	3.19	0.00	0.00	2.37
SE/SA	MIAMI GARDENS	FL	0.99	2.33	1.92	2.09	2.04	3.04	0.00	0.00	1.24
SE/SA	MIAMI LAKES	FL	0.58	1.64	1.44	3.84	3.00	4.00	0.00	0.00	1.80
SE/SA	MICCO	FL	1.00	1.16	1.81	1.50	1.10	2.10	0.00	0.00	2.24
SE/SA	MIDDLEBURG	FL	0.95	1.16	1.92	2.84	2.00	3.00	0.00	0.00	1.13
SE/SA	NEPTUNE BEACH	FL	0.68	1.16	1.92	2.84	2.00	3.00	0.00	0.00	1.02
SE/SA	NEW SMYRNA BEACH	FL	0.40	1.60	2.15	1.89	1.72	2.72	0.01	0.00	5.20
SE/SA	NORTH LAUDERDALE	FL	1.00	1.16	1.92	2.84	2.00	3.00	0.00	0.00	1.00
SE/SA	NORTH PALM BEACH	FL	1.00	2.28	1.83	1.97	1.91	2.91	0.00	0.00	1.33
SE/SA	OAK HILL	FL	0.33	1.16	1.74	1.95	1.45	2.45	0.02	0.00	3.05
SE/SA	OAKLAND PARK	FL	1.00	2.44	1.80	2.56	2.00	3.00	0.00	0.00	1.00
SE/SA	OPA LOCKA	FL	0.92	1.65	1.46	1.45	1.03	2.03	0.00	0.00	1.34
SE/SA	ORANGE PARK	FL	0.99	1.19	1.89	2.80	2.03	3.03	0.00	0.00	1.16
SE/SA	ORLANDO	FL	0.73	1.31	1.81	2.43	1.98	2.98	0.00	0.00	3.45
SE/SA	ORMOND BEACH	FL	0.86	1.41	2.14	2.27	1.88	2.88	0.00	0.00	5.87
SE/SA	OSTEEN	FL	0.60	1.39	1.93	2.57	2.00	3.00	0.00	0.00	3.07
SE/SA	PALATKA	FL	1.00	1.16	1.91	2.84	2.00	3.00	0.00	0.00	1.02
SE/SA	PALM BAY	FL	0.98	1.16	1.92	2.64	1.86	2.86	0.00	0.00	1.22
SE/SA	PALM BEACH GARDENS	FL	0.81	1.85	1.57	2.54	2.09	3.09	0.00	0.00	3.32
SE/SA	PALM CITY	FL	1.00	1.16	1.92	2.84	2.00	3.00	0.00	0.00	1.00
SE/SA	PALM COAST	FL	0.51	1.51	2.10	2.18	1.76	2.76	0.00	0.00	3.53
SE/SA	PALMETTO BAY	FL	0.90	2.29	1.83	2.00	1.94	2.94	0.00	0.00	1.50
SE/SA	PARKLAND	FL	1.00	1.64	1.44	3.84	3.00	4.00	0.00	0.00	1.00
SE/SA	PLANTATION	FL	0.97	1.71	1.53	1.96	1.51	2.51	0.00	0.00	2.37
SE/SA	POMPANO BEACH	FL	0.03	1.34	1.53	1.75	1.40	2.40	0.00	0.00	2.70
SE/SA	PORT ORANGE	FL	0.77	1.85	1.89	1.92	1.88	2.88	0.01	0.01	10.47
SE/SA	PORT SAINT JOHN	FL	1.00	1.16	1.92	2.82	1.99	2.99	0.00	0.00	1.04
SE/SA	PORT SAINT LUCIE	FL	0.20	1.32	1.83	2.42	1.69	2.69	0.00	0.00	2.42
SE/SA	PORT SALERNO	FL	0.44	1.69	1.29	1.37	1.00	2.00	0.00	0.00	2.02
SE/SA	RIVIERA BEACH	FL	1.00	1.64	1.44	3.83	2.99	3.99	0.00	0.00	1.01
SE/SA	ROCKLEDGE	FL	0.86	1.17	1.88	2.34	1.73	2.73	0.00	0.00	2.82
SE/SA	SAINT AUGUSTINE	FL	0.97	1.30	2.04	2.85	2.17	3.17	0.01	0.01	2.84
SE/SA	SANFORD	FL	0.98	2.13	3.20	2.16	2.53	3.53	0.00	0.00	4.35
SE/SA	SATELLITE BEACH	FL	0.95	1.88	2.13	2.32	2.17	3.17	0.00	0.00	4.75
SE/SA	SEBASTIAN	FL	0.84	1.55	1.85	2.16	1.82	2.82	0.00	0.00	8.56
SE/SA	SHARPES	FL	0.61	1.32	3.24	3.44	3.00	4.00	0.00	0.00	1.17

SE/SA	SOUTHWEST RANCHES	FL	0.75	1.63	1.43	2.49	1.87	2.87	0.00	0.00	2.71
SE/SA	STUART	FL	0.28	1.50	1.79	3.08	2.40	3.40	0.00	0.00	3.36
SE/SA	SUNRISE	FL	0.61	1.66	1.52	3.44	2.70	3.70	0.00	0.00	2.73
SE/SA	TAMARAC	FL	1.00	1.64	1.44	3.84	3.00	4.00	0.00	0.00	1.00
SE/SA	TEQUESTA	FL	0.36	1.76	1.24	1.40	1.00	2.00	0.00	0.00	1.71
SE/SA	TITUSVILLE	FL	0.90	1.92	1.60	3.30	2.55	3.55	0.02	0.02	3.55
SE/SA	VERO BEACH	FL	0.46	1.16	1.72	1.42	1.14	2.14	0.00	0.00	2.05
SE/SA	WABASSO	FL	0.65	1.18	1.78	1.41	1.00	2.00	0.00	0.00	3.04
SE/SA	WEST MELBOURNE	FL	1.00	1.55	1.58	3.45	2.66	3.66	0.00	0.00	1.68
SE/SA	WEST PALM BEACH	FL	0.72	2.28	1.75	2.60	2.05	3.05	0.00	0.01	2.89
SE/SA	YULEE	FL	1.00	1.81	1.09	3.49	3.00	4.00	0.00	0.00	1.46
SE/SA	ALMA	GA	1.00	1.39	1.81	2.40	1.80	2.80	0.00	0.00	1.86
SE/SA	ATLANTA	GA	0.72	1.67	1.17	1.61	1.00	2.00	0.00	0.00	1.62
SE/SA	BAXLEY	GA	0.97	2.28	3.40	1.08	3.00	4.00	0.00	0.00	1.05
SE/SA	BLOOMINGDALE	GA	1.00	1.16	1.92	2.84	2.00	3.00	0.00	0.00	1.01
SE/SA	BRUNSWICK	GA	0.93	1.76	1.18	3.44	2.88	3.88	0.01	0.02	1.63
SE/SA	CRESCENT	GA	1.00	1.17	1.90	2.85	2.02	3.02	0.00	0.00	1.04
SE/SA	DARIEN	GA	0.98	1.62	1.34	3.30	2.69	3.69	0.01	0.02	1.94
SE/SA	ELLABELL	GA	1.00	1.16	1.92	2.84	2.00	3.00	0.00	0.00	1.00
SE/SA	FLEMING	GA	1.00	1.76	1.08	3.46	3.00	4.00	0.00	0.00	2.00
SE/SA	GARDEN CITY	GA	1.00	1.16	1.92	2.84	2.00	3.00	0.00	0.00	1.00
SE/SA	GLENNVILLE	GA	0.80	2.28	3.38	1.09	3.00	4.00	0.00	0.00	1.54
SE/SA	GLENWOOD	GA	1.00	1.83	1.08	3.51	3.00	4.00	0.00	0.00	1.22
SE/SA	HAZLEHURST	GA	0.94	2.28	3.40	1.08	3.00	4.00	0.00	0.00	1.13
SE/SA	HINESVILLE	GA	1.00	1.16	1.92	2.84	2.00	3.00	0.00	0.00	1.00
SE/SA	HORTENSE	GA	1.00	1.16	1.92	2.84	2.00	3.00	0.00	0.00	1.00
SE/SA	JESUP	GA	0.95	1.19	1.90	2.85	2.04	3.04	0.00	0.00	1.20
SE/SA	KINGSLAND	GA	1.00	1.16	1.92	2.84	2.00	3.00	0.00	0.00	1.00
SE/SA	LUDOWICI	GA	0.87	2.28	3.40	1.08	3.00	4.00	0.00	0.00	1.34
SE/SA	MELDRIM	GA	1.00	1.84	1.08	3.52	3.00	4.00	0.00	0.00	1.00
SE/SA	MIDWAY	GA	0.82	1.16	1.94	2.85	2.01	3.01	0.00	0.00	1.45
SE/SA	NAHUNTA	GA	0.98	1.34	1.69	3.02	2.27	3.27	0.00	0.00	1.71
SE/SA	ODUM	GA	1.00	2.28	3.40	1.08	3.00	4.00	0.00	0.00	1.00
SE/SA	POOLER	GA	1.00	1.84	1.08	3.52	3.00	4.00	0.00	0.00	1.00
SE/SA	PORT WENTWORTH	GA	0.76	2.28	3.40	1.08	3.00	4.00	0.00	0.00	1.48
SE/SA	REIDSVILLE	GA	0.94	2.28	3.40	1.08	3.00	4.00	0.00	0.00	1.13
SE/SA	RICEBORO	GA	1.00	1.16	1.92	2.84	2.00	3.00	0.00	0.00	1.00
SE/SA	RICHMOND HILL	GA	0.75	1.20	1.86	2.88	2.07	3.07	0.00	0.00	1.84
SE/SA	SAINT MARYS	GA	0.92	1.17	1.91	2.85	2.01	3.01	0.00	0.00	1.20
SE/SA	SAINT SIMONS ISLAND	GA	0.21	1.81	2.85	2.00	1.66	2.66	0.00	0.00	1.52
SE/SA	SAPELO ISLAND	GA	1.00	1.16	1.92	2.84	2.00	3.00	0.00	0.00	1.00
SE/SA	SAVANNAH	GA	0.95	1.47	1.54	3.15	2.46	3.46	0.01	0.01	2.18
SE/SA	SOPERTON	GA	1.00	1.16	1.92	2.84	2.00	3.00	0.00	0.00	1.00
SE/SA	SPRINGFIELD	GA	1.00	1.16	1.92	2.84	2.00	3.00	0.00	0.00	1.00
SE/SA	SURRENCY	GA	0.93	2.28	3.40	1.08	3.00	4.00	0.00	0.00	1.17
SE/SA	SWAINSBORO	GA	1.00	1.84	1.08	3.52	3.00	4.00	0.00	0.00	1.00
SE/SA	SYLVANIA	GA	0.98	2.28	3.40	1.08	3.00	4.00	0.00	0.00	1.04
SE/SA	TOWNSEND	GA	0.65	1.72	1.29	3.39	2.82	3.82	0.02	0.04	2.65
SE/SA	TYBEE ISLAND	GA	0.98	1.84	1.08	3.52	3.00	4.00	0.00	0.01	1.12
SE/SA	UVALDA	GA	0.93	2.28	3.40	1.08	3.00	4.00	0.00	0.00	1.15
SE/SA	VALONA	GA	1.00	1.50	1.50	3.18	2.50	3.50	0.00	0.00	1.00
SE/SA	WAVERLY	GA	0.11	1.11	2.30	1.79	2.00	3.00	0.00	0.00	1.17
SE/SA	WAYNESVILLE	GA	1.00	1.16	1.92	2.84	2.00	3.00	0.00	0.00	1.00

SE/SA	WOODBINE	GA	1.00	1.19	2.05	2.92	2.13	3.13	0.00	0.00	1.32
SE/SA	ARAPAHOE	NC	1.00	1.13	2.12	2.29	2.00	3.00	0.00	0.00	1.67
SE/SA	ASH	NC	0.95	1.60	1.37	2.62	2.17	3.17	0.00	0.00	1.67
SE/SA	ATLANTIC	NC	0.72	1.47	2.23	3.17	2.76	3.76	0.00	0.00	2.98
SE/SA	ATLANTIC BEACH	NC	0.25	1.60	1.88	1.66	1.34	2.34	0.00	0.00	3.90
SE/SA	AURORA	NC	0.97	1.21	1.89	2.63	1.98	2.98	0.00	0.00	1.41
SE/SA	BARCO	NC	0.91	1.17	1.93	2.81	2.00	3.00	0.02	0.01	1.26
SE/SA	BATH	NC	1.00	1.26	2.72	3.20	2.60	3.60	0.00	0.00	1.00
SE/SA	BAYBORO	NC	1.00	1.28	1.74	2.97	2.22	3.22	0.00	0.00	1.54
SE/SA	BEAUFORT	NC	0.48	1.67	1.56	2.82	2.33	3.33	0.05	0.06	5.02
SE/SA	BELHAVEN	NC	0.98	1.19	1.94	2.81	2.06	3.06	0.00	0.00	1.30
SE/SA	BELVIDERE	NC	0.87	1.80	2.41	2.02	2.52	3.52	0.00	0.00	3.09
SE/SA	BOLIVIA	NC	0.87	1.44	1.75	3.17	2.51	3.51	0.00	0.00	2.54
SE/SA	BURGAW	NC	1.00	1.16	1.92	2.84	2.00	3.00	0.00	0.00	1.00
SE/SA	BUXTON	NC	0.97	1.29	2.97	3.13	2.70	3.70	0.00	0.00	1.62
SE/SA	CALABASH	NC	1.00	1.73	1.52	3.48	2.97	3.97	0.00	0.00	1.53
SE/SA	CAPE CARTERET	NC	0.74	1.31	2.01	2.26	1.98	2.98	0.00	0.00	1.96
SE/SA	CAROLINA BEACH	NC	0.78	1.20	1.87	2.85	2.04	3.04	0.00	0.00	1.79
SE/SA	CEDAR ISLAND	NC	0.63	1.50	1.51	2.65	2.23	3.23	0.01	0.01	6.75
SE/SA	CEDAR POINT	NC	1.00	1.79	1.08	3.48	3.00	4.00	0.00	0.00	1.00
SE/SA	CHARLOTTE	NC	0.94	2.20	2.39	2.09	2.27	3.27	0.00	0.00	4.68
SE/SA	CHOCOWINITY	NC	0.78	1.37	1.76	1.19	1.50	2.50	0.00	0.00	1.64
SE/SA	COINJOCK	NC	1.00	1.16	1.92	2.84	2.00	3.00	0.00	0.00	1.00
SE/SA	COLUMBIA	NC	0.99	1.17	1.92	2.82	2.00	3.00	0.03	0.02	1.05
SE/SA	COROLLA	NC	0.91	1.74	1.10	3.40	2.96	3.96	0.00	0.00	1.69
SE/SA	COVE CITY	NC	0.99	1.83	1.08	3.50	2.99	3.99	0.00	0.00	1.13
SE/SA	CURRIE	NC	1.00	1.68	1.08	3.40	3.00	4.00	0.00	0.00	1.00
SE/SA	CURRITUCK	NC	0.95	1.18	1.89	2.86	2.03	3.03	0.01	0.00	1.19
SE/SA	DARE	NC	1.00	1.16	1.92	2.84	2.00	3.00	0.00	0.00	1.00
SE/SA	DAVIS	NC	1.00	1.19	1.87	2.85	2.04	3.04	0.00	0.00	1.16
SE/SA	DUCK	NC	0.90	1.14	1.76	1.25	1.10	2.10	0.00	0.00	1.56
SE/SA	EDENTON	NC	0.28	2.10	2.56	1.50	2.62	3.62	0.00	0.00	3.93
SE/SA	ELIZABETH CITY	NC	1.00	1.16	1.94	2.78	2.00	3.00	0.00	0.00	1.09
SE/SA	EMERALD ISLE	NC	0.73	1.81	1.57	3.02	2.69	3.69	0.00	0.00	3.35
SE/SA	ENGELHARD	NC	0.83	1.72	1.24	3.27	2.75	3.75	0.02	0.03	2.96
SE/SA	FRISCO	NC	0.99	1.12	1.72	1.16	1.00	2.00	0.00	0.00	1.02
SE/SA	GARLAND	NC	0.92	1.33	3.18	3.43	2.99	3.99	0.00	0.00	1.29
SE/SA	GEORGETOWN	NC	1.00	1.16	1.92	2.84	2.00	3.00	0.00	0.00	1.00
SE/SA	GLOUCESTER	NC	1.00	1.17	1.90	2.86	2.03	3.03	0.00	0.00	1.06
SE/SA	GRANDY	NC	1.00	1.16	1.92	2.84	2.00	3.00	0.00	0.00	1.00
SE/SA	GRANTSBORO	NC	1.00	1.66	1.49	3.44	3.00	4.00	0.00	0.00	2.01
SE/SA	HAMPSTEAD	NC	0.81	1.35	2.10	2.55	1.95	2.95	0.01	0.01	4.74
SE/SA	HARBINGER	NC	1.00	1.16	1.92	2.84	2.00	3.00	0.00	0.00	1.00
SE/SA	HARKERS ISLAND	NC	0.86	1.37	1.62	3.00	2.32	3.32	0.00	0.00	2.91
SE/SA	HATTERAS	NC	0.85	1.45	1.70	2.02	1.55	2.55	0.04	0.02	5.90
SE/SA	HAVELOCK	NC	0.67	1.38	2.57	3.23	2.82	3.82	0.00	0.00	2.23
SE/SA	HENDERSONVILLE	NC	0.91	1.40	1.45	1.46	1.00	2.00	0.00	0.00	1.90
SE/SA	HERTFORD	NC	0.96	1.22	1.96	2.75	2.04	3.04	0.01	0.01	1.24
SE/SA	HOBUCKEN	NC	0.83	1.78	1.17	3.28	2.84	3.84	0.01	0.01	2.62
SE/SA	HOLDEN BEACH	NC	0.15	1.15	1.89	2.73	1.96	2.96	0.00	0.00	1.36
SE/SA	HOLLY RIDGE	NC	0.62	1.41	2.31	2.84	2.44	3.44	0.00	0.00	3.60
SE/SA	HOOKERTON	NC	1.00	1.16	1.92	2.84	2.00	3.00	0.00	0.00	1.00
SE/SA	HUBERT	NC	0.78	1.25	2.24	2.57	2.13	3.13	0.00	0.00	3.20
SE/SA	JACKSONVILLE	NC	0.96	1.30	2.12	2.39	2.00	3.00	0.00	0.00	4.04

SE/SA	JARVISBURG	NC	1.00	1.16	1.92	2.84	2.00	3.00	0.00	0.00	1.01
SE/SA	KILL DEVIL HILLS	NC	0.99	1.17	1.92	2.84	2.01	3.01	0.01	0.01	1.06
SE/SA	KITTY HAWK	NC	0.78	1.27	2.07	2.56	2.09	3.09	0.00	0.00	3.10
SE/SA	KNOTTS ISLAND	NC	1.00	1.16	1.92	2.83	2.00	3.00	0.00	0.00	1.01
SE/SA	KURE BEACH	NC	1.00	1.32	3.24	3.44	3.00	4.00	0.00	0.00	1.00
SE/SA	LA GRANGE	NC	0.78	1.57	1.31	3.06	2.73	3.73	0.00	0.00	1.69
SE/SA	LAKE WACCAMAW	NC	1.00	1.32	3.24	3.44	3.00	4.00	0.00	0.00	1.00
SE/SA	LELAND	NC	0.85	1.33	2.86	3.03	2.65	3.65	0.00	0.00	2.42
SE/SA	LEXINGTON	NC	1.00	1.73	1.23	3.04	2.54	3.54	0.00	0.00	1.27
SE/SA	LOWLAND	NC	1.00	1.34	2.23	3.17	2.53	3.53	0.00	0.00	2.06
SE/SA	MANNS HARBOR	NC	0.92	1.21	1.87	2.87	2.08	3.08	0.00	0.00	1.40
SE/SA	MANTEO	NC	0.47	1.55	1.24	2.77	2.27	3.27	0.00	0.00	1.57
SE/SA	MAPLE	NC	1.00	1.16	1.92	2.84	2.00	3.00	0.00	0.00	1.00
SE/SA	MARSHALLBERG	NC	1.00	1.32	3.24	3.44	3.00	4.00	0.00	0.00	1.00
SE/SA	MERRITT	NC	0.94	1.30	1.82	2.17	1.88	2.88	0.00	0.00	2.95
SE/SA	MOREHEAD CITY	NC	0.81	1.87	2.23	2.16	1.99	2.99	0.00	0.00	11.00
SE/SA	MOUNT PLEASANT	NC	1.00	1.83	1.08	3.51	3.00	4.00	0.00	0.00	1.12
SE/SA	MOYOCK	NC	0.99	1.16	1.92	2.84	2.00	3.00	0.00	0.00	1.01
SE/SA	NAGS HEAD	NC	0.59	1.22	1.82	2.13	1.64	2.64	0.00	0.00	4.28
SE/SA	NEW BERN	NC	0.75	1.26	1.95	2.57	1.97	2.97	0.00	0.00	2.65
SE/SA	NEWPORT	NC	0.97	1.32	2.85	3.23	2.71	3.71	0.00	0.00	2.01
SE/SA	NORTH TOPSAIL BEACH	NC	1.00	1.68	1.08	3.40	3.00	4.00	0.00	0.00	1.00
SE/SA	OAK ISLAND	NC	0.91	1.53	2.38	2.58	2.20	3.20	0.00	0.00	3.68
SE/SA	OCEAN ISLE BEACH	NC	0.87	1.30	2.25	2.90	2.25	3.25	0.00	0.00	2.42
SE/SA	OCRACOKE	NC	0.69	1.48	1.85	1.91	1.60	2.60	0.00	0.00	6.06
SE/SA	ORIENTAL	NC	0.90	1.70	1.22	3.30	2.81	3.81	0.02	0.03	2.56
SE/SA	PANTEGO	NC	1.00	1.16	1.92	2.84	2.00	3.00	0.00	0.00	1.01
SE/SA	PINK HILL	NC	1.00	1.92	2.80	2.79	2.94	3.94	0.00	0.00	1.19
SE/SA	POPLAR BRANCH	NC	0.86	1.17	1.92	2.83	2.01	3.01	0.00	0.00	1.37
SE/SA	POWELLS POINT	NC	1.00	1.16	1.92	2.84	2.00	3.00	0.00	0.00	1.00
SE/SA	RALEIGH	NC	0.98	1.29	1.80	3.00	2.29	3.29	0.00	0.00	1.74
SE/SA	REIDSVILLE	NC	0.92	2.54	2.94	2.08	2.55	3.55	0.00	0.00	2.55
SE/SA	RICHLANDS	NC	0.99	1.33	2.49	2.41	2.05	3.05	0.00	0.00	1.50
SE/SA	RIEGELWOOD	NC	1.00	2.28	3.40	1.08	3.00	4.00	0.00	0.00	1.00
SE/SA	ROCKY POINT	NC	0.81	1.79	1.94	2.33	2.08	3.08	0.00	0.00	10.68
SE/SA	RODANTHE	NC	0.97	1.13	1.77	1.57	1.25	2.25	0.00	0.00	1.24
SE/SA	ROWLAND	NC	1.00	1.34	1.73	1.49	1.07	2.07	0.00	0.00	1.15
SE/SA	ROXBORO	NC	0.94	1.56	2.00	2.09	1.43	2.43	0.00	0.00	4.04
SE/SA	SALTER PATH	NC	1.00	1.43	1.97	3.17	2.58	3.58	0.00	0.00	3.12
SE/SA	SALVO	NC	0.98	1.13	1.77	1.54	1.23	2.23	0.00	0.00	1.57
SE/SA	SANFORD	NC	0.78	1.28	1.86	2.22	1.66	2.66	0.00	0.00	4.21
SE/SA	SCRANTON	NC	1.00	1.44	2.73	3.46	3.00	4.00	0.00	0.00	1.08
SE/SA	SEALEVEL	NC	0.79	1.35	2.74	2.96	2.64	3.64	0.00	0.00	3.36
SE/SA	SELMA	NC	0.10	1.59	1.61	1.17	1.97	2.97	0.00	0.00	1.21
SE/SA	SHALLOTTE	NC	0.74	1.48	2.21	3.02	2.65	3.65	0.00	0.00	4.95
SE/SA	SHILOH	NC	1.00	1.15	1.99	2.65	2.00	3.00	0.00	0.00	1.26
SE/SA	SMYRNA	NC	0.93	1.16	1.92	2.84	2.00	3.00	0.00	0.00	1.15
SE/SA	SNEADS FERRY	NC	0.82	1.62	1.52	3.17	2.62	3.62	0.01	0.02	4.73
SE/SA	SOUTHPORT	NC	0.83	1.80	1.85	2.07	1.66	2.66	0.00	0.00	6.96
SE/SA	STACY	NC	1.00	1.77	1.08	3.47	3.00	4.00	0.00	0.00	1.93
SE/SA	STELLA	NC	0.78	1.39	1.85	2.39	2.11	3.11	0.00	0.00	5.64
SE/SA	STUMPY POINT	NC	0.80	1.47	1.55	2.77	2.25	3.25	0.01	0.01	4.70
SE/SA	SUNSET BEACH	NC	0.82	1.31	2.41	2.48	2.12	3.12	0.00	0.00	4.55

SE/SA	SUPPLY	NC	0.97	1.80	1.58	3.06	2.61	3.61	0.00	0.01	3.27
SE/SA	SURF CITY	NC	0.75	1.62	2.55	2.82	2.51	3.51	0.00	0.00	6.09
SE/SA	SWAN QUARTER	NC	0.95	1.47	1.62	3.08	2.46	3.46	0.02	0.02	3.44
SE/SA	SWANSBORO	NC	0.68	1.70	1.36	3.37	2.89	3.89	0.00	0.00	3.38
SE/SA	TOPSAIL BEACH	NC	0.79	1.68	1.09	3.39	2.99	3.99	0.00	0.00	1.52
SE/SA	TRENTON	NC	1.00	1.08	2.48	1.28	2.00	3.00	0.00	0.00	1.00
SE/SA	TYNER	NC	0.30	2.33	2.64	1.58	3.00	4.00	0.00	0.00	2.19
SE/SA	VANDEMERE	NC	1.00	1.72	1.27	3.46	3.00	4.00	0.00	0.00	1.73
SE/SA	WALLACE	NC	1.00	1.84	1.08	3.52	3.00	4.00	0.00	0.00	1.00
SE/SA	WANCHESE	NC	0.47	1.53	1.74	2.33	1.94	2.94	0.07	0.07	12.72
SE/SA	WASHINGTON	NC	0.80	1.36	1.83	2.13	2.01	3.01	0.00	0.00	2.76
SE/SA	WHITEVILLE	NC	0.62	1.32	1.83	1.57	1.27	2.27	0.00	0.00	4.46
SE/SA	WILLISTON	NC	1.00	1.73	1.08	3.44	3.00	4.00	0.00	0.00	1.22
SE/SA	WILMINGTON	NC	0.84	1.42	2.30	2.71	2.20	3.20	0.01	0.01	5.40
SE/SA	WINDSOR	NC	0.64	2.12	2.41	1.47	2.57	3.57	0.00	0.00	2.83
SE/SA	WINNABOW	NC	0.34	1.42	1.75	1.82	1.76	2.76	0.00	0.00	3.12
SE/SA	WRIGHTSVILLE BEACH	NC	0.67	2.06	2.60	2.34	2.64	3.64	0.00	0.00	8.81
SE/SA	AIKEN	SC	1.00	1.81	1.08	3.50	3.00	4.00	0.00	0.00	1.39
SE/SA	ANDREWS	SC	1.00	2.28	3.40	1.08	3.00	4.00	0.00	0.00	1.00
SE/SA	AWENDAW	SC	1.00	1.16	1.92	2.84	2.00	3.00	0.00	0.00	1.00
SE/SA	BARNWELL	SC	0.97	1.71	1.24	3.39	2.81	3.81	0.00	0.00	1.56
SE/SA	BEAUFORT	SC	0.97	1.51	1.67	3.24	2.61	3.61	0.01	0.02	2.69
SE/SA	BLUFFTON	SC	1.00	1.37	2.67	3.34	2.82	3.82	0.00	0.00	2.04
SE/SA	BONNEAU	SC	1.00	2.10	2.45	2.08	3.00	4.00	0.00	0.00	1.90
SE/SA	BRUNSON	SC	1.00	1.20	1.87	2.88	2.06	3.06	0.00	0.00	1.13
SE/SA	BURTON	SC	1.00	1.16	1.92	2.84	2.00	3.00	0.00	0.00	1.00
SE/SA	CHARLESTON	SC	0.89	1.48	2.24	2.83	2.38	3.38	0.01	0.01	6.78
SE/SA	CONWAY	SC	1.00	1.50	1.50	3.18	2.50	3.50	0.00	0.00	1.00
SE/SA	CROSS	SC	1.00	1.16	1.92	2.84	2.00	3.00	0.00	0.00	1.00
SE/SA	DALE	SC	1.00	1.16	1.92	2.84	2.00	3.00	0.00	0.00	1.00
SE/SA	DORCHESTER	SC	1.00	1.16	1.92	2.84	2.00	3.00	0.00	0.00	1.00
SE/SA	EDISTO BEACH	SC	1.00	1.82	1.11	3.51	3.00	4.00	0.00	0.00	1.24
SE/SA	EDISTO ISLAND	SC	0.98	1.16	1.92	2.84	2.00	3.00	0.00	0.00	1.04
SE/SA	FOLLY BEACH	SC	1.00	1.32	3.24	3.44	3.00	4.00	0.00	0.00	1.00
SE/SA	GEORGETOWN	SC	1.00	1.47	1.61	3.09	2.45	3.45	0.01	0.01	2.13
SE/SA	GOOSE CREEK	SC	1.00	1.26	2.79	3.23	2.66	3.66	0.00	0.00	1.46
SE/SA	GREEN POND	SC	1.00	1.71	1.61	3.49	3.00	4.00	0.00	0.00	1.57
SE/SA	GRESHAM	SC	1.00	2.28	3.40	1.08	3.00	4.00	0.00	0.00	1.00
SE/SA	HAMPTON	SC	1.00	1.21	1.85	2.89	2.08	3.08	0.00	0.00	1.19
SE/SA	HANAHAN	SC	1.00	1.22	2.08	2.97	2.21	3.21	0.00	0.00	1.54
SE/SA	HEMINGWAY	SC	1.00	2.28	3.40	1.08	3.00	4.00	0.00	0.00	1.00
SE/SA	HILTON HEAD ISLAND	SC	0.99	1.77	1.36	3.44	2.94	3.94	0.00	0.00	1.60
SE/SA	HOLLYWOOD	SC	1.00	1.21	2.37	3.05	2.34	3.34	0.00	0.00	1.14
SE/SA	HUGER	SC	0.83	1.18	2.11	2.92	2.14	3.14	0.00	0.00	1.65
SE/SA	ISLE OF PALMS	SC	0.79	1.16	1.92	2.84	2.00	3.00	0.00	0.00	1.49
SE/SA	JAMESTOWN	SC	1.00	1.22	1.85	2.90	2.08	3.08	0.00	0.00	1.20
SE/SA	JOHNS ISLAND	SC	0.99	1.34	1.71	3.03	2.29	3.29	0.00	0.00	1.78
SE/SA	JOHNSONVILLE	SC	0.96	2.28	3.40	1.08	3.00	4.00	0.00	0.00	1.08
SE/SA	LADSON	SC	0.89	1.16	1.92	2.84	2.00	3.00	0.00	0.00	1.25
SE/SA	LEXINGTON	SC	1.00	1.16	1.96	2.86	2.03	3.03	0.00	0.00	1.06
SE/SA	LITTLE RIVER	SC	0.95	1.94	2.06	2.13	1.77	2.77	0.00	0.01	5.81
SE/SA	LONGS	SC	0.95	1.59	1.70	2.17	1.34	2.34	0.00	0.00	2.73
SE/SA	MCCLELLANVILLE	SC	0.90	1.75	1.53	3.25	2.78	3.78	0.01	0.03	2.82

SE/SA	MEGGETT	SC	0.29	1.48	1.51	1.68	1.18	2.18	0.00	0.01	3.93
SE/SA	MONCKS CORNER	SC	1.00	1.17	1.96	2.86	2.03	3.03	0.00	0.00	1.07
SE/SA	MOUNT PLEASANT	SC	0.95	1.72	1.50	3.41	2.91	3.91	0.00	0.00	2.00
SE/SA	MURRELLS INLET	SC	0.95	1.90	2.05	2.22	2.00	3.00	0.00	0.01	6.98
SE/SA	MYRTLE BEACH	SC	0.98	1.59	1.68	2.80	2.29	3.29	0.00	0.00	2.63
SE/SA	NICHOLS	SC	1.00	1.16	1.92	2.84	2.00	3.00	0.00	0.00	1.00
SE/SA	NORTH AUGUSTA	SC	1.00	1.81	1.08	3.50	3.00	4.00	0.00	0.00	1.44
SE/SA	NORTH CHARLESTON	SC	1.00	1.29	1.79	2.98	2.22	3.22	0.00	0.00	1.57
SE/SA	NORTH MYRTLE BEACH	SC	1.00	1.16	1.92	2.84	2.00	3.00	0.00	0.00	1.00
SE/SA	OKATIE	SC	1.00	1.30	1.75	2.98	2.20	3.20	0.00	0.00	1.22
SE/SA	ORANGEBURG	SC	0.99	1.16	1.92	2.84	2.00	3.00	0.00	0.00	1.02
SE/SA	PAWLEYS ISLAND	SC	1.00	1.83	1.08	3.51	3.00	4.00	0.00	0.00	1.10
SE/SA	PORT ROYAL	SC	1.00	1.84	1.08	3.52	3.00	4.00	0.00	0.00	1.03
SE/SA	RAVENEL	SC	1.00	1.34	2.97	3.39	2.91	3.91	0.00	0.00	1.39
SE/SA	RIDGELAND	SC	0.97	1.35	1.89	3.08	2.39	3.39	0.00	0.00	2.43
SE/SA	RIDGEVILLE	SC	1.00	1.31	3.16	3.40	2.94	3.94	0.00	0.00	1.14
SE/SA	ROCK HILL	SC	1.00	1.83	1.08	3.51	3.00	4.00	0.00	0.00	1.10
SE/SA	SAINT HELENA	SC	0.99	1.25	2.51	3.14	2.50	3.50	0.00	0.00	2.12
SE/SA	SAINT STEPHEN	SC	0.99	1.16	1.92	2.84	2.00	3.00	0.00	0.00	1.01
SE/SA	SEABROOK	SC	1.00	1.18	1.89	2.86	2.04	3.04	0.00	0.00	1.08
SE/SA	SEABROOK ISLAND	SC	1.00	1.32	3.23	3.44	3.00	4.00	0.00	0.00	1.01
SE/SA	SENECA	SC	0.84	1.16	1.92	2.84	2.00	3.00	0.00	0.00	1.36
SE/SA	ST. MATTHEWS	SC	1.00	1.79	1.08	3.49	3.00	4.00	0.00	0.00	1.70
SE/SA	SUMMERVILLE	SC	0.73	1.24	2.57	3.14	2.49	3.49	0.00	0.00	2.31
SE/SA	TAYLORS	SC	1.00	1.84	1.08	3.52	3.00	4.00	0.00	0.00	1.00
SE/SA	ULMER	SC	1.00	1.16	1.92	2.84	2.00	3.00	0.00	0.00	1.00
SE/SA	WADMALAW ISLAND	SC	0.30	1.42	1.49	2.30	1.67	2.67	0.00	0.00	2.35
SE/SA	WALTERBORO	SC	1.00	1.18	1.90	2.86	2.03	3.03	0.00	0.00	1.05
SE/SA	YEMASSEE	SC	1.00	1.16	1.92	2.84	2.00	3.00	0.00	0.00	1.00

S2 Table 3. Southeast Region - Gulf of America/Florida Keys subregion communities. Five-year averages (2018-2022) for all coastal fishing communities in the Gulf of America (SE/GoA)/Florida Keys (SE/KS) subregion of the Southeast Region with any landings of species with CVAs, including region, community name, state, percent classified species in terms of value, sensitivity to temperature, stock size/status, ocean acidification, total sensitivity and total vulnerability, regional quotient for pounds and value, and Simpsons' Reciprocal diversity scores for value.

Region	COMMUNITY	ST	% Class	Temp	Stock	OA	TSens	TVuln	RQLb	RQVal	DVal
SE/GoA	BAYOU LA BATRE	AL	0.99	1.91	1.23	1.86	1.03	1.04	0.02	0.05	2.83
SE/GoA	BON SECOUR	AL	0.93	1.85	1.50	1.93	1.20	1.21	0.00	0.01	6.27
SE/GoA	CHATOM	AL	1.00	1.88	1.20	1.80	1.00	1.00	0.00	0.00	1.00
SE/GoA	CODEN	AL	0.99	1.82	1.45	2.18	1.28	1.28	0.00	0.01	5.07
SE/GoA	DAUPHIN ISLAND	AL	0.96	1.83	1.44	1.76	1.16	1.16	0.00	0.00	1.86
SE/GoA	EIGHT MILE	AL	1.00	1.47	1.85	2.22	1.00	1.00	0.00	0.00	1.32
SE/GoA	ELBERTA	AL	0.99	1.88	1.21	1.80	1.00	1.00	0.00	0.00	1.06
SE/GoA	FAIRHOPE	AL	0.75	1.66	1.99	2.86	2.31	2.32	0.00	0.00	1.92
SE/GoA	FOLEY	AL	1.00	1.87	1.22	1.82	1.00	1.00	0.00	0.00	1.50
SE/GoA	GRAND BAY	AL	1.00	1.60	1.66	2.09	1.00	1.00	0.00	0.00	1.42
SE/GoA	GULF SHORES	AL	1.00	1.82	1.39	1.77	1.12	1.12	0.00	0.00	1.50
SE/GoA	IRVINGTON	AL	0.99	1.90	1.28	2.10	1.26	1.26	0.00	0.01	3.00
SE/GoA	LILLIAN	AL	0.96	1.49	1.84	2.19	1.00	1.00	0.00	0.00	1.14
SE/GoA	LOXLEY	AL	1.00	1.88	1.20	1.80	1.00	1.00	0.00	0.00	1.00
SE/GoA	MOBILE	AL	0.98	1.73	1.61	1.86	1.01	1.02	0.00	0.00	2.65

SE/GoA	MONROEVILLE	AL	1.00	1.48	1.84	2.20	1.00	1.00	0.00	0.00	1.00
SE/GoA	ORANGE BEACH	AL	1.00	1.52	1.79	2.14	1.02	1.02	0.00	0.00	1.25
SE/GoA	ROBERTSDALE	AL	1.00	1.48	1.84	2.20	1.00	1.00	0.00	0.00	1.00
SE/GoA	SHORTER	AL	1.00	1.98	1.15	1.88	1.00	1.00	0.00	0.00	1.86
SE/GoA	SPANISH FORT	AL	1.00	1.88	1.20	1.80	1.00	1.00	0.00	0.00	1.00
SE/GoA	STAPLETON	AL	0.10	1.52	1.96	2.20	1.00	1.00	0.00	0.00	1.11
SE/GoA	SUMMERDALE	AL	1.00	1.80	1.33	1.88	1.00	1.00	0.00	0.00	1.48
SE/GoA	THEODORE	AL	1.00	1.79	1.57	1.74	1.19	1.19	0.00	0.00	1.89
SE/GoA	TUSCUMBIA	AL	1.00	1.88	1.20	1.80	1.00	1.00	0.00	0.00	1.00
SE/GoA	ALVA	FL	0.97	1.80	1.96	1.57	1.00	1.00	0.00	0.00	1.09
SE/GoA	ANNA MARIA	FL	0.96	1.75	2.05	2.08	1.19	1.22	0.00	0.00	3.15
SE/GoA	APALACHICOLA	FL	0.98	1.69	2.44	1.77	1.93	1.93	0.00	0.00	4.56
SE/GoA	APOLLO BEACH	FL	0.53	1.66	1.91	1.84	1.00	1.00	0.00	0.00	1.57
SE/GoA	ARIPEKA	FL	0.99	1.84	1.92	2.36	1.00	1.00	0.00	0.00	1.03
SE/GoA	BAYSHORE GARDENS	FL	1.00	1.48	1.84	2.20	1.00	1.00	0.00	0.00	1.00
SE/GoA	BELLEVIEW	FL	0.97	1.48	1.84	2.20	1.00	1.00	0.00	0.00	1.08
SE/GoA	BLOUNTSTOWN	FL	1.00	1.83	1.25	1.82	1.00	1.00	0.00	0.00	1.61
SE/GoA	BOCA GRANDE	FL	0.68	1.79	1.91	2.31	1.00	1.00	0.00	0.00	1.87
SE/GoA	BOKEELIA	FL	0.26	1.78	1.69	1.78	1.08	1.09	0.01	0.00	2.01
SE/GoA	BONITA SPRINGS	FL	1.00	1.52	1.85	2.22	1.00	1.00	0.00	0.00	1.25
SE/GoA	BRADENTON	FL	0.86	1.71	1.93	2.01	1.03	1.04	0.00	0.00	2.43
SE/GoA	BRADENTON BEACH	FL	1.00	1.84	1.92	2.36	1.00	1.00	0.00	0.00	1.00
SE/GoA	BRANDON	FL	0.85	1.66	2.13	1.99	1.50	1.50	0.00	0.00	2.97
SE/GoA	CALLAWAY	FL	0.98	1.51	1.88	2.09	1.00	1.00	0.00	0.00	1.57
SE/GoA	CANTONMENT	FL	0.99	1.75	1.94	1.66	1.00	1.00	0.00	0.00	1.20
SE/GoA	CAPE CORAL	FL	0.69	1.54	1.88	2.20	1.03	1.03	0.00	0.00	1.81
SE/GoA	CAPTIVA	FL	1.00	1.84	1.92	2.36	1.00	1.00	0.00	0.00	1.00
SE/GoA	CARRABELLE	FL	0.77	1.97	1.20	1.98	1.06	1.06	0.00	0.00	1.85
SE/GoA	CEDAR KEY	FL	0.86	1.61	1.87	3.11	2.24	2.24	0.00	0.00	2.41
SE/GoA	CHIEFLAND	FL	1.00	1.61	1.87	3.06	2.15	2.15	0.00	0.00	2.06
SE/GoA	CHOKOLOSKEE	FL	1.00	1.79	1.93	2.38	1.11	1.11	0.00	0.00	1.27
SE/GoA	CLEARWATER	FL	0.55	1.83	2.32	2.13	1.52	1.52	0.00	0.00	3.25
SE/GoA	CORTEZ	FL	0.74	1.89	2.43	1.94	1.68	1.74	0.00	0.01	8.28
SE/GoA	CRAWFORDVILLE	FL	0.89	1.58	1.87	2.22	1.04	1.04	0.00	0.00	2.44
SE/GoA	CROSS CITY	FL	0.99	1.72	2.03	2.43	1.47	1.47	0.00	0.00	2.99
SE/GoA	CRYSTAL RIVER	FL	0.98	1.77	1.99	2.27	1.13	1.13	0.00	0.00	2.04
SE/GoA	DE FUNIAK SPRINGS	FL	1.00	1.79	1.82	1.63	1.00	1.00	0.00	0.00	1.45
SE/GoA	DESTIN	FL	0.98	1.51	2.02	1.53	1.40	1.83	0.00	0.01	3.18
SE/GoA	DOVER	FL	0.97	1.62	1.89	1.94	1.00	1.00	0.00	0.00	2.13
SE/GoA	DUNEDIN	FL	0.98	1.70	2.07	2.17	1.23	1.23	0.00	0.00	2.24
SE/GoA	DUNNELLON	FL	1.00	1.70	1.56	2.11	1.03	1.03	0.00	0.00	1.05
SE/GoA	EAGLE LAKE	FL	0.93	1.79	1.96	1.58	1.00	1.01	0.00	0.00	1.26
SE/GoA	EASTPOINT	FL	0.86	1.64	1.94	2.39	1.66	1.66	0.00	0.00	4.87
SE/GoA	ENGLEWOOD	FL	0.88	1.79	1.92	2.11	1.00	1.00	0.00	0.00	2.11
SE/GoA	ESTERO	FL	0.99	1.83	1.94	2.33	1.03	1.03	0.00	0.00	1.22
SE/GoA	EVERGLADES	FL	1.00	1.84	1.92	2.36	1.00	1.00	0.00	0.00	1.02
SE/GoA	FORT MYERS	FL	0.99	1.62	1.98	2.18	1.15	1.16	0.00	0.00	2.21
SE/GoA	FORT MYERS BEACH	FL	0.99	1.76	1.39	1.84	1.04	1.05	0.00	0.02	1.13
SE/GoA	FORT WALTON BEACH	FL	0.87	1.54	2.21	1.80	1.41	1.42	0.00	0.00	4.49

SE/GoA	FOUNTAIN	FL	0.96	1.98	1.15	1.87	1.00	1.00	0.00	0.00	1.94
SE/GoA	FREEPORT	FL	0.97	1.69	1.68	1.98	1.00	1.00	0.00	0.00	3.09
SE/GoA	GIBSONTON	FL	0.25	1.48	1.88	2.04	1.00	1.00	0.00	0.00	1.59
SE/GoA	GOODLAND	FL	0.95	1.68	2.22	2.01	1.44	1.46	0.00	0.00	3.14
SE/GoA	GULF BREEZE	FL	0.32	1.72	2.13	1.58	1.22	1.22	0.00	0.00	1.67
SE/GoA	GULFPORT	FL	1.00	1.74	1.94	1.67	1.00	1.00	0.00	0.00	1.42
SE/GoA	HERNANDO BEACH	FL	0.44	1.73	1.74	2.18	1.00	1.00	0.01	0.00	3.19
SE/GoA	HOLMES BEACH	FL	0.46	1.59	2.48	1.75	1.74	1.85	0.00	0.00	2.98
SE/GoA	HOMOSASSA	FL	0.67	1.81	1.98	2.28	1.16	1.16	0.00	0.00	2.48
SE/GoA	HORSESHOE BEACH	FL	0.36	1.89	1.79	2.46	1.40	1.40	0.00	0.00	3.18
SE/GoA	HUDSON	FL	0.34	1.82	1.95	2.31	1.08	1.08	0.00	0.00	1.91
SE/GoA	INDIAN ROCKS BEACH	FL	1.00	1.81	2.15	2.17	1.31	1.31	0.00	0.00	2.24
SE/GoA	INGLIS	FL	0.81	1.54	1.77	2.17	1.00	1.00	0.00	0.00	1.81
SE/GoA	INVERNESS	FL	1.00	1.69	1.89	2.29	1.00	1.00	0.00	0.00	1.73
SE/GoA	LAND O LAKES	FL	0.99	1.81	1.95	2.23	1.08	1.16	0.00	0.00	1.61
SE/GoA	LARGO	FL	1.00	1.83	2.76	1.87	2.12	2.14	0.00	0.00	3.57
SE/GoA	LECANTO	FL	1.00	1.84	2.26	2.18	1.44	1.44	0.00	0.00	1.73
SE/GoA	LEESBURG	FL	0.99	1.61	2.00	2.01	1.24	1.25	0.00	0.00	2.26
SE/GoA	LYNN HAVEN	FL	1.00	1.48	1.84	2.20	1.00	1.00	0.00	0.00	1.01
SE/GoA	MADEIRA BEACH	FL	0.95	1.88	2.79	1.88	2.15	2.17	0.00	0.01	3.69
SE/GoA	MARCO ISLAND	FL	0.92	1.86	2.04	1.95	1.45	1.51	0.00	0.00	6.06
SE/GoA	MATLACHA	FL	0.91	1.79	2.50	1.86	1.80	1.82	0.00	0.00	4.88
SE/GoA	MAYO	FL	1.00	1.71	1.93	1.74	1.00	1.00	0.00	0.00	1.13
SE/GoA	MEXICO BEACH	FL	0.04	1.81	1.95	1.64	1.00	1.00	0.00	0.00	1.16
SE/GoA	MILTON	FL	0.85	1.71	1.95	1.71	1.01	1.01	0.00	0.00	2.56
SE/GoA	NAPLES	FL	0.78	1.78	1.94	2.17	1.10	1.28	0.00	0.01	2.66
SE/GoA	NAVARRE	FL	0.99	1.79	1.95	1.60	1.00	1.00	0.00	0.00	1.20
SE/GoA	NEW PORT RICHEY	FL	0.28	1.48	1.88	2.04	1.00	1.00	0.00	0.00	1.69
SE/GoA	NICEVILLE	FL	0.47	1.95	1.31	1.89	1.00	1.00	0.00	0.00	3.22
SE/GoA	NOKOMIS	FL	1.00	1.87	2.21	2.26	1.30	1.30	0.00	0.00	1.58
SE/GoA	NORTH FORT MYERS	FL	0.95	1.51	1.90	2.17	1.07	1.08	0.00	0.00	1.61
SE/GoA	NORTH PORT	FL	0.06	1.48	1.88	2.11	1.14	1.14	0.00	0.00	2.12
SE/GoA	ODESSA	FL	1.00	1.74	2.01	2.22	1.13	1.13	0.00	0.00	1.44
SE/GoA	OSPREY	FL	1.00	1.84	1.92	2.36	1.00	1.00	0.00	0.00	1.00
SE/GoA	PALM HARBOR	FL	0.77	1.49	1.88	2.05	1.00	1.00	0.00	0.00	1.87
SE/GoA	PALMETTO	FL	0.76	1.55	1.88	2.07	1.00	1.00	0.00	0.00	1.97
SE/GoA	PANACEA	FL	0.95	1.67	2.20	2.00	1.61	1.61	0.00	0.00	5.52
SE/GoA	PANAMA CITY	FL	0.85	1.71	2.52	1.65	2.00	2.01	0.00	0.01	5.22
SE/GoA	PANAMA CITY BEACH	FL	0.31	1.60	1.87	3.04	2.19	2.19	0.00	0.00	3.92
SE/GoA	PENSACOLA	FL	0.97	1.61	1.96	1.57	1.31	1.31	0.00	0.00	4.09
SE/GoA	PERRY	FL	0.74	1.62	1.88	2.14	1.05	1.05	0.00	0.00	3.27
SE/GoA	PINELAND	FL	0.68	1.78	1.91	1.57	1.00	1.00	0.00	0.00	1.77
SE/GoA	PINELLAS PARK	FL	0.65	1.85	1.77	1.83	1.03	1.03	0.00	0.00	3.70
SE/GoA	PLACIDA	FL	1.00	1.76	2.07	2.23	1.19	1.19	0.00	0.00	1.84
SE/GoA	PORT CHARLOTTE	FL	0.36	1.64	1.88	2.20	1.00	1.00	0.00	0.00	1.96
SE/GoA	PORT RICHEY	FL	0.05	1.53	2.11	1.94	1.33	1.33	0.00	0.00	1.08
SE/GoA	PORT SAINT JOE	FL	0.67	1.86	1.44	1.78	1.00	1.00	0.00	0.00	6.22
SE/GoA	PUNTA GORDA	FL	0.97	1.57	1.87	2.06	1.00	1.00	0.00	0.00	1.90
SE/GoA	REDINGTON SHORES	FL	0.98	1.85	2.09	2.10	1.24	1.26	0.00	0.00	2.69

SE/GoA	ROTONDA	FL	1.00	1.84	1.92	2.36	1.00	1.00	0.00	0.00	1.00
SE/GoA	RUBONIA	FL	0.01	1.88	1.88	1.92	1.00	1.00	0.00	0.00	1.01
SE/GoA	RUSKIN	FL	0.32	1.54	1.85	2.19	1.00	1.00	0.00	0.00	2.03
SE/GoA	SAFETY HARBOR	FL	1.00	1.48	1.84	2.20	1.00	1.00	0.00	0.00	1.00
SE/GoA	SAINT JAMES CITY	FL	0.96	1.67	1.90	2.20	1.01	1.01	0.00	0.00	2.30
SE/GoA	SAINT MARKS	FL	0.94	1.77	2.07	2.16	1.20	1.20	0.00	0.00	2.65
SE/GoA	SAINT PETERSBURG	FL	0.96	1.81	2.58	1.91	1.91	1.92	0.00	0.01	4.82
SE/GoA	SAINT PETERSBURG BEACH	FL	0.96	1.80	1.93	2.33	1.04	1.04	0.00	0.00	1.41
SE/GoA	SANIBEL	FL	0.28	1.58	1.87	1.87	1.00	1.00	0.00	0.00	3.01
SE/GoA	SANTA ROSA BEACH	FL	0.92	1.58	1.93	1.92	1.12	1.25	0.00	0.00	2.87
SE/GoA	SARASOTA	FL	0.99	1.80	1.91	2.30	1.03	1.03	0.00	0.00	1.72
SE/GoA	SEMINOLE	FL	0.99	1.97	2.84	1.98	2.14	2.16	0.00	0.00	2.09
SE/GoA	SOPCHOPPY	FL	0.99	1.50	1.84	2.16	1.00	1.00	0.00	0.00	1.16
SE/GoA	SOUTHPORT	FL	0.87	1.84	1.40	2.03	1.05	1.05	0.00	0.00	2.63
SE/GoA	SPRING HILL	FL	0.00	1.72	2.27	1.91	1.71	1.73	0.01	0.00	1.48
SE/GoA	ST. GEORGE ISLAND	FL	0.99	1.86	1.22	1.81	1.00	1.00	0.00	0.00	1.30
SE/GoA	STEINHATCHEE	FL	0.93	1.61	2.25	1.93	1.46	1.46	0.00	0.00	4.91
SE/GoA	SUWANNEE	FL	1.00	1.68	1.88	3.18	2.27	2.27	0.00	0.00	2.12
SE/GoA	TALLAHASSEE	FL	0.98	1.60	2.22	1.91	1.47	1.47	0.00	0.00	3.80
SE/GoA	TAMPA	FL	0.96	1.75	1.37	1.84	1.01	1.01	0.00	0.01	1.27
SE/GoA	TARPON SPRINGS	FL	0.83	1.80	2.15	1.96	1.46	1.49	0.00	0.00	7.38
SE/GoA	TERRA CEIA	FL	1.00	1.84	1.92	2.36	1.00	1.00	0.00	0.00	1.00
SE/GoA	UNKNOWN	FL	1.00	2.04	1.12	1.92	1.00	1.00	0.00	0.00	1.00
SE/GoA	VALPARAISO	FL	1.00	1.99	1.15	1.88	1.00	1.00	0.00	0.00	1.75
SE/GoA	VENICE	FL	1.00	1.82	2.54	2.05	1.69	1.69	0.00	0.00	2.62
SE/GoA	WEEKI WACHEE	FL	0.97	1.84	1.94	2.34	1.03	1.03	0.00	0.00	1.15
SE/GoA	WEWAHITCHKA	FL	1.00	1.85	1.23	1.82	1.00	1.00	0.00	0.00	1.37
SE/GoA	YANKEETOWN	FL	0.72	1.57	1.71	1.98	1.00	1.00	0.00	0.00	1.79
SE/GoA	YOUNGSTOWN	FL	0.75	1.68	1.87	3.66	2.98	2.98	0.00	0.00	1.05
SE/GoA	ABBEVILLE	LA	0.56	1.87	1.22	1.82	1.00	1.00	0.24	0.03	2.11
SE/GoA	ABITA SPRINGS	LA	1.00	1.88	1.20	1.80	1.00	1.00	0.00	0.00	1.00
SE/GoA	AKERS	LA	1.00	1.48	1.84	2.20	1.00	1.00	0.00	0.00	1.00
SE/GoA	ALBANY	LA	1.00	1.56	1.72	2.12	1.00	1.00	0.00	0.00	1.32
SE/GoA	AMA	LA	1.00	1.88	1.20	1.80	1.00	1.00	0.00	0.00	1.00
SE/GoA	AMELIA	LA	1.00	1.75	1.39	1.91	1.00	1.00	0.00	0.00	1.01
SE/GoA	AMITE CITY	LA	1.00	1.88	1.20	1.80	1.00	1.00	0.00	0.00	1.00
SE/GoA	ANGIE	LA	1.00	1.50	1.81	2.18	1.00	1.00	0.00	0.00	1.12
SE/GoA	ARABI	LA	1.00	1.88	1.20	1.80	1.00	1.00	0.00	0.00	1.00
SE/GoA	ARNAUDVILLE	LA	1.00	1.55	1.73	2.13	1.00	1.00	0.00	0.00	1.41
SE/GoA	BAKER	LA	1.00	2.00	1.14	1.89	1.00	1.00	0.00	0.00	1.64
SE/GoA	BALDWIN	LA	1.00	1.78	1.36	1.90	1.00	1.00	0.00	0.00	1.01
SE/GoA	BARATARIA	LA	1.00	1.78	1.38	1.95	1.05	1.05	0.00	0.00	1.64
SE/GoA	BASILE	LA	1.00	1.87	1.25	1.87	1.00	1.00	0.00	0.00	1.93
SE/GoA	BASTROP	LA	1.00	1.48	1.84	2.20	1.00	1.00	0.00	0.00	1.00
SE/GoA	BATON ROUGE	LA	1.00	1.71	1.80	2.42	1.37	1.37	0.00	0.00	1.73
SE/GoA	BAYOU L'OURSE	LA	1.00	2.03	1.12	1.91	1.00	1.00	0.00	0.00	1.14
SE/GoA	BELL CITY	LA	1.00	1.52	1.78	2.16	1.00	1.00	0.00	0.00	1.21
SE/GoA	BELLE CHASSE	LA	1.00	1.84	1.41	2.43	1.65	1.65	0.01	0.02	2.38
SE/GoA	BERWICK	LA	1.00	1.48	1.83	2.20	1.00	1.00	0.00	0.00	1.02

SE/GoA	BOGALUSA	LA	1.00	1.89	1.25	1.89	1.00	1.00	0.00	0.00	2.22
SE/GoA	BOOTHVILLE	LA	1.00	1.90	1.19	1.81	1.00	1.00	0.00	0.01	1.24
SE/GoA	BOSSIER CITY	LA	1.00	1.48	1.84	2.20	1.00	1.00	0.00	0.00	1.00
SE/GoA	BOURG	LA	1.00	1.52	1.85	2.46	1.34	1.34	0.00	0.00	1.44
SE/GoA	BOUTTE	LA	1.00	1.48	1.84	2.20	1.00	1.00	0.00	0.00	1.00
SE/GoA	BRAITHWAITE	LA	1.00	1.68	1.87	3.67	2.99	2.99	0.00	0.01	1.02
SE/GoA	BREAUX BRIDGE	LA	0.28	1.55	1.74	2.15	1.00	1.00	0.00	0.00	1.46
SE/GoA	BRITTANY	LA	1.00	1.48	1.84	2.20	1.00	1.00	0.00	0.00	1.00
SE/GoA	BROUSSARD	LA	1.00	1.92	1.18	1.83	1.00	1.00	0.00	0.00	1.49
SE/GoA	BURAS	LA	1.00	1.84	1.36	2.21	1.40	1.40	0.01	0.01	2.24
SE/GoA	BUSH	LA	1.00	1.51	1.85	2.41	1.28	1.28	0.00	0.00	1.25
SE/GoA	CALHOUN	LA	1.00	1.88	1.20	1.80	1.00	1.00	0.00	0.00	1.00
SE/GoA	CAMERON	LA	0.96	1.57	2.20	2.05	1.93	1.94	0.00	0.00	2.85
SE/GoA	CARENCRO	LA	1.00	2.04	1.12	1.92	1.00	1.00	0.00	0.00	1.00
SE/GoA	CENTERVILLE	LA	1.00	1.48	1.84	2.20	1.00	1.00	0.00	0.00	1.00
SE/GoA	CHALMETTE	LA	1.00	1.58	1.69	2.11	1.00	1.00	0.00	0.00	1.63
SE/GoA	CHARENTON	LA	1.00	1.48	1.84	2.20	1.00	1.00	0.00	0.00	1.00
SE/GoA	CHAUVIN	LA	0.99	1.80	1.35	1.94	1.03	1.03	0.01	0.02	2.26
SE/GoA	CHURCH POINT	LA	0.41	1.48	1.84	2.20	1.00	1.00	0.00	0.00	1.25
SE/GoA	CLINTON	LA	1.00	2.04	1.12	1.92	1.00	1.00	0.00	0.00	1.00
SE/GoA	COCODRIE	LA	1.00	1.68	1.88	3.68	3.00	3.00	0.00	0.00	1.00
SE/GoA	CONVENT	LA	1.00	1.48	1.84	2.20	1.00	1.00	0.00	0.00	1.00
SE/GoA	COVINGTON	LA	1.00	1.68	1.88	3.68	3.00	3.00	0.00	0.00	1.00
SE/GoA	CREOLE	LA	1.00	1.61	1.89	1.95	1.00	1.00	0.00	0.00	1.87
SE/GoA	CROWLEY	LA	1.00	1.64	1.58	2.04	1.00	1.00	0.00	0.00	1.91
SE/GoA	CUT OFF	LA	1.00	1.84	1.31	1.92	1.01	1.01	0.00	0.00	2.02
SE/GoA	CYPREMORT POINT	LA	1.00	1.48	1.84	2.20	1.00	1.00	0.00	0.00	1.00
SE/GoA	DELACROIX	LA	1.00	1.90	1.19	1.82	1.01	1.01	0.00	0.00	1.26
SE/GoA	DELCAMBRE	LA	1.00	1.85	1.25	1.83	1.00	1.00	0.00	0.00	1.19
SE/GoA	DENHAM SPRINGS	LA	1.00	1.48	1.84	2.20	1.00	1.00	0.00	0.00	1.00
SE/GoA	DES ALLEMANDS	LA	1.00	1.48	1.84	2.20	1.00	1.00	0.00	0.01	1.01
SE/GoA	DESTREHAN	LA	1.00	1.55	1.74	2.14	1.00	1.00	0.00	0.00	1.42
SE/GoA	DULAC	LA	0.82	1.87	1.26	1.87	1.01	1.01	0.00	0.01	2.88
SE/GoA	EMPIRE	LA	0.00	2.04	1.12	1.92	1.00	1.00	0.34	0.09	1.00
SE/GoA	ERATH	LA	1.00	1.72	1.45	1.96	1.00	1.00	0.00	0.00	1.60
SE/GoA	ETHEL	LA	1.00	1.48	1.84	2.20	1.00	1.00	0.00	0.00	1.00
SE/GoA	EUNICE	LA	0.10	1.90	1.21	1.83	1.00	1.00	0.00	0.00	1.95
SE/GoA	EVANGELINE	LA	1.00	1.91	1.18	1.83	1.00	1.00	0.00	0.00	1.29
SE/GoA	FERRIDAY	LA	1.00	2.04	1.12	1.92	1.00	1.00	0.00	0.00	1.00
SE/GoA	FOLSOM	LA	1.00	1.48	1.84	2.20	1.00	1.00	0.00	0.00	1.00
SE/GoA	FRANKLIN	LA	1.00	1.54	1.82	2.18	1.00	1.00	0.00	0.00	1.73
SE/GoA	FRANKLINTON	LA	1.00	1.88	1.20	1.80	1.00	1.00	0.00	0.00	1.01
SE/GoA	GALLIANO	LA	0.91	1.77	1.73	1.73	1.28	1.28	0.00	0.00	1.90
SE/GoA	GHEENS	LA	1.00	1.48	1.84	2.20	1.00	1.00	0.00	0.00	1.00
SE/GoA	GIBSON	LA	1.00	1.48	1.84	2.20	1.00	1.00	0.00	0.00	1.00
SE/GoA	GOLDEN MEADOW	LA	0.99	1.85	1.44	1.90	1.17	1.18	0.01	0.02	2.71
SE/GoA	GONZALES	LA	1.00	1.48	1.84	2.20	1.00	1.00	0.00	0.00	1.00
SE/GoA	GRAMERCY	LA	1.00	1.76	1.48	2.06	1.00	1.00	0.00	0.00	1.05
SE/GoA	GRAND CHENIER	LA	1.00	1.48	1.84	2.20	1.00	1.00	0.00	0.00	1.01
SE/GoA	GRAND ISLE	LA	1.00	1.90	1.22	1.85	1.02	1.04	0.01	0.01	1.89
SE/GoA	GRAY	LA	1.00	1.85	1.27	1.86	1.00	1.00	0.00	0.00	1.59
SE/GoA	GRETNA	LA	0.85	1.56	1.82	2.09	1.00	1.00	0.00	0.00	2.34
SE/GoA	GUEYDAN	LA	1.00	1.78	1.36	1.90	1.00	1.00	0.00	0.00	1.59

SE/GoA	HACKBERRY	LA	0.99	1.50	1.89	2.23	1.01	1.01	0.00	0.00	1.35
SE/GoA	HAMMOND	LA	0.99	1.48	1.84	2.20	1.00	1.00	0.00	0.00	1.03
SE/GoA	HARAHAN	LA	1.00	1.68	1.88	3.68	3.00	3.00	0.00	0.00	1.00
SE/GoA	HARVEY	LA	1.00	1.72	1.76	3.35	2.63	2.63	0.00	0.00	1.40
SE/GoA	HENDERSON	LA	1.00	1.88	1.20	1.80	1.00	1.00	0.00	0.00	1.13
SE/GoA	HOUMA	LA	0.92	1.65	1.96	2.53	2.06	2.06	0.00	0.02	4.42
SE/GoA	IOWA	LA	1.00	1.48	1.84	2.20	1.00	1.00	0.00	0.00	1.00
SE/GoA	JACKSON	LA	1.00	1.64	1.61	2.08	1.00	1.00	0.00	0.00	1.15
SE/GoA	JEANERETTE	LA	1.00	1.73	1.45	1.96	1.00	1.00	0.00	0.00	1.51
SE/GoA	JEFFERSON	LA	1.00	1.56	1.71	2.12	1.00	1.00	0.00	0.00	1.04
SE/GoA	JENNINGS	LA	1.00	1.73	1.45	1.96	1.00	1.00	0.00	0.00	1.75
SE/GoA	JONESVILLE	LA	1.00	1.48	1.88	2.04	1.00	1.00	0.00	0.00	1.00
SE/GoA	KAPLAN	LA	1.00	1.50	1.82	2.18	1.00	1.00	0.00	0.00	1.08
SE/GoA	KENNER	LA	1.00	1.57	2.12	2.34	1.05	1.05	0.00	0.00	1.96
SE/GoA	KENTWOOD	LA	1.00	1.48	1.84	2.20	1.00	1.00	0.00	0.00	1.00
SE/GoA	KINDER	LA	1.00	1.88	1.20	1.80	1.00	1.00	0.00	0.00	1.00
SE/GoA	LACOMBE	LA	1.00	1.48	1.84	2.20	1.00	1.00	0.00	0.00	1.00
SE/GoA	LAFAYETTE	LA	1.00	1.50	1.81	2.18	1.00	1.00	0.00	0.00	1.12
SE/GoA	LAFITTE	LA	1.00	1.80	1.36	1.93	1.00	1.00	0.00	0.01	2.18
SE/GoA	LAKE ARTHUR	LA	1.00	1.63	1.60	2.05	1.00	1.00	0.00	0.00	1.38
SE/GoA	LAKE CHARLES	LA	1.00	1.59	1.68	2.10	1.00	1.00	0.00	0.00	1.52
SE/GoA	LAPLACE	LA	1.00	1.55	1.72	2.13	1.00	1.00	0.00	0.00	1.04
SE/GoA	LAROSE	LA	1.00	1.48	1.84	2.20	1.00	1.00	0.00	0.01	1.00
SE/GoA	LEESVILLE	LA	1.00	2.04	1.12	1.92	1.00	1.00	0.00	0.00	1.00
SE/GoA	LIVINGSTON	LA	1.00	1.68	1.52	2.00	1.00	1.00	0.00	0.00	1.00
SE/GoA	LIVONIA	LA	0.71	1.61	1.63	2.07	1.00	1.00	0.00	0.00	1.13
SE/GoA	LOCKPORT	LA	1.00	1.87	1.25	1.87	1.00	1.00	0.00	0.00	1.82
SE/GoA	LORANGER	LA	1.00	1.77	1.43	1.99	1.00	1.00	0.00	0.00	1.13
SE/GoA	LOREAUVILLE	LA	0.49	1.60	2.24	2.40	1.00	1.00	0.00	0.00	1.63
SE/GoA	LULING	LA	1.00	1.51	1.79	2.17	1.00	1.00	0.00	0.00	1.18
SE/GoA	LYDIA	LA	1.00	1.56	1.86	2.76	1.75	1.75	0.00	0.00	1.79
SE/GoA	MADISONVILLE	LA	1.00	1.48	1.84	2.20	1.00	1.00	0.00	0.00	1.01
SE/GoA	MANDEVILLE	LA	1.00	1.79	1.37	1.92	1.00	1.00	0.00	0.00	1.46
SE/GoA	MANSURA	LA	0.92	1.88	1.20	1.80	1.00	1.00	0.00	0.00	1.54
SE/GoA	MARKSVILLE	LA	1.00	1.48	1.84	2.20	1.00	1.00	0.00	0.00	1.00
SE/GoA	MARRERO	LA	1.00	1.56	1.72	2.13	1.00	1.00	0.00	0.00	1.45
SE/GoA	MAUREPAS	LA	1.00	1.48	1.84	2.20	1.00	1.00		0.00	1.00
SE/GoA	MAURICE	LA	1.00	1.84	1.28	1.87	1.00	1.00	0.00	0.00	1.66
SE/GoA	MERAUX	LA	1.00	1.54	1.77	2.16	1.00	1.00	0.00	0.00	1.49
SE/GoA	METAIRIE	LA	1.00	1.48	1.84	2.20	1.00	1.00	0.00	0.00	1.02
SE/GoA	MONTEGUT	LA	1.00	1.78	1.53	2.50	1.71	1.71	0.00	0.01	3.30
SE/GoA	MORGAN CITY	LA	0.84	1.78	1.40	1.95	1.00	1.00	0.00	0.00	2.93
SE/GoA	NAPOLEONVILLE	LA	1.00	1.48	1.84	2.20	1.00	1.00	0.00	0.00	1.00
SE/GoA	NEW IBERIA	LA	1.00	1.48	1.84	2.20	1.00	1.00	0.00	0.01	1.01
SE/GoA	NEW ORLEANS	LA	0.98	1.53	1.87	2.32	1.16	1.16	0.00	0.01	2.09
SE/GoA	NORCO	LA	1.00	1.56	1.81	2.62	1.59	1.59	0.00	0.00	1.28
SE/GoA	OPELOUSAS	LA	0.38	1.75	1.41	1.93	1.00	1.00	0.00	0.00	1.30
SE/GoA	PARADIS	LA	1.00	1.48	1.84	2.20	1.00	1.00	0.00	0.00	1.00
SE/GoA	PATTERSON	LA	1.00	1.54	1.75	2.15	1.00	1.00	0.00	0.00	1.34
SE/GoA	PAULINA	LA	1.00	1.82	1.37	1.98	1.00	1.00	0.00	0.00	2.27
SE/GoA	PEARL RIVER	LA	1.00	1.55	1.85	2.69	1.66	1.66	0.00	0.00	1.52
SE/GoA	PERRY	LA	1.00	1.73	1.71	3.20	2.49	2.49	0.00	0.00	1.12
SE/GoA	PIERRE PART	LA	0.83	1.51	1.79	2.17	1.01	1.01	0.00	0.00	1.65

SE/GoA	PLAQUEMINE	LA	0.04	1.64	1.55	1.97	1.00	1.09	0.00	0.00	1.50
SE/GoA	POINTE A LA HACHE	LA	1.00	1.48	1.84	2.20	1.00	1.00	0.00	0.00	1.00
SE/GoA	PONCHATOULA	LA	0.95	1.56	1.72	2.13	1.00	1.00	0.00	0.00	1.38
SE/GoA	PORT ALLEN	LA	1.00	1.51	1.79	2.17	1.00	1.00	0.00	0.00	1.17
SE/GoA	PORT SULPHUR	LA	1.00	1.70	1.81	3.51	2.81	2.81	0.00	0.02	1.21
SE/GoA	PRAIRIEVILLE	LA	1.00	1.62	1.62	2.06	1.00	1.00	0.00	0.00	1.29
SE/GoA	RACELAND	LA	1.00	1.48	1.84	2.20	1.00	1.00	0.00	0.00	1.00
SE/GoA	RAGLEY	LA	1.00	1.88	1.20	1.80	1.00	1.00	0.00	0.00	1.00
SE/GoA	RAYNE	LA	1.00	1.48	1.84	2.20	1.00	1.00	0.00	0.00	1.00
SE/GoA	RESERVE	LA	0.44	1.61	1.63	2.08	1.00	1.00	0.00	0.00	2.79
SE/GoA	RIVER RIDGE	LA	1.00	1.55	2.08	2.33	1.00	1.00	0.00	0.00	1.41
SE/GoA	ROBERT	LA	1.00	1.48	1.84	2.20	1.00	1.00	0.00	0.00	1.00
SE/GoA	SAINT AMANT	LA	1.00	1.48	1.84	2.20	1.00	1.00	0.00	0.00	1.00
SE/GoA	SAINT BERNARD	LA	1.00	1.64	1.74	2.76	1.85	1.85	0.01	0.04	2.72
SE/GoA	SAINT FRANCISVILLE	LA	1.00	1.48	1.84	2.20	1.00	1.00	0.00	0.00	1.00
SE/GoA	SAINT LANDRY	LA	1.00	1.88	1.20	1.80	1.00	1.00	0.00	0.00	1.00
SE/GoA	SAINT MARTINVILLE	LA	0.28	1.74	1.44	1.92	1.00	1.00	0.00	0.00	1.69
SE/GoA	SCHRIEVER	LA	0.34	1.68	1.64	2.56	1.67	1.67	0.00	0.00	1.24
SE/GoA	SIMMESPORT	LA	0.00	1.64	1.91	2.13	1.26	1.26	0.00	0.00	1.03
SE/GoA	SLAUGHTER	LA	1.00	2.00	1.14	1.89	1.00	1.00	0.00	0.00	1.50
SE/GoA	SLIDELL	LA	1.00	1.52	1.82	2.13	1.05	1.05	0.00	0.01	1.35
SE/GoA	STONEWALL	LA	1.00	1.91	1.18	1.83	1.00	1.00	0.00	0.00	1.48
SE/GoA	SULPHUR	LA	1.00	1.86	1.27	1.87	1.00	1.00	0.00	0.00	1.78
SE/GoA	SUNSET	LA	1.00	1.88	1.20	1.80	1.00	1.00	0.00	0.00	1.00
SE/GoA	TERRYTOWN	LA	1.00	1.81	1.52	2.76	2.00	2.00	0.00	0.00	1.46
SE/GoA	THERIOT	LA	1.00	1.71	1.50	2.06	1.07	1.07	0.00	0.01	2.52
SE/GoA	THIBODAUX	LA	0.71	1.55	1.79	2.42	1.34	1.34	0.00	0.00	1.84
SE/GoA	TICKFAW	LA	1.00	1.93	1.19	1.87	1.00	1.00	0.00	0.00	1.42
SE/GoA	UNKNOWN	LA	1.00	1.88	1.20	1.80	1.00	1.00	0.00	0.00	1.05
SE/GoA	VACHERIE	LA	0.99	1.48	1.84	2.20	1.00	1.00	0.00	0.00	1.03
SE/GoA	VENICE	LA	0.99	1.86	1.27	1.80	1.02	1.02	0.01	0.01	1.59
SE/GoA	VILLE PLATTE	LA	1.00	2.04	1.12	1.92	1.00	1.00	0.00	0.00	1.00
SE/GoA	VINTON	LA	1.00	1.51	1.81	2.19	1.00	1.00	0.00	0.00	1.11
SE/GoA	VIOLET	LA	1.00	1.53	1.85	2.58	1.51	1.51	0.00	0.01	1.62
SE/GoA	WELSH	LA	1.00	1.48	1.84	2.20	1.00	1.00	0.00	0.00	1.00
SE/GoA	WESTWEGO	LA	1.00	1.59	1.68	2.11	1.00	1.00	0.00	0.00	1.63
SE/GoA	WHITE CASTLE	LA	1.00	1.48	1.84	2.20	1.00	1.00	0.00	0.00	1.00
SE/GoA	YOUNGSVILLE	LA	1.00	1.88	1.21	1.80	1.00	1.00	0.00	0.00	1.03
SE/GoA	ZACHARY	LA	1.00	1.48	1.84	2.20	1.00	1.00	0.00	0.00	1.00
SE/GoA	BAY SAINT LOUIS	MS	1.00	1.68	1.54	2.03	1.00	1.00	0.00	0.00	1.82
SE/GoA	BILOXI	MS	1.00	1.91	1.21	1.86	1.00	1.00	0.01	0.02	2.30
SE/GoA	BOONEVILLE	MS	1.00	1.88	1.20	1.80	1.00	1.00	0.00	0.00	1.00
SE/GoA	CARRIERE	MS	0.95	1.72	1.95	2.10	1.00	1.00	0.00	0.00	1.22
SE/GoA	DIAMONDHEAD	MS	1.00	1.62	1.58	2.02	1.00	1.00	0.00	0.00	1.00
SE/GoA	D'IBERVILLE	MS	1.00	1.94	1.17	1.85	1.00	1.00	0.00	0.00	1.83
SE/GoA	ELLISVILLE	MS	1.00	1.97	1.22	1.96	1.00	1.00	0.00	0.00	1.30
SE/GoA	FLORENCE	MS	1.00	1.99	1.14	1.89	1.00	1.00	0.00	0.00	1.48
SE/GoA	GAUTIER	MS	0.86	1.81	1.92	1.93	1.00	1.00	0.00	0.00	3.29
SE/GoA	GULFPORT	MS	0.97	1.82	1.33	1.91	1.00	1.00	0.00	0.00	1.97
SE/GoA	HATTIESBURG	MS	0.95	1.61	1.80	1.64	1.18	1.18	0.00	0.00	1.50
SE/GoA	HICKORY	MS	0.60	1.86	2.01	1.83	1.00	1.00	0.00	0.00	3.10

SE/GoA	KILN	MS	1.00	1.71	1.50	2.02	1.00	1.00	0.00	0.00	1.20
SE/GoA	LAKESHORE	MS	1.00	1.68	1.88	3.67	2.99	2.99	0.00	0.00	1.01
SE/GoA	LONG BEACH	MS	0.99	1.86	1.26	1.84	1.00	1.00	0.00	0.00	1.49
SE/GoA	LUCEDALE	MS	0.99	1.86	1.42	1.84	1.00	1.00	0.00	0.00	2.14
SE/GoA	LUMBERTON	MS	1.00	1.88	1.20	1.80	1.00	1.00	0.00	0.00	1.01
SE/GoA	MOSS POINT	MS	0.37	1.73	1.53	1.97	1.00	1.00	0.13	0.02	1.81
SE/GoA	OCEAN SPRINGS	MS	0.99	1.85	1.35	1.87	1.00	1.00	0.00	0.00	1.98
SE/GoA	PASCAGOULA	MS	0.84	1.76	1.70	1.82	1.18	1.18	0.00	0.00	4.48
SE/GoA	PASS CHRISTIAN	MS	0.99	1.85	1.36	2.13	1.29	1.29	0.00	0.00	2.57
SE/GoA	PEARLINGTON	MS	1.00	1.48	1.84	2.20	1.00	1.00	0.00	0.00	1.00
SE/GoA	PERKINSTON	MS	1.00	2.04	1.12	1.92	1.00	1.00	0.00	0.00	1.00
SE/GoA	PICAYUNE	MS	1.00	1.73	1.45	1.96	1.00	1.00	0.00	0.00	1.10
SE/GoA	POPLARVILLE	MS	1.00	1.48	1.84	2.20	1.00	1.00	0.00	0.00	1.00
SE/GoA	PURVIS	MS	1.00	1.88	1.20	1.80	1.00	1.00	0.00	0.00	1.00
SE/GoA	SAUCIER	MS	1.00	1.89	1.20	1.81	1.00	1.00	0.00	0.00	1.09
SE/GoA	STARKVILLE	MS	1.00	2.04	1.12	1.92	1.00	1.00	0.00	0.00	1.00
SE/GoA	TUPELO	MS	1.00	1.48	1.84	2.20	1.00	1.00	0.00	0.00	1.00
SE/GoA	TYLERTOWN	MS	1.00	1.88	1.20	1.80	1.00	1.00	0.00	0.00	1.00
SE/GoA	VANCLEAVE	MS	0.91	1.72	1.58	1.95	1.00	1.00	0.00	0.00	2.60
SE/GoA	VICKSBURG	MS	1.00	1.48	1.84	2.20	1.00	1.00	0.00	0.00	1.00
SE/GoA	WAVELAND	MS	1.00	1.75	1.43	1.97	1.01	1.01	0.00	0.00	2.24
SE/GoA	ANAHUAC	TX	0.99	1.62	1.89	3.19	2.32	2.32	0.00	0.00	1.97
SE/GoA	ARANSAS PASS	TX	0.88	1.97	1.19	1.91	1.00	1.00	0.00	0.00	2.04
SE/GoA	ARROYO CITY	TX	1.00	1.48	1.84	2.20	1.00	1.00	0.00	0.00	1.00
SE/GoA	AUSTIN	TX	1.00	1.60	2.24	2.40	1.00	1.00	0.00	0.00	1.00
SE/GoA	AUSTWELL	TX	1.00	1.68	1.88	3.67	2.99	2.99	0.00	0.00	1.01
SE/GoA	BACLIFF	TX	0.99	1.68	1.88	3.68	3.00	3.00	0.00	0.01	1.01
SE/GoA	BAY CITY	TX	0.85	1.91	1.19	1.83	1.00	1.00	0.00	0.00	2.00
SE/GoA	BOLIVAR PENINSULA	TX	1.00	1.90	1.28	1.82	1.07	1.07	0.01	0.02	2.11
SE/GoA	BROWNSVILLE	TX	0.99	2.01	1.15	1.91	1.00	1.00	0.01	0.02	1.32
SE/GoA	CHANNELVIEW	TX	1.00	1.48	1.84	2.20	1.00	1.00	0.00	0.00	1.00
SE/GoA	CORPUS CHRISTI	TX	0.60	1.62	2.09	2.28	1.03	1.03	0.00	0.00	3.04
SE/GoA	DAYTON	TX	1.00	1.92	1.18	1.83	1.00	1.00	0.00	0.00	1.63
SE/GoA	DEER PARK	TX	1.00	1.44	2.20	1.72	1.00	1.00	0.00	0.00	1.00
SE/GoA	DENISON	TX	1.00	1.88	1.20	1.80	1.00	1.00	0.00	0.00	1.00
SE/GoA	DICKINSON	TX	0.95	1.78	1.56	2.77	2.01	2.01	0.00	0.00	2.49
SE/GoA	EDNA	TX	1.00	1.44	2.20	1.72	1.00	1.00	0.00	0.00	1.00
SE/GoA	FREEPORT	TX	0.81	1.46	2.68	1.50	1.94	1.94	0.00	0.00	1.66
SE/GoA	FULTON	TX	0.97	1.64	1.86	3.23	2.40	2.40	0.00	0.00	1.91
SE/GoA	GALVESTON	TX	0.94	1.69	2.00	1.67	1.54	1.54	0.00	0.03	3.03
SE/GoA	GROVES	TX	1.00	1.48	1.84	2.20	1.00	1.00	0.00	0.00	1.00
SE/GoA	HITCHCOCK	TX	0.06	1.52	1.96	2.19	1.00	1.00	0.00	0.00	1.13
SE/GoA	HOUSTON	TX	0.99	1.47	2.63	1.70	2.07	2.07	0.00	0.00	1.39
SE/GoA	INEZ	TX	0.92	1.58	2.18	2.39	1.00	1.00	0.00	0.00	1.59
SE/GoA	INGLESIDE	TX	0.13	1.51	1.92	2.19	1.00	1.00	0.00	0.00	1.85
SE/GoA	KATY	TX	1.00	1.68	1.88	3.68	3.00	3.00	0.00	0.00	1.00
SE/GoA	KEMAH	TX	1.00	1.88	1.20	1.80	1.00	1.00	0.00	0.00	1.00
SE/GoA	LA PORTE	TX	0.00	1.52	1.85	2.12	1.00	1.00	0.00	0.00	1.01
SE/GoA	LAGUNA VISTA	TX	1.00	2.04	1.12	1.92	1.00	1.00	0.00	0.00	1.04
SE/GoA	LIBERTY	TX	1.00	1.56	1.87	2.73	1.70	1.70	0.00	0.00	1.96
SE/GoA	LOS FRESNOS	TX	1.00	2.02	1.14	1.91	1.00	1.00	0.00	0.01	1.22
SE/GoA	LOYOLA BEACH	TX	1.00	1.60	2.24	2.40	1.00	1.00	0.00	0.00	1.00

SE/GoA	MATAGORDA	TX	0.88	1.57	2.28	1.96	1.94	1.94	0.00	0.00	2.86
SE/GoA	MATHIS	TX	0.19	1.52	1.96	2.20	1.00	1.00	0.00	0.00	1.55
SE/GoA	MCALLEN	TX	0.35	1.80	1.96	1.56	1.00	1.00	0.00	0.00	1.83
SE/GoA	MISSOURI CITY	TX	1.00	1.52	1.96	2.20	1.00	1.00		0.00	1.00
SE/GoA	MUNDAY	TX	1.00	2.04	1.12	1.92	1.00	1.00	0.00	0.00	1.00
SE/GoA	NEDERLAND	TX	1.00	1.49	1.82	2.19	1.00	1.00	0.00	0.00	1.07
SE/GoA	OAK ISLAND	TX	1.00	1.48	1.84	2.20	1.00	1.00	0.00	0.00	1.00
SE/GoA	ORANGE	TX	1.00	1.50	1.82	2.18	1.00	1.00	0.00	0.00	1.09
SE/GoA	PALACIOS	TX	1.00	1.99	1.15	1.89	1.00	1.00	0.01	0.05	1.77
SE/GoA	PEARLAND	TX	1.00	1.44	2.20	1.72	1.00	1.00	0.00	0.00	1.00
SE/GoA	PORT ARANSAS	TX	0.14	1.74	1.57	2.01	1.00	1.00	0.00	0.00	1.64
SE/GoA	PORT ARTHUR	TX	1.00	1.90	1.20	1.83	1.00	1.00	0.01	0.05	1.46
SE/GoA	PORT ISABEL	TX	0.96	2.01	1.14	1.91	1.00	1.00	0.00	0.02	1.36
SE/GoA	PORT LAVACA	TX	0.87	1.68	1.85	3.55	2.85	2.85	0.00	0.00	1.53
SE/GoA	PORT MANSFIELD	TX	0.78	1.58	2.15	2.33	1.00	1.00	0.00	0.00	2.64
SE/GoA	PORT O'CONNOR	TX	0.66	1.68	1.76	2.84	1.98	1.98	0.00	0.00	3.27
SE/GoA	PORTLAND	TX	0.27	1.65	1.91	1.86	1.00	1.00	0.00	0.00	2.36
SE/GoA	RAYMONDVILLE	TX	1.00	1.80	1.96	1.56	1.00	1.00	0.00	0.00	1.00
SE/GoA	RIO HONDO	TX	0.67	1.48	1.84	2.20	1.00	1.00	0.00	0.00	1.62
SE/GoA	RIVIERA	TX	0.97	1.65	1.62	2.01	1.00	1.00	0.00	0.00	1.34
SE/GoA	ROCKPORT	TX	0.43	1.62	1.79	2.08	1.00	1.00	0.00	0.00	2.48
SE/GoA	SAN ANTONIO	TX	1.00	1.75	1.87	2.24	1.00	1.00	0.00	0.00	1.01
SE/GoA	SAN BENITO	TX	1.00	2.03	1.12	1.92	1.00	1.00	0.00	0.00	1.10
SE/GoA	SAN LEON	TX	1.00	1.68	1.87	3.64	2.95	2.95	0.00	0.02	1.06
SE/GoA	SANTE FE	TX	0.85	1.49	1.84	2.20	1.00	1.00	0.00	0.00	1.37
SE/GoA	SARGENT	TX	0.73	1.87	1.23	1.81	1.00	1.00	0.00	0.00	1.68
SE/GoA	SEABROOK	TX	0.93	1.50	1.89	2.23	1.00	1.00	0.00	0.00	1.53
SE/GoA	SEADRIFT	TX	0.99	1.57	1.83	2.68	1.67	1.67	0.00	0.00	2.04
SE/GoA	SINTON	TX	0.47	1.49	1.91	2.09	1.00	1.00	0.00	0.00	1.74
SE/GoA	SMITHVILLE	TX	0.96	1.58	2.16	2.27	1.00	1.00	0.00	0.00	1.42
SE/GoA	SWEENY	TX	1.00	1.88	1.20	1.80	1.00	1.00	0.00	0.00	1.02
SE/GoA	TEXAS CITY	TX	0.90	1.66	1.87	3.50	2.76	2.76	0.00	0.00	1.56
SE/GoA	UNKNOWN	TX	1.00	2.04	1.12	1.92	1.00	1.00	0.00	0.00	1.00
SE/GoA	VICTORIA	TX	0.91	1.77	1.45	1.78	1.00	1.00	0.00	0.00	1.22
SE/GoA	WHARTON	TX	0.73	1.44	2.20	1.72	1.00	1.00	0.00	0.00	1.25
SE/GoA	WINNIE	TX	0.97	1.70	1.92	1.75	1.00	1.00	0.00	0.00	1.32
SE/KS	BIG PINE KEY	FL	1.00	1.60	1.96	2.47	1.58	1.58	0.02	0.04	1.91
SE/KS	CUDJOE KEY	FL	0.98	1.74	1.89	2.22	1.42	1.55	0.00	0.01	4.16
SE/KS	DUCK KEY	FL	1.00	1.82	1.92	2.37	1.04	1.04	0.00	0.01	1.09
SE/KS	ISLAMORADA	FL	0.98	1.63	1.95	2.42	1.62	1.67	0.03	0.05	2.50
SE/KS	KEY COLONY BEACH	FL	0.87	1.58	1.85	2.13	1.59	1.95	0.00	0.00	3.02
SE/KS	KEY LARGO	FL	0.87	1.61	1.90	2.32	1.61	1.70	0.24	0.06	3.74
SE/KS	KEY WEST	FL	0.99	1.64	1.75	2.26	1.52	1.57	0.42	0.36	3.49
SE/KS	MARATHON	FL	1.00	1.59	1.95	2.47	1.63	1.64	0.24	0.44	1.98
SE/KS	SUGARLOAF SHORES	FL	1.00	1.60	1.80	1.70	1.00	1.47	0.00	0.00	1.53
SE/KS	SUMMERLAND KEY	FL	0.99	1.69	1.88	2.34	1.33	1.41	0.00	0.01	2.68
SE/KS	TAVERNIER	FL	0.83	1.49	2.03	2.44	1.87	1.87	0.02	0.02	2.09
