# Supplementary material for: Developing indicators of risk to environmental variability based on species dependency in U.S. fishing communities in the Northeast and Southeast Regions
Source: PLoS One. 2025 Dec 30;20(12):e0335034. doi: 10.1371/journal.pone.0335034 (PMC12752975; doi:10.1371/journal.pone.0335034)
Supplement: S3 File — (PDF) [file pone.0335034.s003.pdf]

Supplemental Materials III

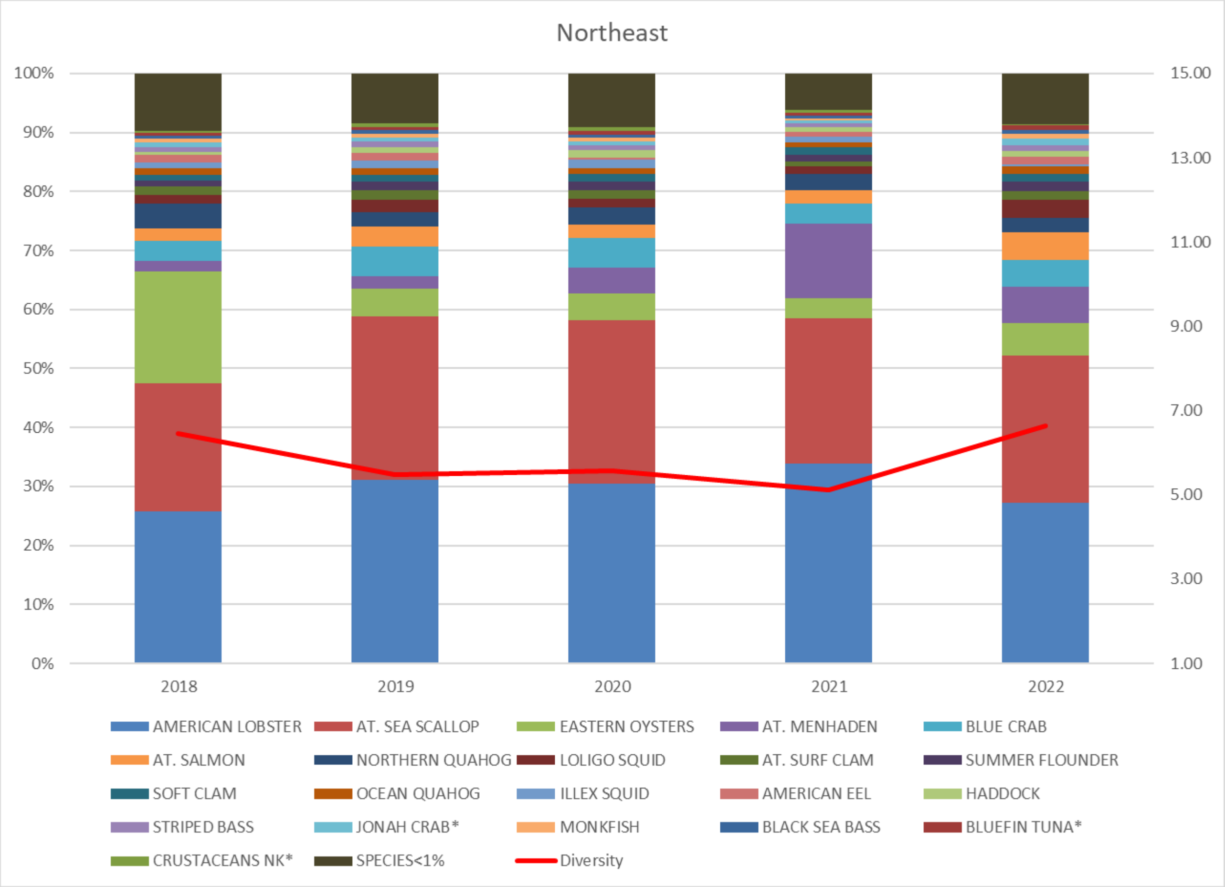

S3 Fig 1. Northeast Region landings composition.

S3 Table 1. Northeast top species CVAs ranked by Total Vulnerability

Species	Temperature	Ocean Acidification	Stock Size/Status	Total Sensitivity	Total Vulnerability
Atlantic Salmon	2.84	1.48	3.84	4	4
Blue Crab	1.64	1.6	2.8	3	4
Eastern Oyster	1.4	3.88	2.56	3	4
Northern Quahog	1.68	3.8	2.28	4	4
Ocean Quahog	2.44	3.88	1.2	4	4
Softshell Clam	1.84	3.48	2.28	3	4
Striped Bass	1.68	1.24	1.96	3	4
American Eel	1.32	1.2	2.72	2	3
Atlantic Sea Scallop	2.44	4	1.8	3	3
Atlantic Surfclam	1.92	3.68	2	3	3
Black Sea Bass	1.4	1.84	2.04	2	3
American Lobster	2.32	2.12	2.28	2	2
Atlantic Menhaden	1.48	1.76	2.44	1	2
Summer Flounder	1.24	1.28	1.96	1	2
Haddock	2.36	1.12	2.28	1	1
Loligo Squid	1.48	1.36	1.84	1	1
Monkfish	2.04	1.08	1.96	1	1
Illex Squid	1.32	1.84	2.08	1	1

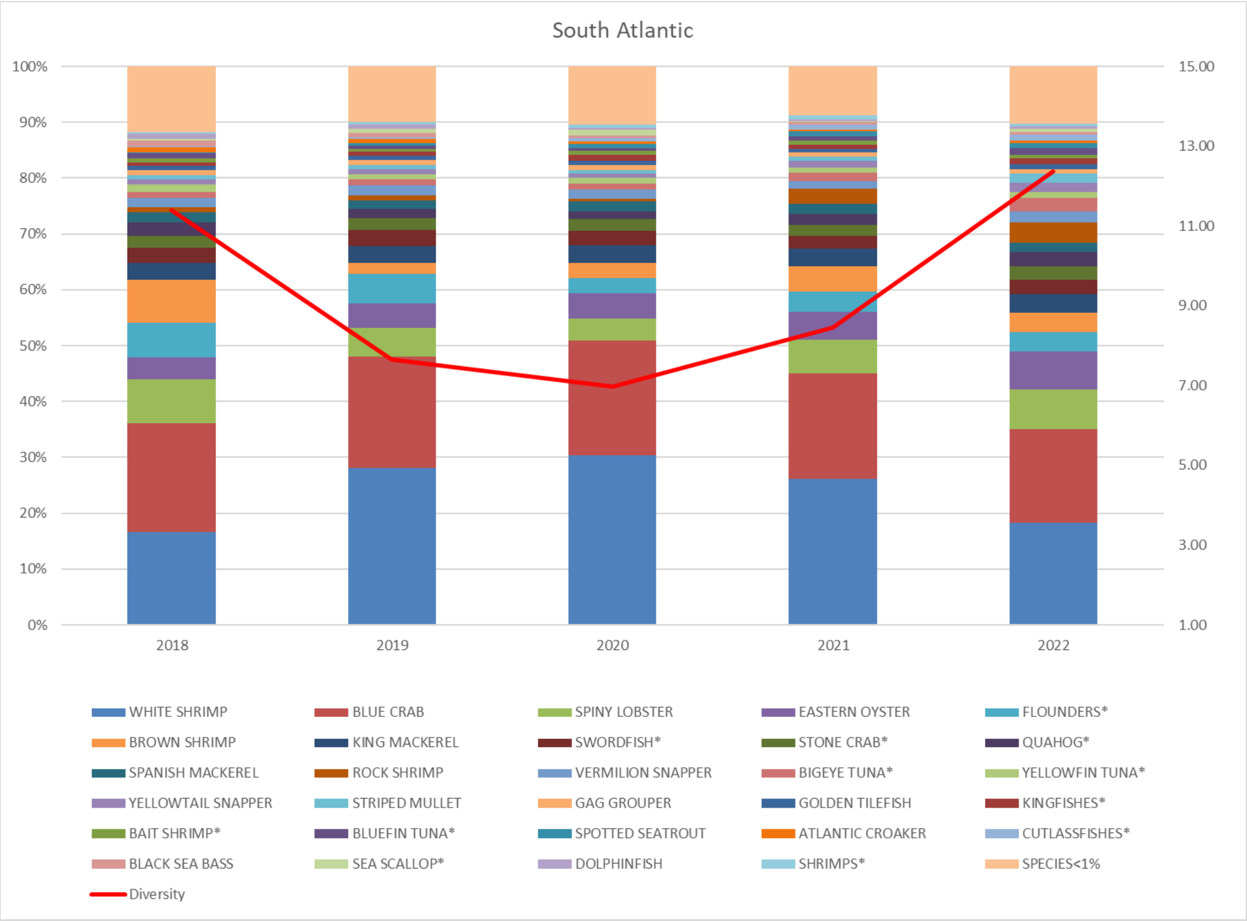

S3 Fig 2. Southeast Region South Atlantic subregion landings composition.

S3 Table 2. Southeast Region South Atlantic subregion top species CVAs ranked by Total Vulnerability

Vermilion Snapper	1.4	2.12	1.6	1	2

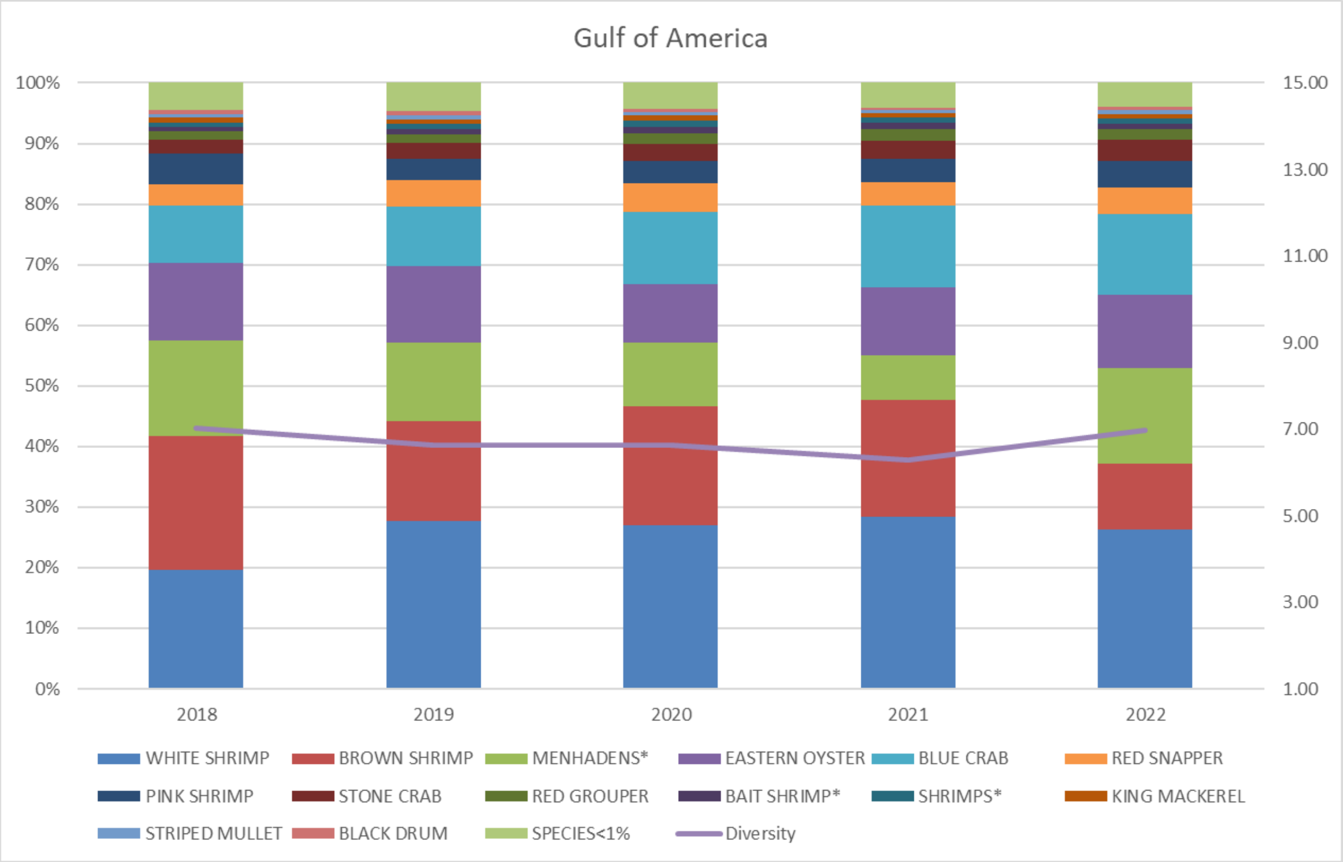

S3 Fig 3. Southeast Region Gulf of America subregion landings composition.

S3 Table 3. Southeast Region Gulf of America subregion top species CVAs ranked by Total Vulnerability

Species	Temperature	Ocean Acidification	Stock Size/Status	Total Sensitivity	Total Vulnerability
Eastern Oyster	1.7	3.7	1.9	3.0	3.0
King Mackerel	1.5	1.5	1.6	1.0	2.0
Red Grouper	2.0	2.0	2.9	2.0	2.0
Red Snapper	1.4	1.5	2.8	2.0	2.0
Black Drum	1.6	2.4	2.2	1.0	1.0
Blue Crab	1.5	2.2	1.8	1.0	1.0
Pink Shrimp	1.8	1.8	1.3	1.0	1.0
Shrimp, Northern Brown	2.0	1.9	1.1	1.0	1.0
Shrimp, Northern White	1.9	1.8	1.2	1.0	1.0
Stone Crab	1.8	2.4	1.9	1.0	1.0
Striped Mullet	1.8	1.6	2.0	1.0	1.0

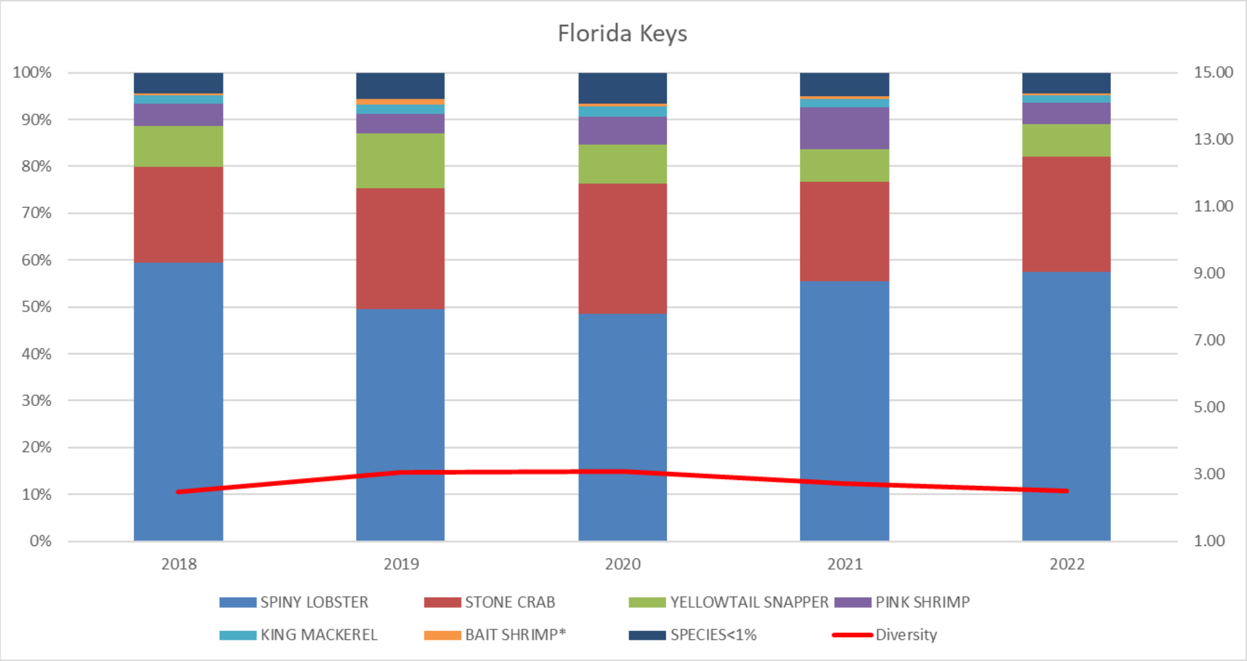

S3 Fig 4. Southeast Region Florida Keys subregion landings composition.

S3 Table 4. Southeast Region Florida Keys subregion top species CVAs ranked by Total Vulnerability

Species	Temperature	Ocean Acidification	Stock Size/Status	Total Sensitivity	Total Vulnerability
King Mackerel	1.5	1.5	1.6	1.0	2.0
Spiny Lobster	1.4	2.6	2.0	2.0	2.0
Northern Pink Shrimp	1.8	1.8	1.3	1.0	1.0
Stone Crab	1.8	2.4	1.9	1.0	1.0
Yellowtail Snapper	1.8	2.0	1.2	1.0	1.0
