# Supplementary material for: Developing indicators of risk to environmental variability based on species dependency in U.S. fishing communities in the Northeast and Southeast Regions
Source: PLoS One. 2025 Dec 30;20(12):e0335034. doi: 10.1371/journal.pone.0335034 (PMC12752975; doi:10.1371/journal.pone.0335034)
Supplement: Attachment — Submitted filename: POne_CEVRI Reviews Round 1.docx [file pone.0335034.s005.docx]

**We would like to thank the reviewers for the comments and suggestions that helped improve the manuscript considerably. We have provided detailed responses to each reviewer’s specific comments below. In addition, we have also made improvements to the figures to increase readability and quality.**

**Reviewer #1:** This paper includes an impressive amount of work and includes a lot of great information about risk and vulnerability to fishing communities and fisheries species on the U.S. Northeast and Southeast. It is also very well written and easy to follow. The addition of scores over time is an important, novel contribution. I do however feel there is more work that could be done to strengthen and clarify this manuscript, mainly in the methods.

**We thank reviewer 1 for highlighting the novel and important contributions of the paper and for the suggestions that have helped us improve the paper. We give specific responses to each question and suggestions below.**

METHODS
1. Use of the term “vulnerability” and no adaptive capacity indicators – the term Total Vulnerability is used but according to line 199, the Total Vulnerability score is only made up of sensitivity and exposure (“considering all 12 sensitivity and 12 exposure factors”) and doesn’t include adaptive capacity. Though the intergovernmental panel on climate change has changed its definition of vulnerability over time, I’m pretty sure in every iteration it includes some form of adaptive capacity. Based on IPCC and other literature, vulnerability is commonly a combination of sensitivity, exposure, and adaptive capacity. Does the Total Vulnerability indicator used include adaptive capacity? If not, why not and should this then be “risk”?

**We have made edits to the paper to clarify the use of the terminology referred to above. The indicators were developed to understand potential risk of a community based solely on their level of dependence on certain species and those species bio-environmental vulnerability as calculated by the Climate Vulnerability Assessments conducted for each region by their respective Fisheries Science Center researchers. The term “vulnerability” should only be employed in this context when referring to those prior assessments. This has been corrected in the revised manuscript. It is outside of the scope of this paper to methodologically account for adaptive capacity - this is a very important aspect that we hope to address in a more encompassing future paper but one that would require a great deal of additional effort.**

Furthermore, the introduction on lines 144-149 talks about the importance of considering social factors in combination with environmental factors and also adaptive capacity in general. Yet, I don’t think any social adaptive capacity is looked at within the metric of vulnerability used in this manuscript. Total species diversity is looked at as a proxy for adaptive capacity, but what about social components that make up these communities? Yes you found lower risk for the South Atlantic compared to Northeast but what about the variation in social factors between these regions that may make one more able to cope then another? Is it possible to look at how social adaptive capacity factors would impact your results? And/or at least discuss?

**Diversity is one aspect widely discussed in the literature as affecting adaptive capacity. The manuscript does not explicitly claim that to be the only factor and this is discussed in the context of literature addressing the relationship between diversity and adaptive capacity. As mentioned above, we believe it is beyond the scope of this paper to include or discuss all aspects that influence a community’s adaptive capacity. Here we focused on the methods used to develop indicators to correlate potential risk and species dependence for communities. We have removed Adaptive Capacity from the keyword list to avoid confusion and have made other edits to increase clarity. In addition we have included a sentence in the discussion to further highlight the value of exploring these relationships in future research:**

***“Future research to consider factors influencing a community’s adaptive capacity in the face of the environmental variations captured by the CEVRI is also a necessary step for further understanding community vulnerability in a more holistic and pragmatic way.”***

2. Lines 182-184 – By using landed value dollars instead of weight, how much does this change what the top species are? Is the high value for certain species potentially due to high value for a rare species and then the top species don’t actually reflect what is most commonly fished for? In other words, would weight actually reflect what is a more common resource across many fishers? For example, Koehn et al. 2022 looked at – “We focused on the top 90% of landed weight, not revenue, to focus on the majority of what is caught in the community (versus a rare but highly valued species caught).”

**The reviewer is correct in thinking that the results would be very different if we did the analyses by weight. We made the decision to use value because that better reflects risk from a socio-economic perspective by assessing potential revenue loss for communities, i.e., if environmental changes impact a community’s ability to land species that are of high value that will translate into a significant loss of revenue for the community. Our methods session includes the following clarification which the authors believe is sufficient justification:**

***“The species’ landed value in dollars was used, instead of weight in pounds, to relate risk to environmental variability to community economic dependence on vulnerable species.”***

**The idea of high value “rare” species is not one that brings concerns for the regions studied. In the passage quoted above, Koehn et al. were interested in ecological risk rather than socio-economic risk. In their discussion of social vulnerability they also discuss their results in terms of revenue:**

***“The majority of communities with 25% or more revenue from a single or multiple salmon species are in the top ten percent of at-risk communities (***[***Fig 3***](https://journals.plos.org/plosone/article?id=10.1371/journal.pone.0272120#pone-0272120-g003)***,*** [***S5 Table***](https://journals.plos.org/plosone/article?id=10.1371/journal.pone.0272120#pone.0272120.s010)***). Most communities with greater than or approximately 20% of revenue from Pacific hake or sablefish were also in the top ten percent of at-risk communities.”***

3. Lines 219-221 – I know that the Total Sensitivity and Total Vulnerability were taken from another reference, but how were these calculated? How were each of the 12 sensitivity/exposure factors combined and then how was exposure and sensitivity combined? The algorithm for this could change the results.

**The references cited for the calculation of the bioenvironmental vulnerability scores (CVAs) have all been peer reviewed and they include details of the methodology and validation strategies used by the authors. We only used the final scores as calculated by the experts in the various studies cited - they have used a well tested methodology to calculate total sensitivity and total vulnerability scores for each species included in the assessment and we have plugged those into the CEVRI formula to calculate the COMMUNITY-level scores for total sensitivity and total vulnerability based on landings composition. We have clarified this in the text by adding the word “community” when referring to the community level total sensitivity and vulnerability.**

4. Figure 2 – Flip the y axis so it goes from 0/<0 at the left bottom corner and reads from low to high from up to down. Not sure why the y axis is flipped and goes from high to low

**The direction of the values in the y axis is intentional so that the figure reads from left to right in terms of lower to higher risk. We believe that makes the figure more intuitive and it has no impact on the information provided.**

5. Lines 238-240 – Why were certain species of importance not included?

**We believe this paragraph in the paper (particularly after the inclusion of the underlined below) makes it clear why not all species with importance for communities were included in the CEVRI analyses:**

***“Although the list of species with CVA scores in the Northeast and Southeast Regions represent a significant portion of the commercial landings in these regions, not all species with regional and local importance were included in the biological assessments, limiting our ability to account for the complete species portfolio for every community. In addition, landings composition varies considerably among different communities in a given region. To ensure that final community scores range between one and four, percent contribution to landings value was calculated based only on the total value of CVA-classified species landed in a community, excluding non-classified species.”***

6. Line 272 – The years 2018-2022 were included, did the beginning of covid show any large changes in fishing catch? Was 2020 significantly different than the other years?

**This is an interesting question but we only used percent contribution of species to total landings and we did not observe a significant difference in terms of relative species contribution with Covid.**

RESULTS
7. Figure 6 has one red community – no red on the legend and what community is this? Why so high for that one community?

**This has been fixed.**

8. Figures 8-10 – combine into one plot with 3 panels?

**Thank you for the suggestion. We will leave this decision to the journal editors.**

9. Lines 332-343 and figures 8-10 – What statistics were used to determine that these trends were significant? Could try quantile regression.

**We used Pearson’s r - That was indicated in the figure and the caption and we have added it to the text for clarity.**

10. I find the community profiles interesting. It does make the paper longer but it’s interesting to draw out these examples. Do most communities fit into similar profiles as the 4 you pulled out?

**Each community has a unique profile that is based on their species portfolio. We share the whole list of communities analyzed and their respective CEVRI scores and other variables in the supplemental materials. The criteria used for community selection is clearly explained in the text:**

***“Four communities representing the different regions and sub-regions studied were selected for profiling. Communities selected displayed a high percentage of landings revenue coming from CVA-classified species and a high value contribution to the regional quotient across the 2018-2022 five-year average (Supplemental Materials I). These profiles exemplify the use of the CEVRI to analyze trends in coastal fishing communities’ level of risk to environmental variability and the relationship between risk levels and landings composition.”***

DISCUSSION
11. Lines 504-505 – You mention how social vulnerability and ocean acidification has been discussed elsewhere. What did that paper find? How would that impact your results given there are probably differences in social vulnerability across the regions you looked at?

**The reference to the Ekstrom et al (2015) paper - which I believe is what the reviewer is referring to - is used to emphasize the relationship we establish in the discussion about community high dependence on species of shellfish which are highly vulnerable to OA. Similarly to our argument, this previous study reached similar conclusions using a different methodology - that communities highly dependent on highly vulnerable species have more to lose as ocean pH decreases. Other factors discussed would not impact our results because we cannot make inferences about factors the CEVRI is not assessing. We acknowledge that there are many factors that affect a community’s social vulnerability but here we are only discussing the factors that correspond to their revenue dependence on species that are at risk to the environmental factors considered in the CVA. We believe these limitations are made clear in our description of the methods used to calculate the indicators.**

12. Lines 518-520 – This is a good point that there may be other environmental factors not considered here that species may be vulnerable to. Have other papers considered these? Also, you note in the methods that Colburn et al. 2016 looked at similar regions – what were their findings compared to yours?

**There are many studies that have considered species vulnerability to a number of different environmental factors, including climate change. However, in this study we are using the scores that were calculated as part of the NOAA Fisheries CVA effort to develop the new indicators presented. It is outside of the scope of this paper to consider all possible factors. The CEVRI can be upgraded in future iterations and can be used in conjunction with other indicators and data to show a more complete picture of community risk and vulnerability - we include this in the discussion:**

***“The community environmental variability risk indicators can also be cross-referenced with other community-level indicators to identify intersections between environmental fluctuations and other factors, such as social and economic vulnerability (NOAA 2024d).”***

**We do not believe that including comparisons between the current analysis and Colburn et al. (2016) will add anything of value to the paper. That paper presents a coarser version of the analysis done in this manuscript and it is published in a peer reviewed journal so the information is easily accessible.**

13. Why do you think the one region’s species diversity vs. vulnerability trend was opposite from the other regions and opposite from what’s expected?

**The primary reason for that is that the region in question has high dependence on fewer species that happen to have low CVA scores for Total Vulnerability. This is addressed in the discussion:**

***“Our analyses also show that the relationship between risk and diversity of landings composition is not always straightforward. Communities in the Gulf of America/Florida Keys sub-region with lower diversity scores also tended to present lower environmental variability risk levels for the factors analyzed. Similarly, the Gulf of America profiled community, Bayou La Batre, AL, scored relatively low in terms of environmental variability risk despite being highly dependent on a small number of shrimp species. While the species in question did not show high vulnerability to the factors analyzed, they may be vulnerable to other environmental fluctuations and risks not considered in our analyses.”***

**Reviewer #2:** Review report
This manuscript entitled “Developing environmental variability risk indicators for fishing communities” presents the development and application of Community Environmental Variability Risk Indicators (CEVRI) to evaluate the vulnerability of U.S. Northeast and Southeast fishing communities to environmental variability. Using commercial landings data (2000–2022) and Climate Vulnerability Assessment (CVA) scores for species, the study aims to assess community-level risk based on dependency on vulnerable species. It is an important and timely contribution, particularly in the context of climate adaptation planning for coastal communities. However, the manuscript suffers from critical methodological, conceptual, and analytical limitations that must be addressed before it can be considered for publication. These include insufficient theoretical framing of “risk,” absence of validation or statistical testing, over-simplified treatment of diversity and adaptive capacity, and vague policy relevance. Please, see my detailed comments below.

**We thank the reviewer for the thorough review of the manuscript and the many suggestions that have helped us to improve it. We provide responses to the specific comments and suggestions below.**

Major comments
Introduction: The manuscript lacks a formal, operational definition of 'risk'. The distinction between risk, vulnerability, exposure, and adaptive capacity is not clearly articulated. Please, define 'risk' explicitly based on existing frameworks (e.g., IPCC), and clarify how CEVRI maps onto these components.

**We believe that what the reviewer means is a better *conceptual definition* of risk - the *operationalization* is provided in the methods and that refers to the indicator developed to assess/measure risk. We have made several edits to the introduction to more clearly define risk in the context of the study:**

***“In this study, we examine community level risk to environmental variability in coastal fishing communities in the Northeast and Southeast U.S. Regions using indicators based on species dependency and their respective bio-environmental vulnerabilities.”***

***“Environmental variability risk for fishing and coastal communities are determined by: 1) their exposure to variability; 2) their sensitivity to change in terms of target species and the ecosystem on which they depend; and 3) their ability to adapt to change (i.e., their adaptive capacity).***

***In this study, we primarily address the second factor described above by using an interdisciplinary approach to develop indicators that assess U.S. Northeast and Southeast Regions coastal fishing communities’ risk to environmental variability and analyze spatial and temporal trends and changes over the period of 2000-2022. To characterize fishing communities according to their environmental variability risk we analyzed commercial landings value composition using species-level Climate Vulnerability Assessment (CVA) scores first developed by Hare et al. (2016) for species considered commercially, recreationally, and ecologically important in the Northeast [...]”***

Lines 182-184: It`s stated that “The species’ landed value in dollars was used, instead of weight in pounds, to relate risk to environmental variability to community economic dependence on vulnerable species.” The use of commercial landings value (rather than biomass or catch volume) as the sole economic proxy for community dependence on species introduces market volatility as a confounding factor, since price fluctuations may not reflect ecological vulnerability. Therefore, a sensitivity analysis to test how this proxy distorts risk scores under price shifts is required.

**We respectfully disagree with this statement. We made the decision to use value because that is a better reflection of risk from a socio-economic perspective since it measures potential revenue loss for communities, i.e., if environmental changes impact a community’s ability to land species that are of high value that will translate into a significant loss of revenue for the community. We are not measuring ecological vulnerability but rather risk to human communities in the face of environmental variability. Using percent contribution to total value in this case is appropriate as a proxy for economic dependence. In addition, while price fluctuations are a pertinent factor, choice of target species is constrained by other factors (e.g., licensing, gear type). Moreover, we use time series and averages to show overall community dependence on species, as opposed to a snapshot. Our methods section includes the following clarification which the authors believe is sufficient justification:**

***“The species’ landed value in dollars was used, instead of weight in pounds, to relate risk to environmental variability to community economic dependence on vulnerable species.”***

**This decision has also been supported by consulting with NMFS economists and it is in line with previous research looking at community risk factors under environmental change, e.g. in Koehn et al. (2022): *“The majority of communities with 25% or more revenue from a single or multiple salmon species are in the top ten percent of at-risk communities (***[***Fig 3***](https://journals.plos.org/plosone/article?id=10.1371/journal.pone.0272120#pone-0272120-g003)***,*** [***S5 Table***](https://journals.plos.org/plosone/article?id=10.1371/journal.pone.0272120#pone.0272120.s010)***). Most communities with greater than or approximately 20% of revenue from Pacific hake or sablefish were also in the top ten percent of at-risk communities.”***

Lines 196-201: Table 1 lists three sensitivity attributes used to calculate Community Environmental Variability Risk Indicators (CEVRI), but this requires clarification. While the original Climate Vulnerability Assessment (CVA) framework includes 12 sensitivity and 12 exposure attributes, the current manuscript employs only three sensitivity attributes. This drastic reduction risks oversimplifying species-level risk assessments, particularly in dynamic environments where multiple stressors interact. For instance, excluding exposure factors (e.g., hypoxia, salinity shifts) limits ecological realism by failing to capture critical threats that compound vulnerability. Please explicitly justify this selective use of attributes in the methodology. If driven by data limitations, acknowledge this constraint and discuss how it may affect the interpretation of results (e.g., underestimating risks for species sensitive to omitted stressors). Additionally, consider addressing whether the retained attributes sufficiently represent the full spectrum of climate impacts or inadvertently bias outcomes toward specific vulnerability pathways.

**The Climate Vulnerability Assessments (CVA) efforts conducted by NOAA Fisheries scientists considered each sensitivity and exposure factor separately for each species, therefore each species has a specific score for each factor (e.g. Sea scallops have a score for their sensitivity to temperature, and another score for the sensitivity to OA). These specific scores were then used by the researchers to create composite Total Sensitivity (all sensitivity scores combined) and Total Vulnerability (all exposure and all sensitivity scores). In our study we took the species scores for the 3 sensitivity factors that relate to environmental variability (temperature, OA, stock size and status) and used these to create community-level scores for these three factors. We are not reducing or oversimplifying risk because we explicitly refer to risk to temperature, risk to OA, and risk based on stock size and status. In addition, we also calculated community-level scores that use the species’ Total Sensitivity and Vulnerability scores therefore accounting for factors beyond environmental variability and stock status in order to capture critical threats that compound community risk (that includes biological sensitivities and different exposure factors that are listed in the extensively cited peer reviewed papers and NOAA technical memos we reference). To avoid the perception that other critical factors are being omitted we have, in addition to citing and providing links to the sources, added the list of all sensitivity and exposure factors included in the Total Sensitivity and Total Exposure scores to the supplemental materials. We thank the reviewer for leading us to realize that we had maintained the text as it has been originally written for the NE region where 12 exposure factors were used and had forgotten to acknowledge that this number varies by region, which we have now added to the text. We have not, however, found it necessary to duplicate all the information provided in the extensive methodologies of the publicly available resources.**

Line 200: I strongly recommend including a comprehensive supplementary table listing all analyzed species with their associated CVA scores, organized by: geographic region, taxonomic group (e.g., hard corals, mollusks, calcified algae), scientific names, and species importance (commercially, recreationally, and ecologically important). This should be accompanied by each species' percentage contribution to total landed value, with special designation for non-CVA assessed species. Crucially, the table should explicitly quantify what percentage of total landings were excluded from analysis per region/community due to lack of CVA scores. Providing these details would serve four key purposes: first, it would enhance methodological transparency by making the study's taxonomic coverage explicit; second, it would enable more robust assessment of regional and taxonomic vulnerability patterns; third, it would facilitate meaningful comparative analyses with other studies; and fourth, it would significantly improve the reproducibility of the findings. The absence of such fundamental data currently limits readers' ability to evaluate potential biases introduced by species exclusions or taxonomic coverage gaps.

**Each publication for their respective region cited in the manuscript includes the full list of species, with their taxonomic groups and scientific names. These publications include species profiles with very complete information and the criteria used to score the species. Since all this information is publicly available either through the NOAA website or open access peer reviewed journals, we do not think it is necessary to include that information in this paper. We include in the table provided in the supplemental materials 2 the 5 year average percent contribution of unclassified species which covers the regional data mapped in the paper. We have added a sentence to the methods to make that explicit. The communities that were chosen to be profiled averaged above 80% or more during the whole time series (2000-2022) as indicated in the methods section:**

**“Select communities were also analyzed for longitudinal trends to exemplify the application of the CEVRI for further community-level analyses and profiling. For these analyses, communities with relatively high dependence on classified species for value landed (80% or more) were selected and a time series (2000-2022) for each CEVRI was plotted along with regional level scores for comparison purposes.”**

**The CVA or similar bio-environmental assessment by species is a necessary pre-requisite for reproducing the analyses conducted in this study. The methods used to conduct CVAs are extensively covered in the literature, have been validated by implementation in multiple US regions and are publicly available through a NOAA website dedicated to the CVAs:** [**https://www.fisheries.noaa.gov/national/climate/climate-vulnerability-assessments**](https://www.fisheries.noaa.gov/national/climate/climate-vulnerability-assessments) **. Reproducibility of the methods presented here require that the CVA steps and methods be reproduced based on the appropriate literature first.**

Lines 214: In the CEVRI equation, the manuscript should clarify whether species were weighted equally in the calculations or if adjustments were made to account for the disproportionate importance of high-value or dominant species. This distinction is critical for proper interpretation, as equal weighting could mask vulnerability hotspots concentrated in economically significant taxa.

**The objective of the indicator is to assess risk to environmental change to a community based on how much each species contributes to value and multiply that by its 1-4 CVA score which results in a 1-4 community score that takes into consideration the landings composition and the different CVA scores, i.e., species vulnerabilities to the 3 environmental variability factors and then total scores for sensitivity and vulnerability. So the goal IS to weight by contribution so that if a community depends 100% economically on a species highly vulnerable to OA, say (OA CVA = 4) that community would also have a score of 4, meaning that community is at high risk for decreases in pH.**

Lines 212-216: The presentation of CEVRI scores as point estimates without confidence intervals or uncertainty measures (Figures 11-12) limits interpretation of result robustness. I recommend incorporating statistical uncertainty quantification through methods like bootstrap or Monte Carlo simulations, particularly since two key inputs introduce variability: (1) expert-derived CVA scores with undocumented weighting criteria, and (2) fluctuating market prices. A sensitivity analysis testing how CEVRI ranks respond to variations in these inputs would greatly strengthen the analysis by identifying which components drive most uncertainty. This is especially important given the policy implications of vulnerability assessments, where understanding score stability across plausible parameter ranges is crucial for decision-making. For a robust bootstrap analysis to quantify uncertainty, I recommend consulting Burton et al. (2023), who demonstrate applicable methods for propagating uncertainty in assessment of overall climate vulnerability (page 7).

**It is not possible to calculate uncertainty here because there is no data distribution to be analyzed. The yearly score is simply the sum of the multiplied CVA score (which is a constant by each species by factor) by their contribution to value for that given year. The expert derived uncertainty was accounted for in the methods used by the researchers involved in the scoring assessments (publicly available information), the final scores generated by that process are ordinal values, i.e., ranks, ranging from 1 (low) to 4 (very high). Market price fluctuations affect the revenue generating value of a species, but that will be reflected in the percent contribution to value for each species in any given year. In other words, if the price decreases for a particular species that *could* result in lower contribution of that species to total value which will affect the CEVRI score for that given year (risk will go up or down depending on the CVA score for that species and the relative contribution of the other species landed). This explains why the CEVRI is best employed for understanding time series or based on an average of recent years, rather than a snapshot. The ability to look at trends showing, for instance, that a community is becoming more dependent as time passes on a highly vulnerable species or that based on the landings composition characteristics of the past 5 years, a community’s portfolio shows high risk to certain environmental variability factors is what we are interested in and we are confident that the manuscript is achieving this goal, particularly after the revisions based on reviewers’ comments.**

Lines 219-222: The methodology for calculating total sensitivity and total vulnerability requires clarification, as an apparent inconsistency exists between the described approach and the implementation. While the text indicates that 12 sensitivity and 12 exposure factors were used (Lines 198-199), the CEVRI values were ultimately derived from only three core sensitivity attributes. To resolve this discrepancy and ensure methodological transparency, three key clarifications are needed. First, the relationship between the 12 factors and the three CEVRI attributes should be explicitly stated, explaining how the broader set of factors maps onto the simplified framework. Second, the mathematical formulation used to aggregate the factors and calculate the total sensitivity and vulnerability metrics should be provided, including all relevant equations or decision rules. Third, the weighting scheme applied to the factors that requires clarification - whether equal weights were used throughout or if differential weighting was employed, along with the rationale for this choice. Providing these details would significantly strengthen the methodological rigor and reproducibility of the analysis.

**This comment demonstrates a lack of understanding of the methodological approach and revisions have been made to hopefully improve clarity. The CEVRI analyses are not solely based on the 3 sensitivity attributes, i.e., we do not simplify the framework, we simply use the individual species scores generated for each one of those factors. We use those three *in addition* to the Total Sensitivity and Total Vulnerability scores that were also generated as part of the CVA framework by species by considering all sensitivity and all sensitivity and exposure factors, respectively. We isolated those 3 in addition because they relate to environmental variability and community adaptation more closely, and are thus of particular interest to our analysis. The community total sensitivity and total vulnerability scores reflect the complete set of factors analyzed during the CVA process, thus providing a complete picture. We did not use any mathematical formulations to aggregate factors - this was part of the CVA methodology which is cited and can be accessed publicly.**

Line 243: The limited species coverage (71-82 per region) may bias risk assessments by excluding locally important taxa. Please: quantify excluded species, discuss potential impacts on results (e.g., for specialized fisheries), and consider proxy methods for unassessed species (e.g., taxonomic relatives). A supplementary table listing all analyzed species with their CVA scores would improve transparency.

**The limited species coverage DOES bias risk assessments. That is one of the most significant weaknesses of the data. We do address this by making it clear that we are only using CVA classified species in the calculation of CEVRI and by 1) presenting the percent classified species for each community represented in the spatial analysis in supplemental materials; 2) only choosing communities with high percent contribution to profile and 3) marking non-classified species with an asterisk in the landings composition graphs. While this is indicated as a weakness of the current available data, this in no way invalidates the methods developed and the results presented for the profiled communities. In addition, the CVAs are meant to be updated and species can be added in that process to increase the percent of species that are considered important at the community level. In fact, that is one of the valuable contributions of this work: it provides another method for determining species importance and updating species lists for future CVA iterations (this is currently happening in the NE region and the analyses presented here have been used to propose the addition of species that have relatively high importance for a number of communities in the region). Working closely with natural scientists to develop accurate proxies and/or update the bioenvironmental information is a valuable recommendation for a future project to improve iterations of the CEVRI.**

Line 249: The manuscript's use of Simpson’s Reciprocal Index as a proxy for adaptive capacity warrants further discussion given its limitations in capturing key ecological dimensions of resilience. While species diversity metrics provide some insight into portfolio effects, the current approach overlooks two critical factors that mediate true adaptive capacity: functional redundancy (whether landings comprise species with similar ecological roles) and trophic breadth (the range of feeding strategies represented). This is particularly important because a fleet landing diverse but uniformly climate-vulnerable taxa (e.g., multiple mollusk or shrimp species) would appear resilient under the current metric while actually facing compounded risks. I recommend the authors explicitly address this limitation in the discussion, acknowledging how the "diversity equals resilience" assumption may break down when portfolios contain taxonomically clustered, similarly sensitive species. A valuable extension would be developing a weighted diversity index that incorporates species-specific vulnerability scores, where the contribution of each species to the overall index is adjusted by its climate sensitivity. This refinement could better distinguish between truly resilient portfolios (diverse across vulnerability categories) and pseudo-resilient ones (diverse but uniformly vulnerable). At minimum, the discussion should explore how the current metric might overestimate adaptive capacity in regions specializing in taxonomically narrow but species-rich fisheries, with potential examples from the study system.

**We are not trying to assess ecological resilience but rather socio-economic risk based on a fishing community's revenue dependence on species of low/high bio-environmental vulnerability. Therefore, what we are interested in IS portfolio effects and the well established relationship between that and adaptive capacity in fishing communities as per the literature we cite on this topic on the third paragraph of the discussion. While we address the importance of compounded risks in the discussion, is it not possible nor the intention of this indicator to account for all or most of the factors affecting a community risk to environmental factors. We specifically address that the relationship is not straightforward and we exemplify that with the SE communities chosen for profiling which are characterized by low diversity AND low risk to the environmental variability factors investigated in this study. We acknowledge that while they may appear low risk, these communities are highly dependent on 1 or 2 species with low vulnerability but if those species experienced impacts from other factors, such as disease or habitat loss, for example, that could be detrimental to the community - in the fourth paragraph of the discussion we use the “gilded trap” example and Long Island Sound to further illustrate that idea. The key here is that we are looking at multiple environmental variability factors, so while a community may be highly vulnerable to OA it can be more moderately vulnerable in terms of its *total* vulnerability because that will capture additional factors. The reviewer's suggestion of “developing a weighted diversity index that incorporates species-specific vulnerability scores, where the contribution of each species to the overall index is adjusted by its climate sensitivity” is not entirely clear but it sounds like it is describing precisely what the CEVRI does.**

Line 268-270: The use of five-year aggregated data for indicator time series raises concerns about temporal resolution adequacy in capturing climate vulnerability dynamics. While this approach may smooth interannual variability, it risks obscuring three critical elements: first, the immediate impacts of acute environmental or economic shocks that often drive rapid changes in fishery systems; second, the potentially important short-term adaptive responses communities employ following such disturbances; and third, early warning signals that typically manifest at finer temporal scales before establishing longer-term trends. This resolution limitation is particularly problematic for climate vulnerability assessments where timely detection of emerging risks is paramount for effective adaptation planning. I strongly recommend the authors either analyze the data at annual resolution to preserve these critical signals or provide a robust methodological justification for the five-year aggregation choice - for instance, by demonstrating through sensitivity analyses that key patterns remain detectable at this timescale. A comparison of annual versus aggregated trends in selected high-variability regions would significantly strengthen the manuscript by either validating the current approach or revealing important masked dynamics. Such validation seems especially warranted given that many climate adaptation policies operate on annual decision cycles where missing short-term vulnerability fluctuations could lead to suboptimal management interventions.

**The CEVRI does not account for environmental variability itself but rather for community risk to environmental variability factors considered in the CVA - based solely on their revenue dependency on climate-vulnerable species. The 5-year average is a way to minimize the effect of a yearly spike or dive in any given species’ contribution to value. and provide a more accurate reflection of a community’s recent portfolio of species in order to generate scores that reflect that community risk to, say, decreases in pH or increases in temperature. For example, New Bedford MA’s high dependency on scallops for the most recent years of data indicates that, generally speaking, that community is currently at high risk to lose revenue if the pH decreases considerably -- due to the resultant impacts on scallop populations. A snapshot, i.e., single year score, could underestimate or overestimate species contributions generally. The yearly scores are helpful to show trends, however. They represent 2 potential applications of the CEVRI - time series and spatial visualization. Also important to stress is that the only thing that changes annually is the contribution of species to revenue - species CVA scores are static and can only change if the CVA is updated.**

Policy relevancy: The manuscript’s policy relevance is significantly undermined by the absence of actionable guidance for translating CEVRI scores into tangible management interventions. While the framework is presented as a tool for climate adaptation, it does not clarify how stakeholders—such as fishery managers, disaster relief agencies, or conservation planners—should operationalize these vulnerability rankings in practice. For instance, there is no discussion of how CEVRI could inform dynamic quota adjustments during climate anomalies, prioritize infrastructure upgrades in high-risk communities, or trigger preemptive disaster relief for regions crossing specific vulnerability thresholds. This gap is compounded by the lack of empirical validation against real-world outcomes (e.g., historical fishery collapses, post-disaster recovery rates), leaving uncertainty about whether CEVRI scores reliably predict risks or merely reflect theoretical constructs. Furthermore, the oversimplified use of three sensitivity attributes (versus the original CVA’s 12) and exclusion of critical exposure factors like hypoxia or salinity shifts risks misrepresenting species-level vulnerabilities, particularly in ecosystems facing multi-stressor interactions. These methodological choices, combined with unaddressed data gaps (e.g., unreported landings, unvalidated expert judgments), raise questions about the framework’s ecological realism and its capacity to inform place-based management. To bridge these gaps, the authors must explicitly map CEVRI outputs to existing policy frameworks and provide concrete examples of how scores could guide decisions like MPA expansions, parametric insurance schemes, or species-specific harvest controls. Without this translational discussion and validation, CEVRI risks remaining an academic exercise rather than a tool for building climate-resilient fisheries.

**This comment stems partially from a misunderstanding of the methodological approach and goals of the CEVRI - many of which the authors hope have been addressed by revisions to the manuscript and extensive explanations above. To reiterate, the CEVRI is limited to showing potential risk to a fishing community based on its revenue dependency on species and their respective vulnerability to specific environmental variability factors. We did not oversimplify the CVA data, we simply used 3 sensitivity factors in isolation IN ADDITION to the composite scores for total sensitivity and vulnerability generated by the experts through validated and publicly available CVA efforts. There are NO unreported landings - we explain this as a weakness of the calculation since some species landed were not included in the CVA but the percent contribution of CVA species is included for the 5-year averages on the map and the community profile data includes all species (non-classified have an asterisk); expert judgements are not unvalidated - they are part of the extensively peer reviewed and publicly available CVA efforts that are clearly outlined on the NOAA website and in open access citations in the paper. The authors have full confidence in the work conducted by the CVA efforts, which were first published in 2016 and continue to be extensively referenced. We also appreciate the reviewer’s suggestions to strengthen the policy relevance and have used some of these ideas to improve the paper. However, the information provided by the CEVRI alone is not sufficient to guide the type of complex decisions that the reviewer has suggested. Moreover, we feel a discussion of the role it could play in these types of decisions is outside the scope of this paper.**

Methods of analysis: The manuscript lacks empirical validation of the CEVRI framework against observed socio-economic or ecological outcomes, such as historical fishery collapses, livelihood disruptions, or species population declines. Without cross-verification with real-world data (e.g., comparing CEVRI scores to post hoc vulnerability trends), it remains unclear whether these indicators reliably predict risks or offer actionable insights for decision-makers. This omission significantly limits the framework’s credibility for policy or management applications. To address this gap, the authors should either: validate CEVRI scores against historical case studies in the study regions, or compare results with independent vulnerability assessments (e.g., IUCN Red List trends, fishery closure records). At minimum, the discussion must explicitly acknowledge this limitation and outline plans for future validation, as unverified indices risk misdirecting climate adaptation efforts.

**While the authors appreciate the suggestion to increase validation of the indicators proposed, the goal of this first publication of the CEVRI does not go beyond proposing a method to increase understanding of a community’s potential risk to specific environmental factors considered in the CVA process based solely on landings composition and potential loss of revenue. The authors agree that a fruitful next step would be to analyze the CEVRI scores in relationship to environmental variability but this is a much larger effort and one that is beyond the objectives of this paper. While the urge to use one indicator to address and provide solutions to all of management problems is understandable, we see the development of the CEVRI as the beginning of many interesting research questions and potential applications that can stem from future collaborations. In addition, the authors have tested the value of and interest in this kind of analyses through presentations during fishery management council meetings in the Northeast Region, where we have received helpful and positive feedback, particularly in relation to their use in the councils’ ongoing development of risk analyses. In the manuscript, we provide a realistic overview of the applications in the discussion by highlighting 1) the indicators’ potential to guide considerations for catch diversity limitations stemming from licensing and other regulations, 2) its value as a communication tool for the need to address broader challenges that may involve competing uses of the oceans, 3) its potential to complement existing indicators exploring other facets of community vulnerability and resilience, as well as 4) CEVRI’s value as a methodological model for the development of future indicators.**

Minor comments
Figure 1: The methods should clarify how communities were geographically defined—whether by municipal boundaries, fishing port locations, or dealer reporting addresses. This distinction is critical for interpreting spatial patterns in the analysis. I recommend including a map showing dealer coverage and their respective service areas to help visualize potential data limitations. Additionally, please discuss how landings aggregation might affect the results. For instance, dealers in strategic locations (e.g., Atlantic Beach, FL) may source from multiple states, creating mismatches between reported dealer locations/address and actual fishing grounds. This could artificially inflate or distort the apparent vulnerability of certain communities. Addressing these nuances would strengthen the study’s transparency and highlight important limitations for management applications.

**We have added information to more explicitly explain in the methods section how the data was aggregated for each region and acknowledge data limitations:**

**“*The data used for the development of the community-level indicesCommunity Environmental Variability Risk Indicators (CEVRI) consists of landed value reported to NOAA Fisheries by dealers and aggregated at the community level by port of landing for the Northeast region and dealer address for the Southeast region. The use of different aggregation criteria for the two regions are a consequence of limitations in the data reported by the responsible authorities. For the purposes of our analyses, communities are geographically defined by the U.S. Census Bureau Federal Information Processing Standard (FIPS) “Census Place” codes associated with a port or dealer’s address city. The use of landings value as reported by dealers by location was used to reflect value likely to be retained in the community, thus indicating their revenue dependency on different species.*”**

**Unfortunately, it is not possible to provide an accurate map of a dealer service area as that information is not available and dealers may receive products from a multitude of places at any given time. The argument behind using dealer reported data is that this would better reflect value that is being retained in a community and the fact that most of the highly engaged and reliant fishing communities typically have a high volume and value being landed and dealt in said communities. This approach is naturally imperfect and compounded by data reporting limitations. We are aware of the challenges highlighted by the reviewer and have debated them extensively over the years.**

Line 224. Figure 2: For consistency with Figures 3 and 4, please either label the categories directly in Figure 2 (i.e., "1: Low", "2: Medium", "3: High", "4: Very High"), or include this classification system in the figure legend, explicitly defining what each CVA score represents.

**We have added the information to the figure legend.**

Discussion: The CEVRI assessment may be affected by data gaps, particularly regarding small-scale or illegal fisheries activities. Since landings are often attributed to dealer locations rather than actual fishing grounds, this could distort spatial vulnerability patterns. Please discuss how these data limitations might influence the accuracy of CEVRI scores and their interpretation for management purposes.

**The authors acknowledge that data gaps and impacts of IUU fisheries is a constant challenge in any analyses involving fisheries data. This is a universal problem and one that using different data aggregation criteria would unfortunately not address. We also understand that different methodological approaches would result in different outcomes, e.g. using port of landing v. homeport, and volume in pounds. However, our decisions are based on decades of extensive work utilizing the data sources available and limited by data reporting constraints and we believe that the information provided in the methods section, particularly after revisions, are sufficient for the reader to understand what data was used and the justifications for using said data.**

Discussion: While CEVRI effectively captures exposure and sensitivity components, it currently does not account for community adaptive capacity factors such as alternative livelihoods or policy support systems. I recommend adding a brief discussion acknowledging this limitation and considering how incorporating adaptive capacity metrics might alter vulnerability rankings.

Discussion: The CEVRI framework focuses on climate-specific vulnerabilities without addressing concurrent stressors like overfishing, pollution, and habitat degradation. Please include discussion of how these anthropogenic pressures might interact with or amplify climate impacts, and whether their exclusion might lead to underestimation of true vulnerability in certain ecosystems or communities.

**We have added the following sentences to the last paragraph of the discussion to address both comments above:**

***“While the CEVRI effectively captures risk to specific environmental variations, communities are affected by a number of concurrent stressors that may compound the effects of the disturbances considered, such as pollution and habitat degradation. Future research considering these added stressors and potential interactions between them and those considered in the CVAs would further elucidate risks to community well-being. In addition, research considering factors influencing a community’s adaptive capacity in the face of the environmental variations captured by the CEVRI is a necessary step for further understanding community vulnerability in a more holistic and pragmatic way. Comparing the assessment of potential risk as indicated by the CEVRI to the factors that may help a community minimize said risks will provide valuable information about policy and management priorities in the face of specific environmental disturbances.”***
